# Vacancy enhanced Li, Na, and K clustering on graphene†

**DOI:** 10.1039/d5se00130g

**Published:** 2025-04-16

**Authors:** Jonathon Cottom, Qiong Cai, Emilia Olsson

**Affiliations:** a Advanced Research Center for Nanolithography Science Park 106 Amsterdam 1098 XG The Netherlands; b School of Chemistry and Chemical Engineering, University of Surrey Guildford GU2 7XH UK; c Institute of Theoretical Physics, Institute of Physics, University of Amsterdam Science Park 904 Amsterdam 1098 XH The Netherlands k.i.e.olsson@uva.nl

## Abstract

The formation of metallic dendrites during battery cycling is a persistent challenge for alkali metal-ion batteries, reducing cycle life and posing safety risks. Although surface defects are often implicated in inhomogeneous metal nucleation, the atomic-scale mechanisms by which they promote metal clustering and subsequent dendrite formation remain poorly understood. Here, we use first-principles calculations to investigate how carbon monovacancies (V_C_) influence the clustering behaviour of alkali metals (Li, Na, and K) on graphene – a common basal-plane motif in graphite, hard carbons, and graphene-based anodes. Clusters of Li, Na, and K of varying size (M_*n*_ for *n* ∈ {1–12}) are characterised on pristine and defective graphene to understand their stability. On pristine graphene, cluster formation is hindered for Li due to the instability of small clusters (*n* ≤ 3) and significant Li–Li repulsion, and suppressed for K due to weak K–K binding and its larger ionic radius. In contrast, Na exhibits spontaneous clustering, suggesting a higher propensity for dendrite formation even in the absence of defects. The introduction of a V_C_ dramatically alters these trends: it stabilises small (*n* ≤ 3) clusters across all three metals by enhancing binding strength with the surface and modifying charge localisation. For Li, the vacancy overcomes the barrier to early-stage nucleation; for Na, it promotes growth at even lower metal loadings; and for K, clustering becomes locally favoured albeit only for the smallest cluster sizes (*n* ≤ 3). These results clarify the defect-facilitated pathways to metal clustering, offering atomistic insight that can inform the development of more dendrite-resistant carbon architectures.

## Abstract 2

The formation of metallic dendrites during battery cycling is a persistent challenge for alkali metal-ion batteries, reducing cycle life and posing safety risks.

## Graphical abstract

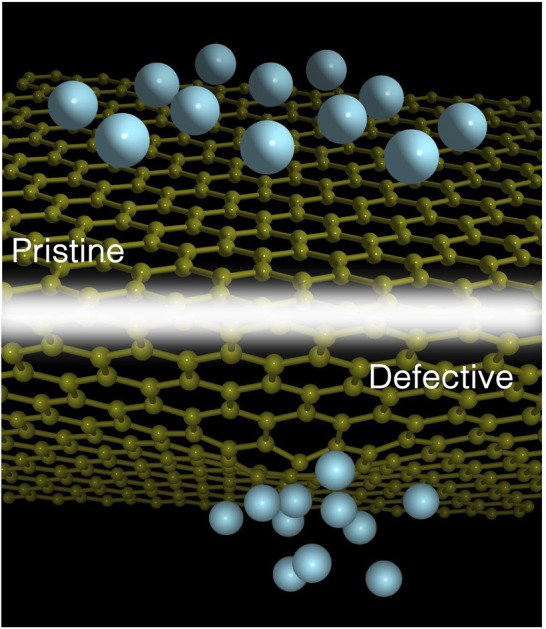

## 1. Introduction

As human society seeks sustainable energy solutions, the development of efficient storage technologies has become pivotal in reducing the dependency on fossil fuels. Lithium-ion batteries (LIBs) have long been central to this effort due to their high energy density and efficiency across a wide range of applications.^1–3^ However, with growing demand straining global lithium (Li) resources, exploring viable alternatives and complementary technologies is essential.^4–9^ Sodium (Na) and potassium (K), situated alongside lithium in the periodic table, offer promising pathways toward cost-effective, large-scale energy storage systems that can alleviate pressure on lithium supplies. Sodium-ion (NIBs) and potassium-ion batteries (KIBs) are emerging as feasible options for applications where weight constraints are less critical, enabling LIBs to be prioritized for weight-sensitive applications.^10–13^

Alkali metal-ion batteries (MIBs), including lithium, sodium, and potassium, share common design elements: a layered cathode, a liquid electrolyte, and (typically) a carbon-based anode. A wide range of carbonaceous anode materials—graphite, graphene, hard and soft carbons, and carbon nanotubes—have been implemented across these systems with varying degrees of success, each offering distinct surface morphologies, porosities, and electronic properties.^14–17^ In parallel, carbon materials are increasingly being explored as interlayer components in solid-state battery architectures, where they facilitate alkali metal plating and stripping while improving mechanical contact with solid electrolytes.^12,18,19^ Despite their morphological diversity, these materials exhibit sp^2^-hybridized frameworks of six-membered carbon rings, forming planar or quasi-planar basal surfaces reminiscent of graphene.^16,18,20–24^ This structural commonality establishes graphene both as a candidate anode material and as a prototypical model for investigating alkali metal interactions on carbon surfaces.^25–31^

The electrochemical performance of MIBs remains constrained by persistent challenges related to stability and cycle life, particularly under fast-charging conditions.^8,12,32,33^ A critical bottleneck is the uncontrolled nucleation of metal at the anode, which initiates dendritic growth and irreversible plating.^34–38^ These filamentary structures, resulting from inhomogeneous deposition at the anode–electrolyte interface, pose severe safety risks through potential short-circuiting.^16,19,39,40^ Although solid-state batteries (ASSBs) were conceived to suppress dendrite formation by replacing liquid electrolytes with mechanically rigid solids, Li and Na dendrites continue to emerge in practice, limiting commercial viability despite the appeal of high energy density and improved safety.^18,19,41–47^ Recent evaluations of solid-state Na systems suggest that hard carbon remains the most practically deployable anode material due to its interface compatibility and cycling stability.^48^ These persistent issues point to an incomplete understanding of early-stage alkali metal deposition and motivate atomic-scale studies of nucleation and clustering on representative carbon surfaces.

Graphene, due to its exceptional electrical conductivity, chemical inertness, and structural similarity to the basal planes of graphite and hard carbon, serves as a relevant and widely adopted model for carbon-based battery anodes.^22,25,26,28–30,49,50^ While pristine graphene exhibits weak interactions with alkali metals (Li, Na, and K), intrinsic defects such as carbon vacancies (V_C_), Stone–Wales rearrangements, and extended grain boundaries substantially alter local electronic and chemical reactivity.^22,51–53^ These defects introduce localized states near the Fermi level, promoting partially covalent metal–carbon bonding and enabling the nucleation of metal clusters.^51,52,54–56^ A range of defect and structural modifications have been explored as avenues for tuning alkali metal adsorption, including double vacancies, hydrogen-vacancy networks in graphane, and substitutional doping.^29,30,50^ These strategies further underscore the broader relevance of defect engineering for tailoring metal–surface interactions in graphene and structurally analogous carbon anode materials.

Previous work has investigated the interactions of single alkali metal atoms (Li, Na, and K) with both pristine and defective graphene within MIBs.^29,56–59^ These studies highlighted the important role played by intrinsic defects, particularly carbon vacancies (V_C_), in single-ion binding and migration, identifying V_C_ as a primary source of irreversible capacity loss.^11,53,60,61^ The metal–vacancy (M–V_C_) binding is dominated by the strong interaction between the alkali metal and the unsaturated carbon dangling bonds, a feature common to all carbon vacancy-containing defects provided one or more dangling bonds remain.^49,59^ Such localized interactions significantly enhance the stability and reduce the mobility of adsorbed metal atoms. Furthermore the V_C_ forms the simplest building block of many important defect clusters with other vacancies in the form of di-vacancy and extended defect pores and with hetero-atoms such as (N_C_)_*n*_V_C_, (O_C_)_*n*_V_C_ and as a result of doping. Other computational studies on lithium clustering demonstrated that small Li clusters adsorb more favorably on graphene compared to bulk Li metal surfaces,^54^ exhibiting concentration-dependent binding energies that influence charging behavior.^62^ Additionally, lattice-gas cluster expansion analyses have explored the equilibrium stability of Li coverages on graphene.^63^ Despite these insights into lithium, corresponding studies addressing Na and K clustering remain limited, leaving significant gaps in understanding their nucleation behavior on graphene and motivating further systematic investigation.

Liang *et al.*^51^ conducted a comparative study on Na clusters, showing that Na weakly adsorbs on pristine graphene but that double vacancies significantly enhance Na adsorption—a result aligned with our previous findings on defect-enhanced Na storage.^13,49,58,59^ Collectively, these studies offer insights into the deposition and clustering of Li on graphene, though several questions remain. Experimental investigations into metallic Li nucleation on basal-plane graphene, for instance, indicate that the pristine surface is lithiophobic, with nucleation barriers too high to overcome,^52^ whereas basal plane defects are believed to serve as nucleation sites, rendering the surface lithiophilic.^52^ In practical MIB applications, such defects may arise from synthesis conditions or cycling-induced wear, linking them to suboptimal performance and non-ideal behavior. However, the role of defects remains largely undefined, posing an open question as to what drives Li and Na clustering tendencies, while K demonstrates no such effect.

This study systematically assesses the impact of a single vacancy on Li, Na, and K clustering behavior. Through density functional theory (DFT) calculations, we provide atomic-scale insights into M nucleation and cluster growth on graphene, for both pristine and vacancy systems. This approach offers a focused test case to elucidate the critical role of vacancy defects in M-clustering, clarifying the link between surface and cluster morphology and the propensity for dendrite formation.

## 2. Methodology

All density functional theory (DFT) calculations were performed spin-polarized using the CP2K code^64–68^ at the Γ-point, employing DZVP-SR-MOLOPT basis sets^65^ for valence electrons and GTH pseudopotentials^69–71^ for core electrons. A plane-wave cutoff of 750 Ry and a relative cutoff of 60 Ry were used, yielding total energy convergence within 0.1 meV per formula unit. Initial defect-free relaxations were carried out on a 308-atom graphene supercell, generated *via* an 11 × 7 orthohexagonal expansion of the primitive cell (see Fig. S1 in the ESI†). For the pristine cell, both the in-plane lattice vectors and atomic positions were fully relaxed using the quasi-Newton BFGS algorithm.^72–75^ In defect-containing calculations, only the atomic positions were optimized. A vacuum spacing of 25 Å was applied in the out-of-plane direction to eliminate spurious interlayer interactions.^66,68,76^ The Perdew–Burke–Ernzerhof (PBE) functional^77,78^ with D3-BJ dispersion corrections^79–83^ was used, consistent with previous studies.^13,24,49,58,59,84,85^ Geometry optimizations were deemed converged when the total energy changed by less than 1 × 10^−7^ eV and the maximum atomic force was below 0.005 eV Å^−1^.

Using the lowest energy M adsorption sites previously identified in ref. 49, the lowest-energy cluster configurations were determined *via* a stepwise sampling scheme.^86^ Starting with a single metal atom (M_1_), atoms were sequentially added to form each M_*n*+1_ cluster from M_*n*_. To efficiently identify sites where a significant M–M interaction occurs as a prerequisite for cluster growth, any incremental interaction energy greater than −0.05 eV is treated as negligible and therefore non-interacting with respect to cluster growth. In effect, for a given neighboring site, if Δ*E* ≥ −0.05 eV, the corresponding binding interaction is considered statistically insignificant, as this value is on the order of (or below) twice the thermal energy at ambient conditions (*k*_B_*T* ≈ 0.025 eV). Consequently, only sites exhibiting Δ*E*_pair_ ≤ −0.05 eV are included in the initial sampling. In practice the search space is constrained to those sites that are predicted to result in either lateral or vertical growth of the cluster. The total number of configurations considered at each iteration is given by
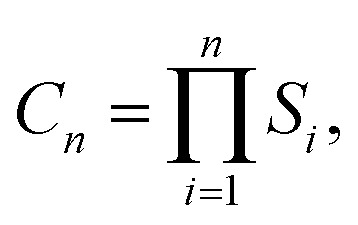
where *S*_*i*_ denotes the set of available neighboring sites for the *i*-th added metal atom that satisfy the criterion Δ*E* ≥ −0.05 eV. Our sampling scheme is designed to efficiently identify robust cluster formation—a necessary precursor to dendrite nucleation—by excluding weakly or non-interacting configurations (*i.e.*, those with Δ*E* ≥ −0.05 eV). By focusing on configurations where the incremental binding energy satisfies Δ*E* ≤ −0.05 eV, we target only those sites likely to yield significant lateral or vertical cluster growth. In the context of battery operation, stable cluster nucleation and growth are understood to correlate with inhomogeneous deposition and dendrite formation.

To evaluate nucleation behavior, interaction energies were calculated using three key metrics: the formation energy *E*_f_, using Zhang and Northrup's standard formalism^87^ (eqn (1)), the binding energy *E*_bind_ (eqn (2)), and the cohesive energy *E*_coh_ (eqn (3)):1
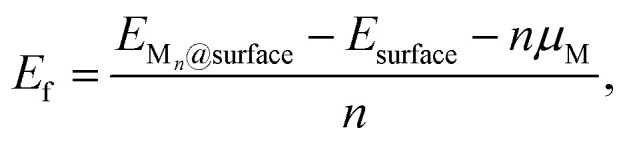
2
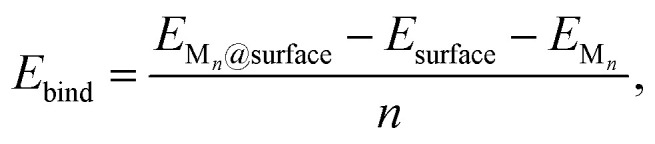
3
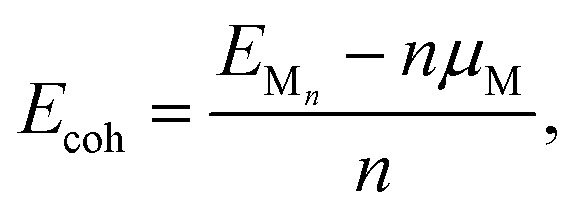
where *E*_M_*n*_@surface_ is the total energy of the M_*n*_ cluster adsorbed on the graphene surface, *E*_surface_ is the energy of the reference surface—either pristine or containing a single carbon vacancy (V_C_)—and *n* is the number of metal atoms in the cluster. The chemical potential *μ*_M_ is taken as the energy of an isolated neutral atom in vacuum; *E*_f_ and *E*_coh_ calculated with *μ*_M_ from bulk metal values are reported in the ESI Fig. S2 and S21† for reference.

The formation energy *E*_f_ quantifies the average energetic cost of assembling the adsorbed cluster from isolated atoms. The binding energy *E*_bind_ captures the net interaction between the cluster and the surface, where negative values indicate attractive interactions and positive values imply repulsion. The cohesive energy *E*_coh_ describes the intrinsic metal–metal bonding within the cluster in vacuum; more negative values correspond to stronger cohesion. These metrics are interdependent. In particular, strong metal–surface interactions (large negative *E*_bind_) often weaken intra-cluster cohesion (less negative *E*_coh_) due to geometric or electronic frustration. This antagonistic relationship provides insight into whether cluster formation is favored over dispersion.

Thermodynamic stability was further assessed using the incremental formation energy relative to the M_1_ representing a disperse coverage with no M–M interactions:

4 Δ*E*_f_ = *E*_f_(*n*) − *E*_f_(*n* = 1).

Clusters with Δ*E*_f_ < 0 are energetically favored to form and grow, whereas Δ*E*_f_ > 0 implies instability and the clustering is unfavoured with respect to the disperse coverage. Note that kinetic effects are not considered in this analysis. For consistency with prior studies, free-energy corrections are omitted here, although several works^62,88–90^ have extended energy-based analyses to incorporate entropic and zero-point contributions. All structural visualizations were prepared using VESTA.^91^

We employ two complementary approaches to elucidate the evolution of the charge transfer. First, we analyze the local coordination environment of the metal atoms by applying directional filtering to classify atoms based on their bonding configurations. This enables us to group metal atoms into distinct categories, such as those with surface coordination or those bonded only to other metal atoms, and to compute the average partial charges within each group as a function of cluster size. Second, we compute the total metal charge *Q*_total_ by summing the Mulliken charges^92^ of all metal atoms in a cluster:5
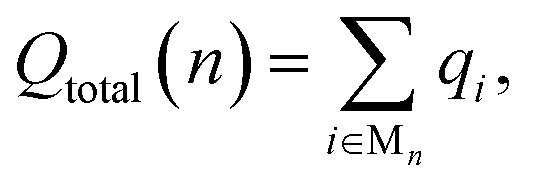
where *q*_*i*_ is the Mulliken charge on the *i*-th metal atom, and *n* is the total number of metal atoms in the cluster. The ideal ionic limit is defined as *nq*_1_, where *q*_1_ is the charge of a single isolated metal atom, extracted from pristine M_1_ systems *via* the oxidation reaction *M*^0^ → *M*^+^. To quantify deviations from full ionic electron transfer, we introduce the fractional ionic character,6
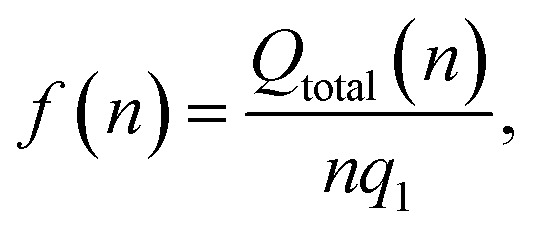
which serves as a descriptor for the balance between electrons transferred to the surface and those retained within the cluster due to metal–metal bonding. By comparing results for pristine and vacancy-modified systems across Li, Na, and K, we provide a comprehensive analysis that correlates the average partial charge behavior with overall electron transfer characteristics as a function of cluster size. These trends are visualized using charge density difference plots to illustrate the spatial redistribution of electrons upon cluster formation. In these plots, the charge density difference Δ*ρ*(**r**) is calculated as7
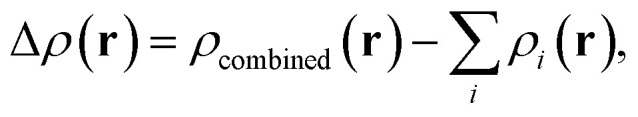
where *ρ*_combined_(**r**) is the total electron density of the combined system (*e.g.*, the M_*n*_ cluster on graphene), and *ρ*_*i*_(**r**) are the electron densities of the isolated components calculated in the same geometry. Regions with Δ*ρ*(**r**) > 0 indicate electron accumulation (electron gain), while regions with Δ*ρ*(**r**) < 0 indicate electron depletion (electron loss).

## 3. Results and discussion

To investigate metal (M) nucleation and clustering on graphene, we systematically examine two systems: (1) the pristine basal plane providing our defect-free reference, and (2) the basal plane containing a carbon vacancy (V_C_). This comparative approach addresses the following key question: How does the presence of a V_C_ defect impact the binding energy (*E*_bind_) and cohesive energy (*E*_coh_) of metal clusters, and consequently, the morphology and stability of the clusters? By analyzing these systems the role of defect-induced surface modifications in promoting or suppressing metal clustering is elucidated.

### 3.1. Metal clustering on the pristine basal plane: trends in stability and morphology

**Fig. 1:**
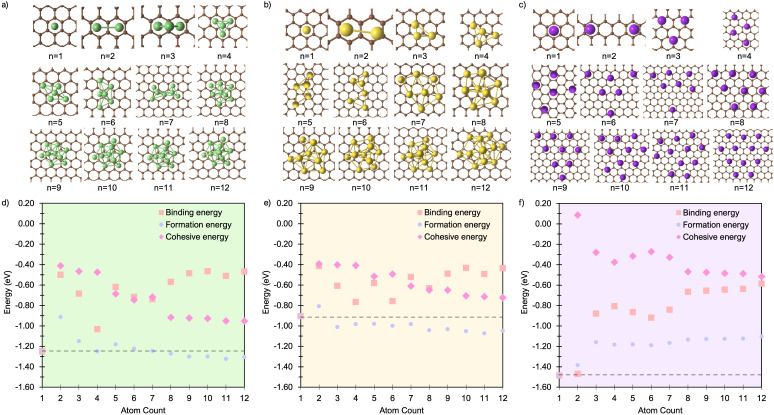
Lowest energy cluster configurations on the pristine basal plane for (a) Li (green spheres represent Li atoms and brown spheres represent C atoms), (b) Na (yellow spheres represent Na atoms), and (c) K (purple spheres represent K atoms). Side views of the lowest energy cluster configurations are included in the ESI (Fig. S3–S5).† The interaction energies decomposed into binding energy (*E*_bind_), cohesive energy (*E*_coh_), and formation energy (*E*_f_) are plotted for Li in (d), Na in (e), and K in (f), with the atom count denoting the number of M atoms in the M_*n*_ clusters.

To investigate the formation of M_*n*_ clusters (*n* = 1–12), we calculated the cluster binding energies on the defect-free graphene basal plane. *E*_bind_, *E*_coh_, and *E*_f_ are shown in Fig. 1, along with the associated cluster geometries. In all cases, the isolated metal atom preferentially adsorbs at the hole site (above the centre of the C_6_ ring), consistent with previous studies.^29,49,93,94^ For clusters with *n* ≥ 2, short-range M–M repulsive interactions distort the geometry, displacing metal atoms from the high-symmetry adsorption site. The degree of distortion is dictated by the inter-site distance between neighbouring hole sites (2.46 Å) in the graphene basal plane and the extent to which M–M separations can be accommodated on the carbon surface.

**Fig. 2:**
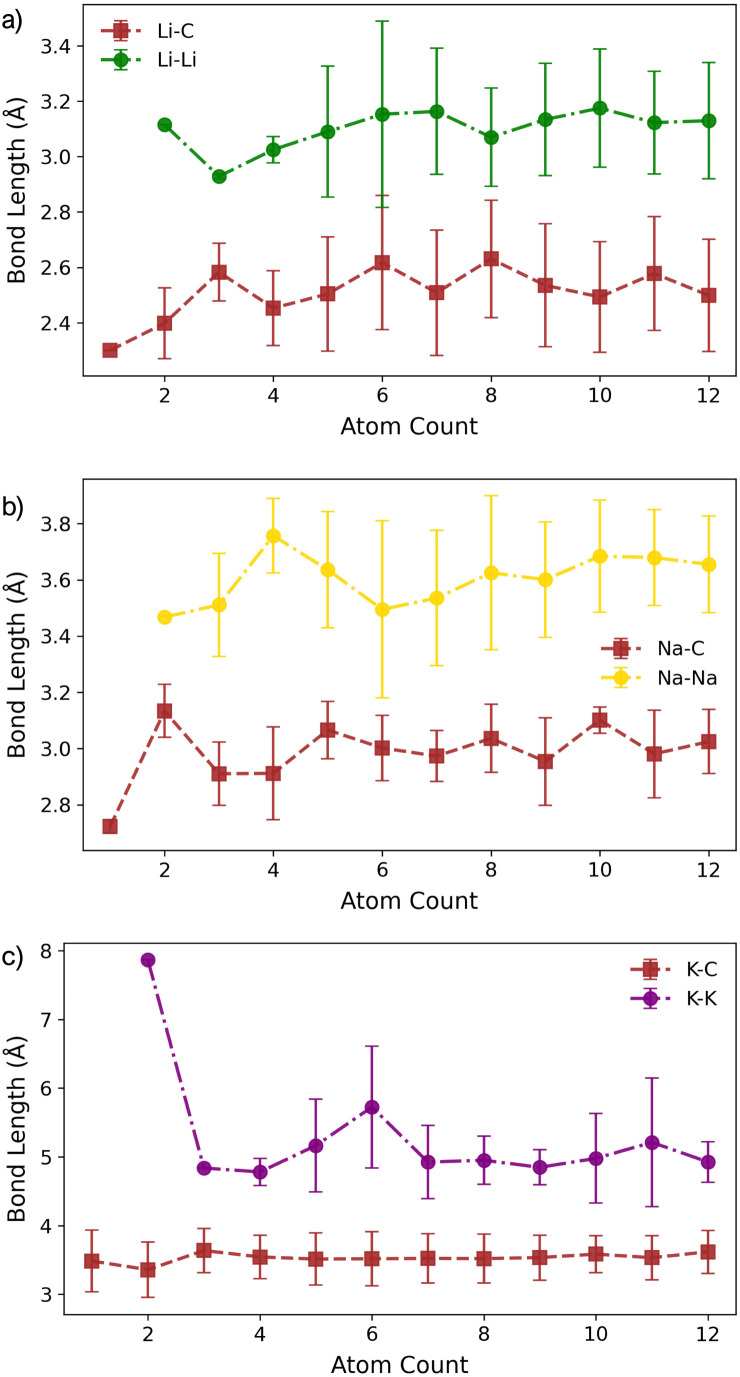
Bond lengths as a function of M cluster size (atom count) for (a) Li, (b) Na, and (c) K clusters on the pristine basal plane, showing the C–M and M–M distances.

For Li_2_ (Fig. 1a and d), the addition of a second Li atom to Li_1_ induces a notable distortion, with a Li–Li separation of 3.12 Å. This displacement from the high-symmetry site (0.33 Å) leads to a number of Li–C bond lengths, replacing the single bond observed in the Li_1_ (Fig. 2a). Na_2_ adopts a similar configuration to Li_2_ but with a greater distortion (3.47 Å) (Fig. 1b), reflecting the larger ionic radius of Na. The increased displacement gives the Na_2_ significant top-site character, driving an increase in the Na–C bond length (Fig. 2b). In contrast, K_2_ shows greater relaxation than either Li or Na, relaxing into a configuration with atoms located at distorted next-next-nearest-neighbour hole sites (Fig. 1c). No stable neighbour or next-neighbour minima were found without applying constraints; upon releasing the constraint, the system relaxed to the next-next-neighbour geometry, corresponding to a K–K separation of 7.86 Å (Fig. 2c).

**Fig. 3:**
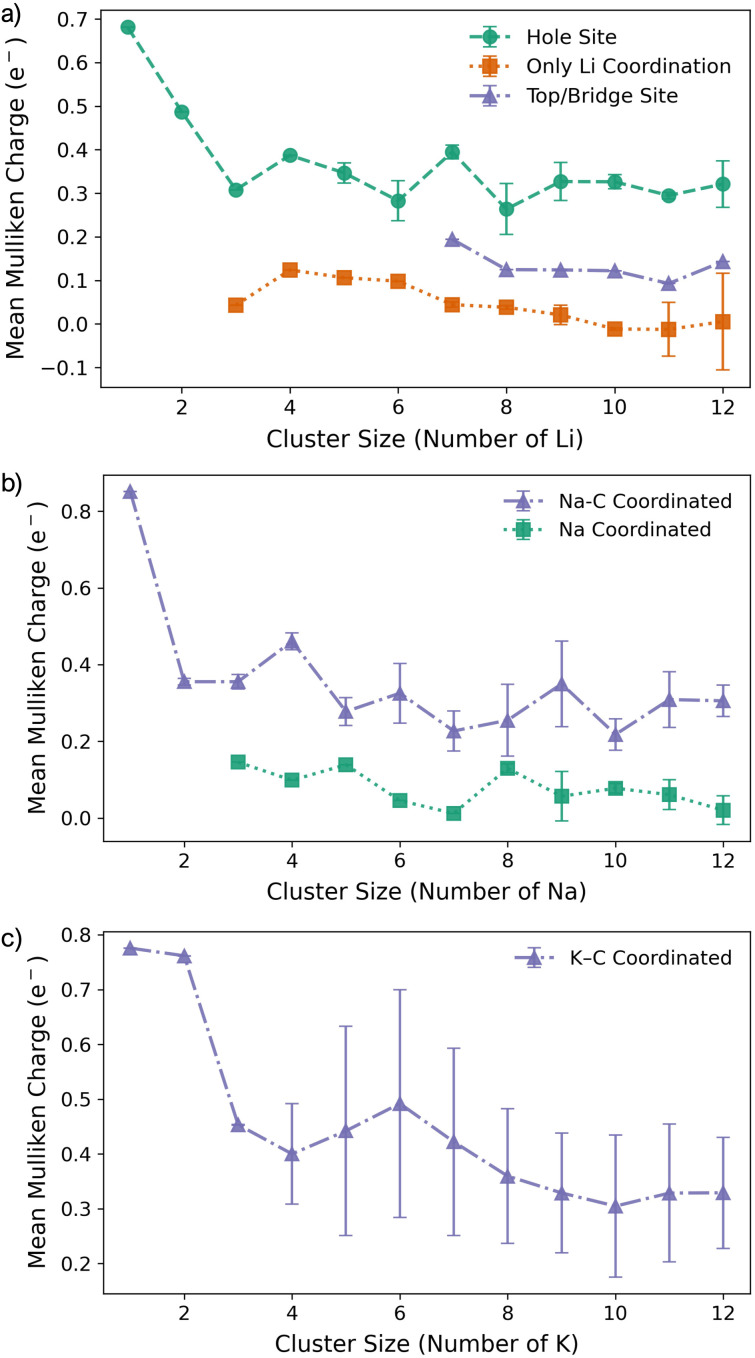
Mean Mulliken charges as a function of M cluster size for (a) Li, (b) Na, and (c) K clusters on the pristine basal plane.

For all Li_1_ and Li_2_ configurations, the dominant interaction is between the metal atom and the surface, as reflected by charge transfer trends (Fig. 3a–c). As previously reported, adsorption of an isolated metal atom results in the transfer of its valence s-electron to the underlying C_6_ ring, following the reaction:M^0^ + C^0^_6_(graphene) → M^+^ + C_6_^−^(graphene),which is captured by Mulliken charges of 0.7–0.85*e*^−^ on the metal and visualised in the Δ*ρ*(**r**) plots (Fig. S12–S14 in the ESI†).^49^ This picture remains valid for K_2_, but for Li_2_ and Na_2_, the presence of a second metal atom leads to a significant reduction in the net positive charge, with Mulliken charges dropping to 0.5*e*^−^ and 0.4*e*^−^, respectively (Fig. 3).

The reduction in partial charge arises from the emergence of partially occupied metal states at the Fermi energy, as shown in the projected density of states (PDOS) (Fig. S11 in the ESI†), and from charge redistribution between the metal centres, evident in the Δ*ρ*(**r**) plots (Fig. S12–S14 in the ESI†). Energetically, M_2_ formation is unfavoured in all cases, with Δ*E*_f_ of +0.34 eV per atom for Li_2_, +0.05 eV per atom for Na_2_, and no stable configuration identified for K_2_; even the relaxed next-next-neighbour geometry remains +0.15 eV per atom higher in energy than the K_1_. While *E*_coh_ for both Li_2_ and Na_2_ is moderately stabilising at −0.40 eV per atom, it is insufficient to counteract the ∼50% reduction in *E*_bind_ relative to the M_1_ reference (Fig. 1d and e).

For K_3_, the trends described previously continue, with two important differences (Fig. 1f). Firstly, *E*_coh_ becomes negative (−0.2 eV per atom), indicating the onset of an attractive K–K interaction. The K–K separation is reduced to 4.8 Å, with next-neighbour top sites occupied (Fig. 2c). Secondly, as observed for Li_2_ and Na_2_, the attractive interaction is driven by the emergence of K-derived states at the Fermi energy (Fig. S11 in the ESI†), which in turn leads to a notable reduction in partial charge (Fig. 3c and S14 in the ESI†).

In contrast, the behaviour of Li_3_ and Na_3_ differs markedly. In both cases, the M_2_ configuration is preserved, with the additional metal atom binding preferentially to the existing metal ions, without direct interaction with the carbon surface (Fig. 1a and b). The influence of M–M interactions is pronounced. Firstly, there is a significant decrease in the M–M bond lengths (Fig. 2b and c), due to the absence of meaningful geometric constraints from the surface lattice. Secondly, *E*_coh_ becomes substantially more favourable for both Li and Na (Fig. 1d and e), while *E*_bind_ remains comparable to that of the dimer, indicating that the energetic benefit arises primarily from the metal–metal interaction.

Finally, analysis of the partial charges reveals the presence of two distinct coordination environments: cationic, surface-adsorbed metal ions (as observed for M_2_), and metal-coordinated sites with no direct C-interaction. The trend established with Li_2_ continues, with a further reduction in the partial charge to 0.33*e*^−^ for surface-bound sites, accompanied by an increased density of Li states at the Fermi level. For Na_3_, the partial charges and associated electronic states remain essentially unchanged relative to Na_2_, indicating that no additional charge redistribution occurs. It is noteworthy that the total reduction in partial charge from Li_1_ to Li_3_ mirrors that observed between Na_1_ and Na_2_, *via* the intermediate Li_2_ configuration. For both Li and Na, the metal-coordinated sites are electronically distinct, exhibiting near-neutral character: approximately 0*e*^−^ for Li and 0.2*e*^−^ for Na (Fig. 3, S12 and S13 in the ESI†). Despite the onset of attractive interactions, cluster formation at *n* = 3 remains strongly disfavoured for Li and K, with Δ*E*_f_ values of +0.20 eV per atom and +0.22 eV per atom, respectively. In contrast, Na cluster formation becomes energetically favourable at this size, with Δ*E*_f_ = −0.11 eV per atom.

The formation of M_4_ clusters marks the point at which the clustering behaviour begins to diverge significantly between the metals. For Li, Li_4_ forms *via* lateral addition to an adjacent hole site, accompanied by relaxation of the previously Li-coordinated atom into the central threefold symmetry site. This structure serves as the fundamental building block for larger clusters, with subsequent growth proceeding by lateral addition to distorted hole, top, or bridge sites (depending on size), followed by vertical growth through the Li-coordinated site. This stepwise occupation is illustrated in Fig. S3 in the ESI.† The mean Li–Li and Li–C bond lengths, shown in Fig. 2a, exhibit a gradual increase in Li–Li separation with cluster size, reaching a maximum spread at Li_6_, where partial formation of a second layer occurs (see Fig. S6 and S7 in the ESI†). The introduction of the threefold-coordinated Li site has a notable effect on the electronic structure, introducing a state deep in the valence band and shifting the charge character towards a more neutral Li^0^ configuration, as evident in Fig. 3 and visualised in the Δ*ρ*(**r**) plots (Fig. S12 in the ESI†).

As cluster size increases, the variation across sites of a given type also increases, reducing the precision with which associated geometric and charge-based descriptors can be defined. *E*_coh_ becomes markedly more favourable with increasing cluster size, although this is partially offset by a reduction in *E*_bind_. Consequently, Δ*E*_f_ becomes increasingly favourable for cluster sizes *n* > 7, with the clustered and dispersed forms becoming energetically degenerate at *n* = 4 and *n* = 7. For lithium, this suggests that clustering can occur under high deposition rates, potentially contributing to the observed plating, with the nucleation probability distributed equally across a degenerate set of surface sites.^52^

Na follows a similar clustering pattern to Li, but with two important differences. Firstly, its larger ionic radius leads to a greater mismatch between the Na–Na bond length and the spacing between surface adsorption sites, resulting in a significantly larger distortion compared to Li (Fig. 2, S8 and S9 in the ESI†). Secondly, *E*_bind_ and *E*_coh_ exhibit substantial overlap, producing a fine balance between metal–metal and metal–carbon interactions (Fig. 1e).

While the general trends mirror those observed for Li, Na clusters consistently exhibit a marked degree of structural distortion at all sizes, a behaviour only seen in Li for the largest clusters (*n* > 10). This structural variability affects the electronic structure: instead of discrete states, the PDOS (Fig. S11 in the ESI†) appears smeared, reflecting the range of geometries present within each cluster. This in turn manifests in the partial charges, where the Na–C interactions span a wide range even in the smallest clusters. Unlike Li, these charges cannot be cleanly distinguished by adsorption site due to the relatively weaker binding of Na (Fig. 3b).

As previously noted, *E*_coh_ and *E*_bind_ lie within a narrow window of approximately 0.4 eV, with *E*_coh_ becoming gradually more favourable and *E*_bind_ increasingly less so as cluster size increases. The resulting formation energy, Δ*E*_f_, indicates that cluster formation becomes energetically favourable for *n* > 2, with Na_2_ already exhibiting a slightly positive Δ*E*_f_ of +0.05 eV per atom. This suggests that Na dendrite formation may occur even on pristine basal plane structures, consistent with experimental observations.^95–98^ However, given the relatively weak binding energy of these Na clusters, further investigation is needed to determine whether they remain anchored to the surface or desorb as isolated entities.

K continues to form a single-layer structure, with atoms spaced to balance metal–metal repulsion against emerging lateral interactions. As the number of K atoms increases, there is a corresponding increase in the spread of K–K distances and the diversity of adsorption sites occupied (Fig. 2c). This variation in site occupancy and degree of lateral interaction leads to a range of partial charges, although the spread remains narrower than that observed for Li and Na, where vertical cluster growth occurs (Fig. 1c, S5 and S10 in the ESI†). Electronically, the behaviour remains consistent with that of K_3_, showing an increase in the density of K-derived states as the cluster size increases. These states remain confined to the Fermi level and above, indicating no significant occupation of lower-energy states (Fig. S11 in the ESI†). Finally, although *E*_coh_ increases modestly with cluster size, this is more than offset by a corresponding decrease in the binding energy (*E*_bind_). As a result, Δ*E*_f_ remains positive across all cluster sizes considered (Fig. 1f). This persistent absence of favourable K clustering supports the conclusion that K nucleation and dendrite formation are energetically unfavourable on pristine basal plane surfaces, consistent with experimental observations in carbon-based KIBs.^97,99,100^

In summary, the formation and stability of metal clusters on the pristine basal plane are governed by a delicate interplay between geometric accommodation, electronic delocalisation, and charge transfer. Li, Na, and K exhibit qualitatively distinct behaviours driven by their ionic radii and their ability to match the spatial periodicity of adsorption sites. Li forms compact clusters with clear layer-by-layer growth beyond *n* = 3, enabled by moderate geometric distortion and strong M–C binding. This growth leads to partial charge redistribution and the emergence of low-lying valence states, although cluster formation remains energetically unfavourable until *n* ≥ 7. Na follows a similar motif, but its larger ionic size imposes greater geometric frustration, resulting in persistent distortion, smeared electronic states, and a complex distribution of partial charges. Despite these distortions, Na cluster formation becomes energetically favourable from *n* = 3, indicating a thermodynamic driving force for nucleation and potential dendrite formation, even in the absence of defects. In contrast, K displays fundamentally different behaviour: the weak M–C binding, strong M–M repulsion, and poor geometric compatibility inhibit both vertical growth and electron localisation. K remains in a dispersed monolayer configuration with minimal electronic perturbation and positive formation energies across all sizes, consistent with the absence of dendrite formation observed experimentally.^97,99,100^

Together, these results highlight how subtle differences in atomic size and metal–surface coupling give rise to distinct clustering pathways, offering mechanistic insight into the conditions under which metal dendrites may nucleate or be suppressed on carbon-based substrates.

### 3.2. Influence of carbon vacancies on metal clustering and stability

**Fig. 4:**
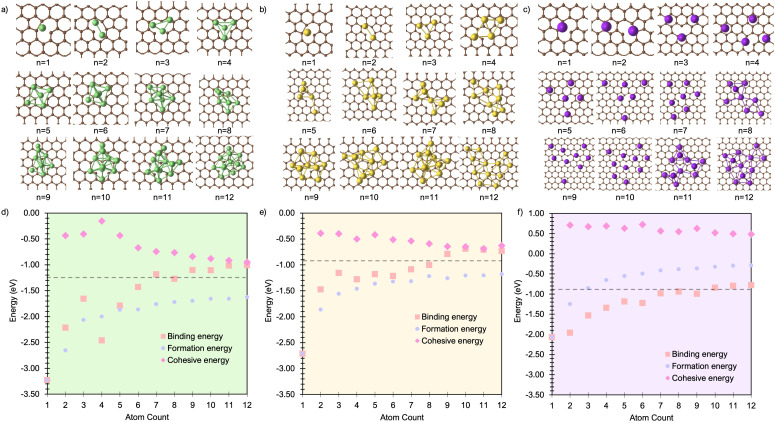
Lowest energy cluster configurations on the defective basal plane for (a) Li (green spheres represent Li atoms and brown spheres represent C atoms), (b) Na (yellow spheres represent Na atoms), and (c) K (purple spheres represent K atoms). Side views of the lowest energy cluster configurations are included in the ESI (Fig. S16–S18).† The interaction energies decomposed into binding energy (*E*_bind_), cohesive energy (*E*_coh_), and formation energy (*E*_f_) are plotted for Li in (d), Na in (e), and K in (f), with the atom count denoting the number of M atoms in the M_*n*_ clusters.

Building on the insights from pristine graphene, we now examine the influence of a single carbon vacancy (V_C_) on metal cluster formation and stability. Fig. 4a–c presents the lowest-energy M_*n*_ cluster geometries on the V_C_ surface for Li, Na, and K (for side views, see Fig. S16–S18 in the ESI†). In all three cases, M_1_ binds preferentially at the vacancy, coordinating to the under-coordinated carbon atom, while the remaining two carbons reconstruct to form a five-membered ring, consistent with earlier work on isolated metal adsorption.^49^ The binding energies (Fig. 4d–f) at *n* = 1 are notably enhanced compared to pristine graphene (Fig. 1d–f), with *E*_bind_ values of −3.23 eV for Li, −2.52 eV for Na, and −2.84 eV for K. This enhancement reflects the strong interaction with the unsaturated carbon atom (Fig. 4a–c).

**Fig. 5:**
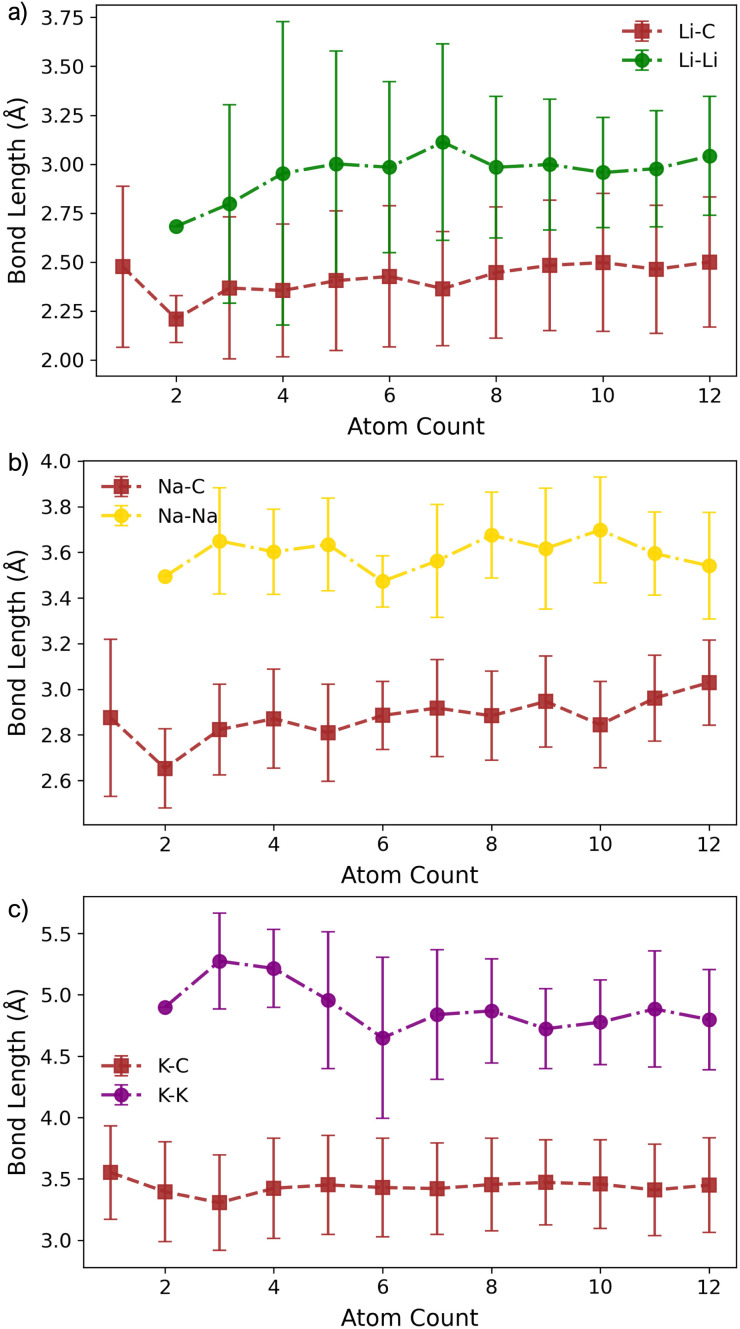
Bond lengths as a function of M cluster size (atom count) for (a) Li, (b) Na, and (c) K clusters on the defective basal plane, showing the C–M and M–M distances.

**Fig. 6:**
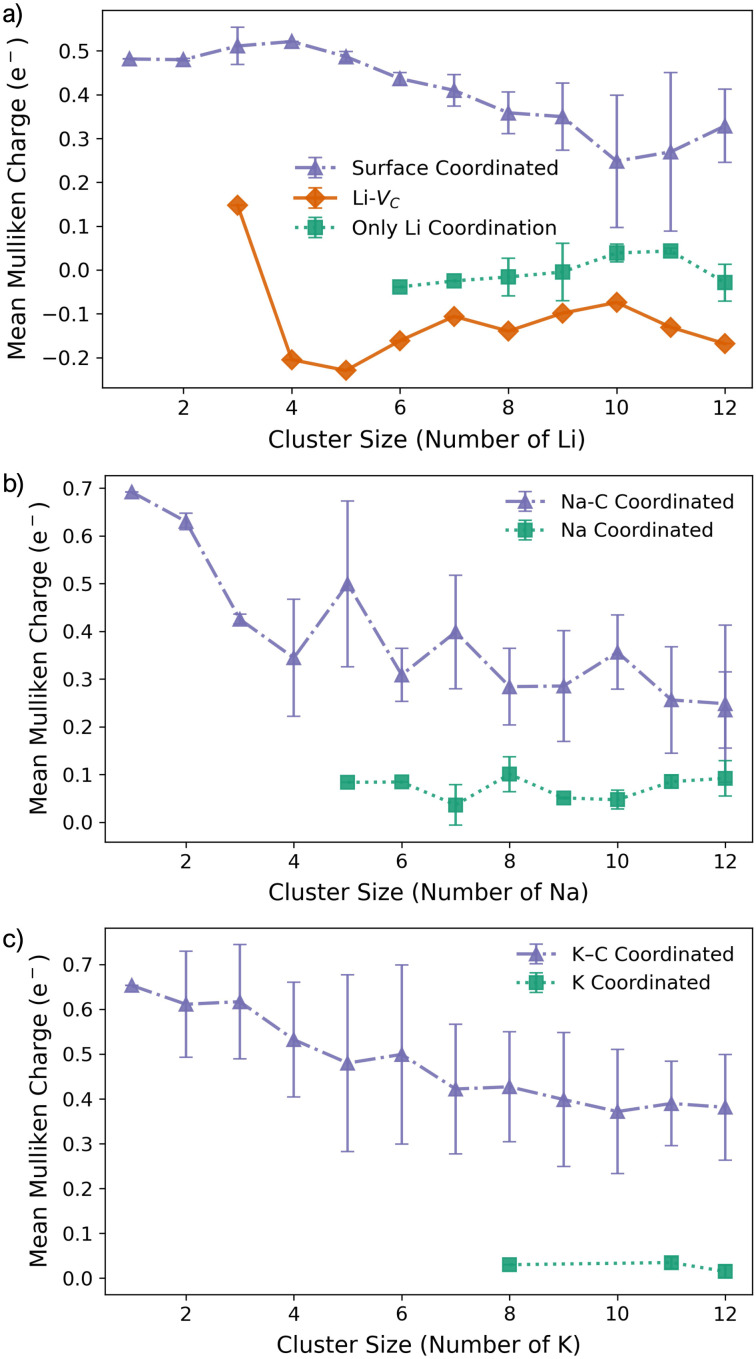
Mean Mulliken charges as a function of M cluster size for (a) Li, (b) Na, and (c) K clusters on the defective basal plane. Δ*ρ*(**r**) plots are presented in Fig. S26–S28 in the ESI.†

The corresponding M–C bond lengths, shown in Fig. 5, are significantly shorter than those in the pristine system (Fig. 2). This shift is driven by the formation of a strong M–C* bond, with distances of 2.03 Å, 2.36 Å, and 2.81 Å for Li_1_, Na_1_, and K_1_, respectively. The presence of the dangling bond alters the charge transfer mechanism: the transferred charge is now predominantly localised on the under-coordinated carbon rather than delocalised over the C_6_ ring (see Fig. S26–S28 in the ESI†). The reactionC^0^_1_ + M^0^ → C_1_^−^ + M^+^leads to a reduced metal partial charge in all cases (most dramatically for Li) indicating an increasingly covalent character to the metal–surface interaction (Fig. 6).

For M_2_, the added atom occupies the adjacent vacancy hole site in the case of Li_2_ and Na_2_, and the next-neighbour hole site for K_2_, with both metal centres interacting with the unsaturated carbon atom. The Li and Na atoms occupy equivalent sites and exhibit negligible extension relative to M_1_ (M–C* distances: Li_2_, 2.05 Å; Na_2_, 2.36 Å) (Fig. 5a and b). In contrast, K_2_ adopts a more asymmetric configuration, with one atom at the vacancy and the other on the next-neighbour hole site, giving K–C* distances of 2.81 Å and 3.68 Å, respectively (Fig. 5c). The partial charges (Fig. 6) remain largely consistent for Li_2_, while Na_2_ and K_2_ exhibit a small decrease of approximately 0.1*e*^−^. In all cases, the charge transfer extends beyond the under-coordinated carbon to neighbouring C atoms, as shown in Fig. S26–S28 in the ESI.† Inspection of the PDOS (Fig. S25 in the ESI†) highlights a key distinction from the pristine system: the metal-derived states remain unoccupied and lie well above the Fermi level, whereas in the pristine case, these states are partially filled. *E*_f_ is dominated by the strong binding of M_2_ to the vacancy site (*E*_bind_), although it is reduced compared to the M_1_ case. *E*_coh_ is marginally more favourable than on the pristine surface, but its impact on Δ*E*_f_ is negligible (Fig. 4). All M_2_ configurations remain strongly stabilised, with Δ*E*_f_ values of −1.2 eV per atom, −1.0 eV per atom, and −0.75 eV per atom for Li_2_, Na_2_, and K_2_, respectively.

At M_3_, Li exhibits markedly different behaviour. At Li_3_, the five-membered carbon ring begins to open, with the C–C bond extending beyond 1.9 Å. This structural change is reflected in the partial charges (Fig. 6), where the Li atom interacting with the breaking C–C bond exhibits a significantly reduced Mulliken charge. At Li_4_, the ring fully ruptures, enabling one Li atom to coordinate to three equivalent dangling carbon atoms with a short C–Li bond length of 1.85 Å. These under-coordinated carbon atoms are displaced upwards by approximately 1.2 Å from the graphene plane (Fig. 4a). The partial charge on this vacancy-bound Li site drops further −0.1*e*^−^, while the Li atoms occupying nearby hole sites retain a consistent charge of 0.5*e*^−^ (Fig. 6a). Following this reconstruction, additional Li atoms preferentially occupy adjacent hole sites and, beyond Li_5_, begin to adsorb vertically, initiating second-layer growth. This growth pathway mirrors that seen on the pristine surface, with the vacancy acting as an anchoring centre. The transition observed at Li_3_ and Li_4_ marks a shift from formation energy (*E*_f_) dominated by strong metal–surface binding (*E*_bind_) to one increasingly governed by metal–metal cohesion (*E*_coh_). Beyond Li_4_, *E*_bind_ becomes progressively less favourable with cluster size, while *E*_coh_ continues to increase. Nevertheless, all Li_*n*_ configurations remain energetically stabilised, with Δ*E*_f_ negative across the entire size range considered.

For both Na and K, the five-membered carbon ring (C_5_) remains intact across all cluster sizes (Fig. 4b and c). For Na, clustering follows the same intermediate configurations as observed on the pristine surface, with small clusters (*n* ≤ 3) anchored at the vacancy site, rendering their formation significantly more favourable. The presence of the vacancy leads to an increased spread in bond lengths (Fig. 5b) and partial charges (Fig. 6b), although the mean values remain comparable to those on the pristine surface. This broadened distribution arises from the combination of local distortion induced by the vacancy and the fixed nature of tightly bound atoms in the small clusters, which are energetically costly to displace. Consequently, additional atoms must accommodate these anchored positions, introducing greater structural distortion within the growing cluster, as illustrated schematically in Fig. S22 and S23 in the ESI.† The PDOS shows a similar trend to the pristine case: Na–Na interactions produce states degenerate with the Fermi level, while non-carbon-coordinated Na atoms contribute deeper-lying valence band states (Fig. S25 in the ESI†).

For all Na_*n*_ clusters considered, Δ*E*_f_ remains negative, indicating favourable clustering. As with Li, a gradual transition is observed from an initial regime dominated by strong local vacancy interactions (*E*_bind_) to a regime where *E*_coh_ and *E*_bind_ contributions are of comparable magnitude, approaching the energetic balance seen on the pristine surface.

For K, clustering proceeds in a manner broadly similar to the pristine system, with two notable exceptions. First, there is a marked energetic antagonism between the vacancy-bound K atom and additional K atoms occupying non-vacancy sites during vertical growth. This competition results in favourable multilayer configurations emerging only at K_8_, K_11_, and K_12_, though isoenergetic lateral configurations exist at each of these sizes. Second, the formation energy Δ*E*_f_ is negative only for clusters up to K_5_, where the strong local vacancy interaction dominates. The comparatively weak *E*_coh_ of potassium limits the transition to a surface-anchored growth mode seen for both Li and Na. As a result, while the vacancy can stabilise small K_*n*_ clusters, it does not promote sustained growth. This reinforces the conclusion that potassium remains resistant to nucleation and dendrite formation, even in the presence of surface defects.

### 3.3. Discussion

The introduction of a carbon vacancy (V_C_) dramatically alters the thermodynamics of metal clustering on the graphene basal plane. For all three metals, the presence of an unsaturated carbon at the vacancy enhances the local binding energy (*E*_bind_) of the initial adsorbed atom, leading to stronger anchoring and the stabilisation of otherwise unfavourable small clusters. As cluster size increases, however, this effect becomes localised: the vacancy contribution is diluted on a per-atom basis, and *E*_bind_ converges toward the pristine values. Concurrently, *E*_coh_ grows steadily more favourable, approaching the bulk metal limit. This crossover from vacancy-dominated to cluster-internal energetics provides a natural scale for predicting cluster size thresholds for nucleation and vertical growth.

Geometrically, the vacancy site introduces a strong asymmetry that constrains early-stage cluster morphology. For Li, the vacancy drives a structural transformation: the five-membered ring opens at *n* = 3, enabling a central Li atom to coordinate to three under-coordinated carbon atoms at *n* = 4. This geometry stabilises the cluster and defines the onset of vertical growth. In contrast, Na and K preserve the five-membered ring across all cluster sizes. This rigidity, combined with the larger equilibrium M–M distances, limits the accessible surface adsorption geometries and restricts metal-carbon relaxation. These trends are mirrored in both the metal–carbon bond lengths and partial charge distributions: Li exhibits shorter bonds and enhanced covalent character at the vacancy, while Na and K maintain the single dangling bond interaction.

The evolution of the electronic structure reflects the transition from localised surface binding to collective metallic behaviour. For Li and Na, small clusters exhibit sharp distinctions in the PDOS, with surface-bound atoms contributing states near the Fermi level, while interior or second-layer atoms retain deeper-lying, metal-like character. This evolution is accompanied by a progressive reduction in Mulliken partial charge with increasing cluster size, consistent with charge delocalisation and the emergence of quasi-metallic behaviour. For Li, the partial charge on the vacancy-bound site drops from ∼0.5*e*^−^ at *n* = 1 to ∼0.1*e*^−^ at *n* = 4, and reaches ∼–0.2*e*^−^ at *n* = 5. Na exhibits a more gradual reduction, with the vacancy-bound site stabilising near ∼0.2*e*^−^ by *n* = 6. In both cases, the emergence of metal-derived states degenerate with the Fermi level coincides with lateral metal–metal interaction, while vertical growth introduces deeper-lying valence band states associated with non-surface-bound atoms.

**Fig. 7:**
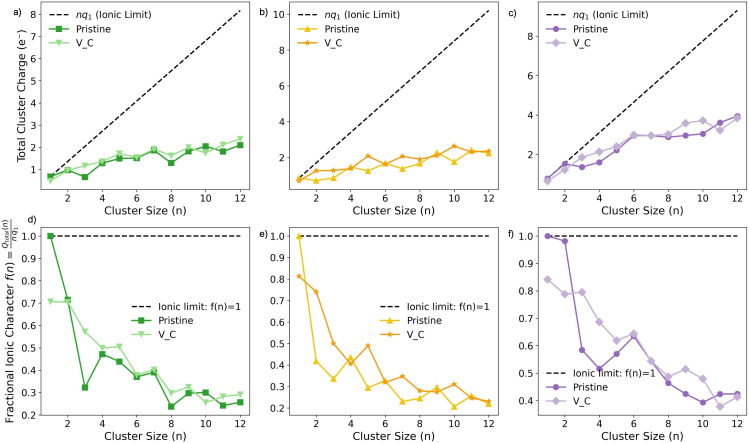
Total metal cluster charge (*Q*_total_) for (a) Li, (b) Na, and (c) K, and their fractional character (*f*(*n*)) for (d) Li, (e) Na, and (f) K.

To further quantify the interplay between electron transfer and cluster growth, we calculate the total cluster charge *Q*_total_ (Fig. 7a–c) and the fractional ionic character *f*(*n*) (Fig. 7d–f). *Q*_total_ represents the summed Mulliken charge of all metal atoms in a given cluster, while *f*(*n*) = *Q*_total_/(*nq*_1_) normalizes this value relative to the ideal ionic limit, defined by the charge *q*_1_ of the isolated surface adsorbed metal (M_1_). For all metals on the pristine surface, *Q*_total_ increases sublinearly with *n*, indicating a progressive departure from predominantly ionic behavior (Fig. 7a–c). This deviation is most pronounced for Li and Na, where *f*(*n*) drops rapidly from unity to approximately 0.3 by *n* = 6–8, consistent with the emergence of strong metal–metal bonding within the cluster (Fig. 7d and e). In contrast, K exhibits weaker charge transfer overall, with *f*(*n*) remaining above 0.4 even at the largest cluster sizes studied (Fig. 7f). The presence of a carbon vacancy primarily influences the smallest clusters (*n* = 1–3), where electron transfer is enhanced through interaction with the vacancy's dangling bond. At *n* = 1, *f*(*n*) increases to 0.71, 0.82, and 0.85 for Li, Na, and K respectively. However, beyond *n* = 4, the influence of the vacancy saturates, and *f*(*n*) converges toward the pristine trend. These trends confirm that electron donation to the surface is rapidly exhausted, and that beyond a critical cluster size, excess charge remains within the cluster itself – consistent with the development of delocalized metallic states near, and eventually below, the Fermi level (Fig. S11 and S25 in the ESI†). Importantly, the vacancy has only a modest effect on *f*(*n*) beyond the smallest sizes, reinforcing its role as a local defect that governs early-stage nucleation without significantly altering the longer-range electronic character of the cluster.

Overall, these findings highlight the dual role of carbon vacancies in mediating early-stage metal nucleation. Vacancies not only stabilise small clusters that are otherwise unfavourable on pristine graphene, but also imprint distinct structural and electronic fingerprints on the growing ensemble. The vacancy acts as an anchor point, localising charge and stabilising low-coordinate geometries, while facilitating a controlled transition to extended, metallic clusters through lateral and vertical addition. The extent to which this anchoring effect persists depends sensitively on the balance between vacancy–metal binding, metal–metal cohesion, and the geometric constraints imposed by ionic radius. As a result, the vacancy-driven nucleation landscape is element-specific: Li benefits from strong, directional bonding and cohesive growth; Na occupies an intermediate regime where distortion accommodates nucleation; and K remains largely non-nucleating, even with defect assistance. These distinctions offer a predictive framework for interpreting alkali metal behaviour on defective carbon and for designing anode materials that control clustering through tailored surface chemistry.

## 4. Conclusions

This study systematically examines the nucleation and growth of Li, Na, and K clusters on both pristine graphene and defective surfaces featuring a single carbon vacancy (V_C_), elucidating the thermodynamic pathways underlying dendrite formation in alkali metal-ion batteries.

On the pristine basal plane, cluster growth is hindered for Li due to strong M–M repulsion and the unfavourable energetics of small clusters. K exhibits minimal clustering across all sizes, reflecting its weak binding and large ionic radius. In contrast, Na clusters readily form, with Δ*E*_f_ becoming favourable as early as *n* = 3, suggesting an inherent propensity for dendrite nucleation even in the absence of defects. The introduction of a vacancy site dramatically alters this picture. For Li, the V_C_ stabilises otherwise unstable small clusters, enabling stepwise growth anchored at the defect. For Na, already favourable clustering is further enhanced by the vacancy, reinforcing inhomogeneous nucleation pathways. K, while weakly bound overall, shows modest cluster stabilisation at small sizes, but fails to achieve favourable vertical growth, maintaining resistance to dendrite formation.

These behaviours arise from the interplay between metal–carbon binding, metal–metal cohesion, and electronic structure evolution, with the vacancy shifting the balance from surface-limited to metal-dominated growth. Importantly, the findings reveal that even a single point defect can serve as a nucleation hotspot, transforming otherwise uniform growth into spatially heterogeneous clustering. Given the diversity of intrinsic and extrinsic defects in real carbonaceous anodes, extending this analysis beyond vacancies will be essential. Such insights will inform strategies for defect-engineered electrode design aimed at suppressing dendrite formation and enhancing long-term electrochemical stability.

## Data availability

Data for this article are available at Zenodo at https://doi.org/10.5281/zenodo.14747737.

## Conflicts of interest

There are no conflicts to declare.

## Supplementary Material

SE-009-D5SE00130G-s001
