# Correction: Vacancy enhanced Li, Na, and K clustering on graphene

**DOI:** 10.1039/d5se90077h

**Published:** 2025-10-08

**Authors:** Jonathon Cottom, Qiong Cai, Emilia Olsson

**Affiliations:** a Advanced Research Center for Nanolithography Science Park 106 Amsterdam 1098 XG The Netherlands; b School of Chemistry and Chemical Engineering, University of Surrey Guildford GU2 7XH UK; c Institute of Theoretical Physics, Institute of Physics, University of Amsterdam Science Park 904 Amsterdam 1098 XH The Netherlands k.i.e.olsson@uva.nl

## Abstract

Correction for “Vacancy enhanced Li, Na, and K clustering on graphene” by Jonathon Cottom *et al.*, *Sustainable Energy Fuels*, 2025, **9**, 2813–2826, https://doi.org/10.1039/D5SE00130G.

The authors regret that Fig. 4f in the original version incorrectly showed the K system energies with reference to the potassium metal potential (as included in the SI). The corrected figure is provided here, showing the energies as referenced to the vacuum chemical potential reference, corresponding to the text in the paper. Despite this change, the authors confirm that the correction of Fig. 4f does not change the conclusions of the paper.

**Fig. 4:**
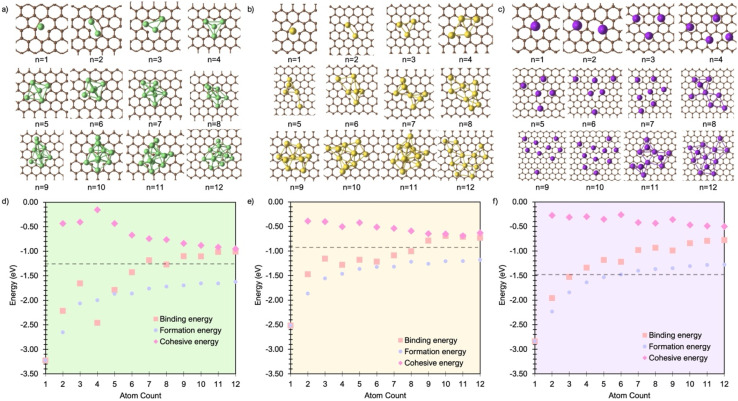
Lowest energy cluster configurations on the defective basal plane for (a) Li (green spheres represent Li atoms and brown spheres represent C atoms), (b) Na (yellow spheres represent Na atoms), and (c) K (purple spheres represent K atoms). Side views of the lowest energy cluster configurations are included in the SI (Fig. S16–S18). The interaction energies decomposed into binding energy (*E*_bind_), cohesive energy (*E*_coh_), and formation energy (*E*_f_) are plotted for Li in (d), Na in (e), and K in (f), with the atom count denoting the number of M atoms in the M_*n*_ clusters.

The Royal Society of Chemistry apologises for these errors and any consequent inconvenience to authors and readers.

