# Supplementary material for: Vacancy enhanced Li, Na, and K clustering on graphene
Source: Sustain Energy Fuels. 2025 Apr 16;9(10):2813–26. doi: 10.1039/d5se00130g (PMC12013468; doi:10.1039/d5se00130g)
Supplement: SE-009-D5SE00130G-s001 [file SE-009-D5SE00130G-s001.pdf]

Supplementary Information (SI) for Sustainable Energy & Fuels.
This journal is © The Royal Society of Chemistry 2025

Supporting Information for "Vacancy Enhanced Li, Na, and K clustering on graphene"

Jonathon Cottom[1,2], Qiong Cai[3], and Emilia Olsson[1,2]

[1]Advanced Research Center for Nanolithography, the Netherlands
[2]Institute of Theoretical Physics, Institute of Physics, University of Amsterdam, the Netherlands
[3]School of Chemistry and Chemical Engineering, University of Surrey, United Kingdom

S1 Graphene supercell models

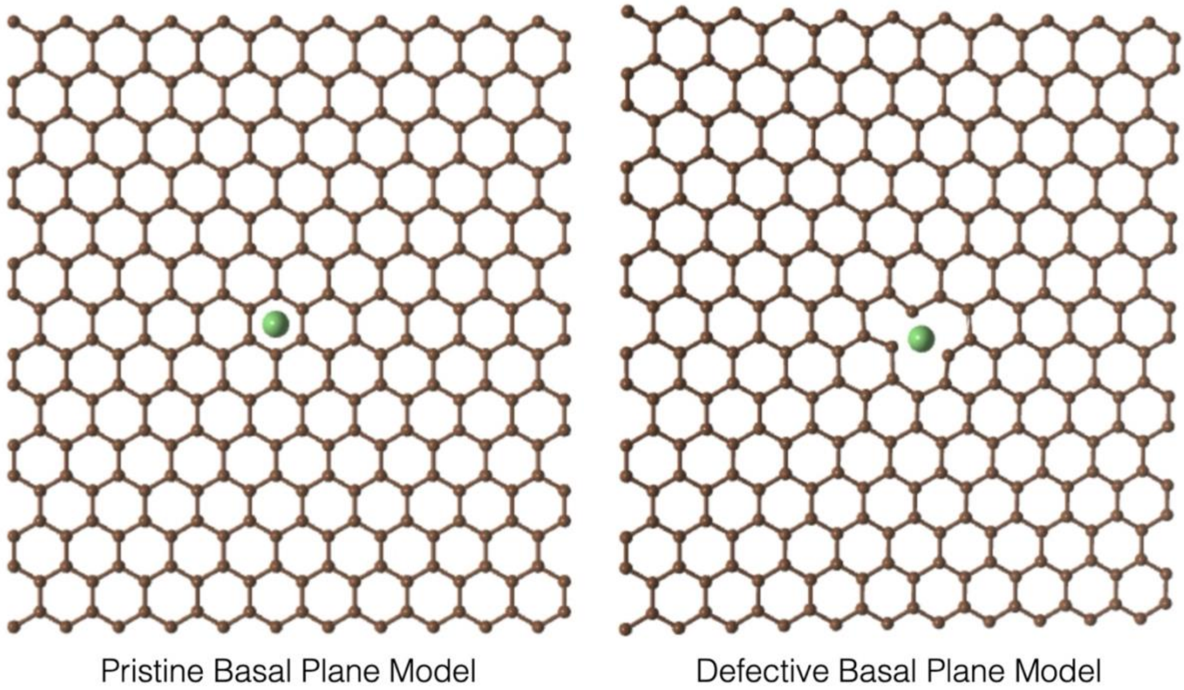

Figure S1. Ball and stick models showing the full simulation cells of one Li on the pristine basal plane model and the defective (with carbon vacancy) basal plane model. The simulation cells are both 27.16 Å× 29.92 Å with a 25 Å vacuum slab. Brown spheres are carbon and green lithium. The Li models are representative of the single Na and K models as well.

S2 M_n on Pristine graphene

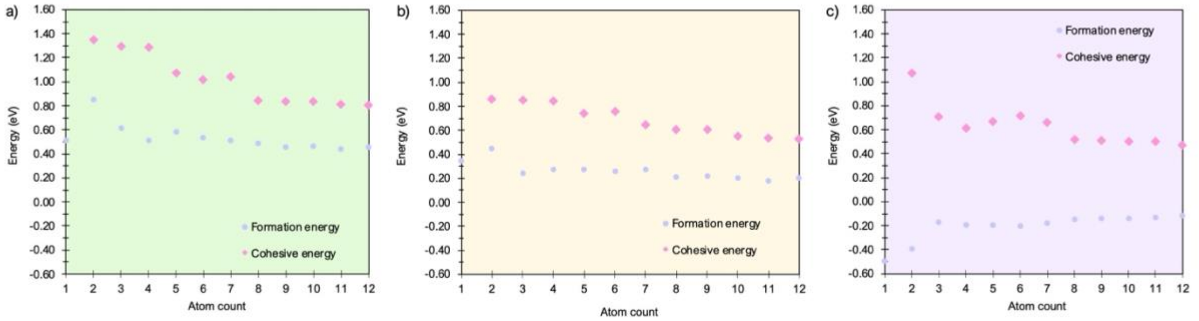

Figure S2. Interaction energies on pristine graphene decomposed into binding energy, cohesive energy, and formation energy with metal chemical potential plotted for Li in (a), Na in (b), and K in (c), with the atom count denoting the number of M atoms in the M_n clusters.

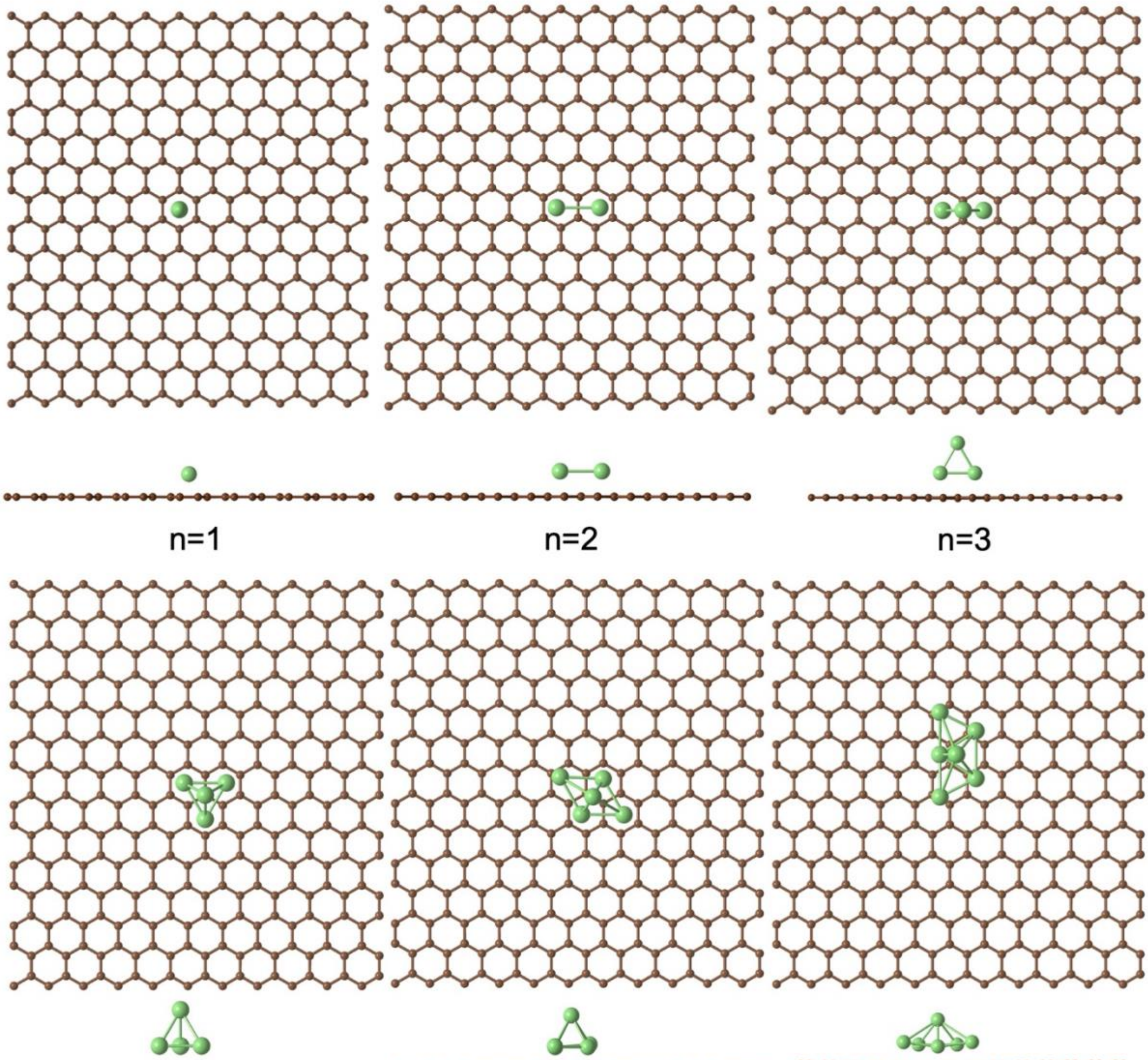
n=1
n=2
n=3
n=4
n=5
n=6

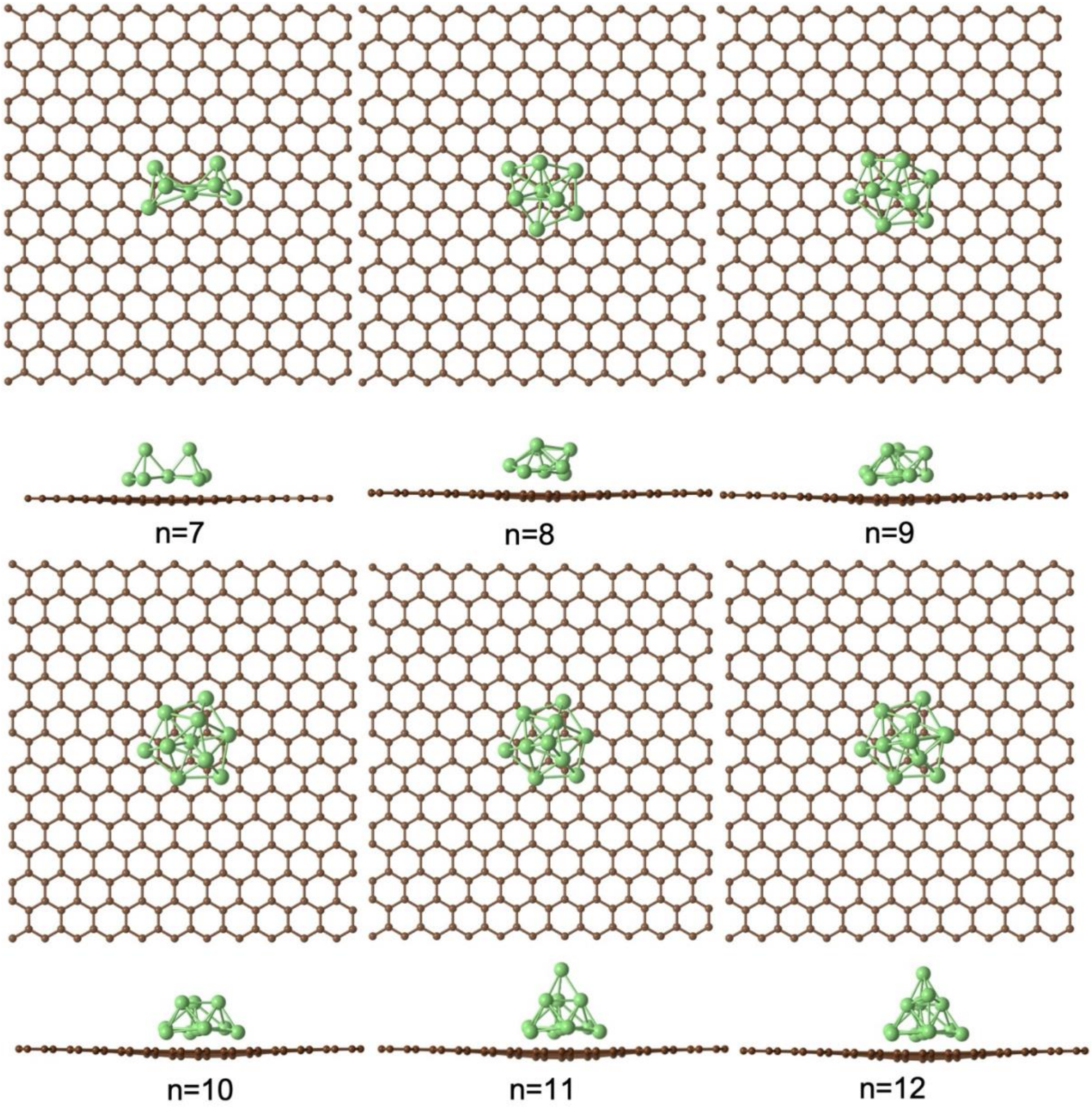

Figure S3. Top and side view of lowest energy Li_n (n=1-12) on pristine basal plane for the data presented in Figure 1a in the main article. Brown spheres are carbon and green lithium.

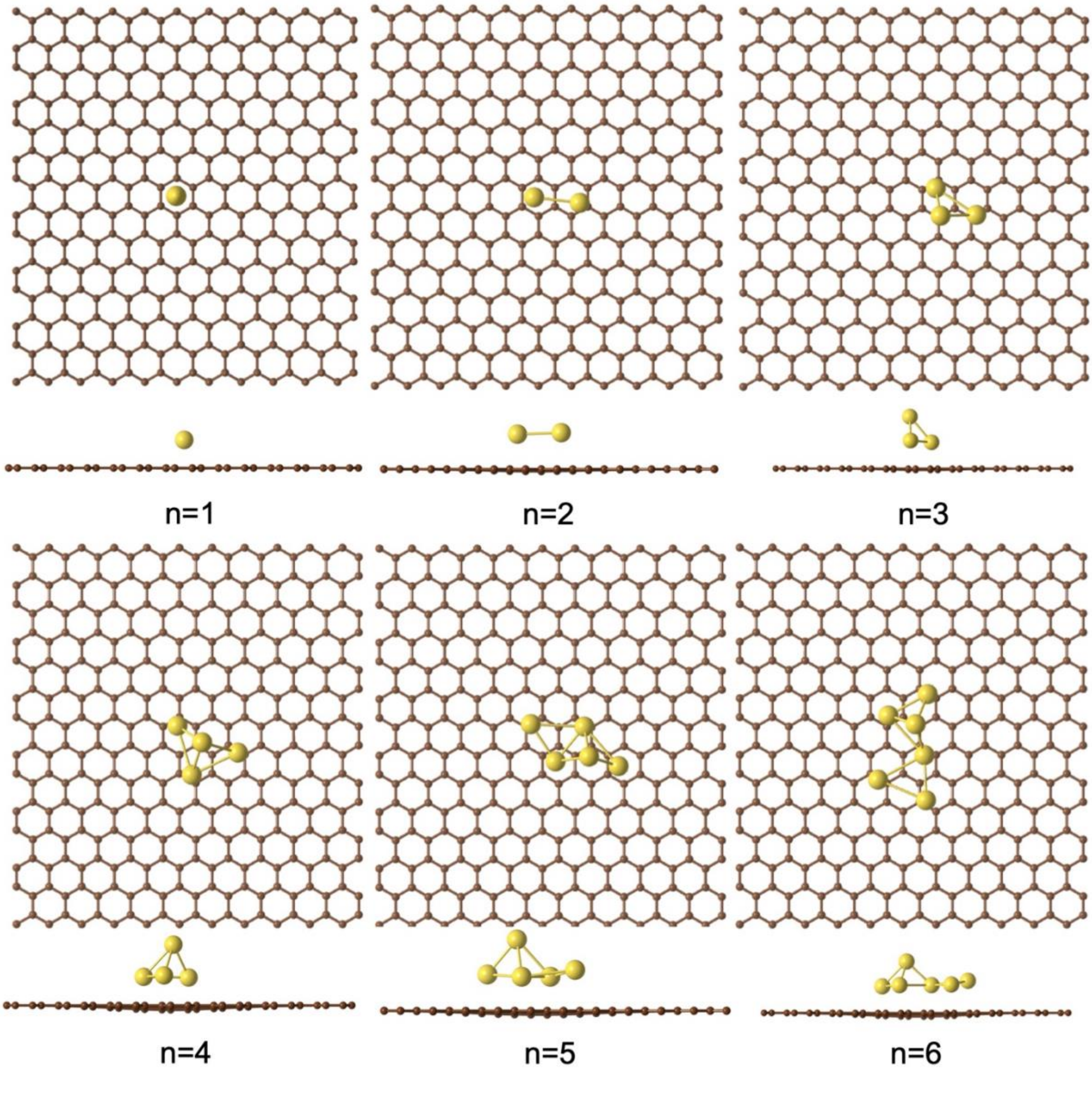
n=1
n=2
n=3
n=4
n=5
n=6

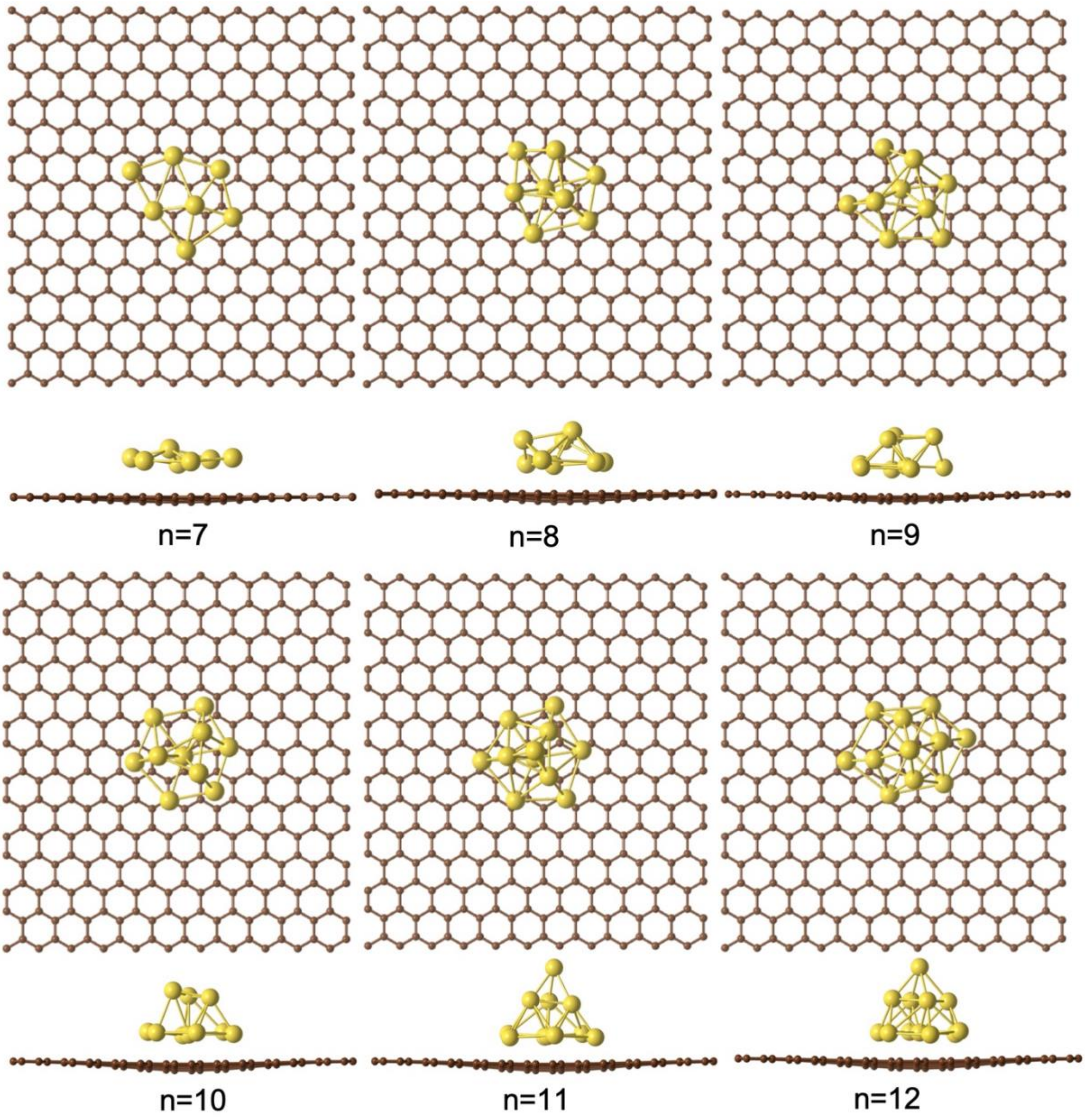

Figure S4. Top and side view of lowest energy Na_n (n=1-12) on pristine basal plane for the data presented in Figure 1b in the main article. Brown spheres are carbon and yellow sodium.

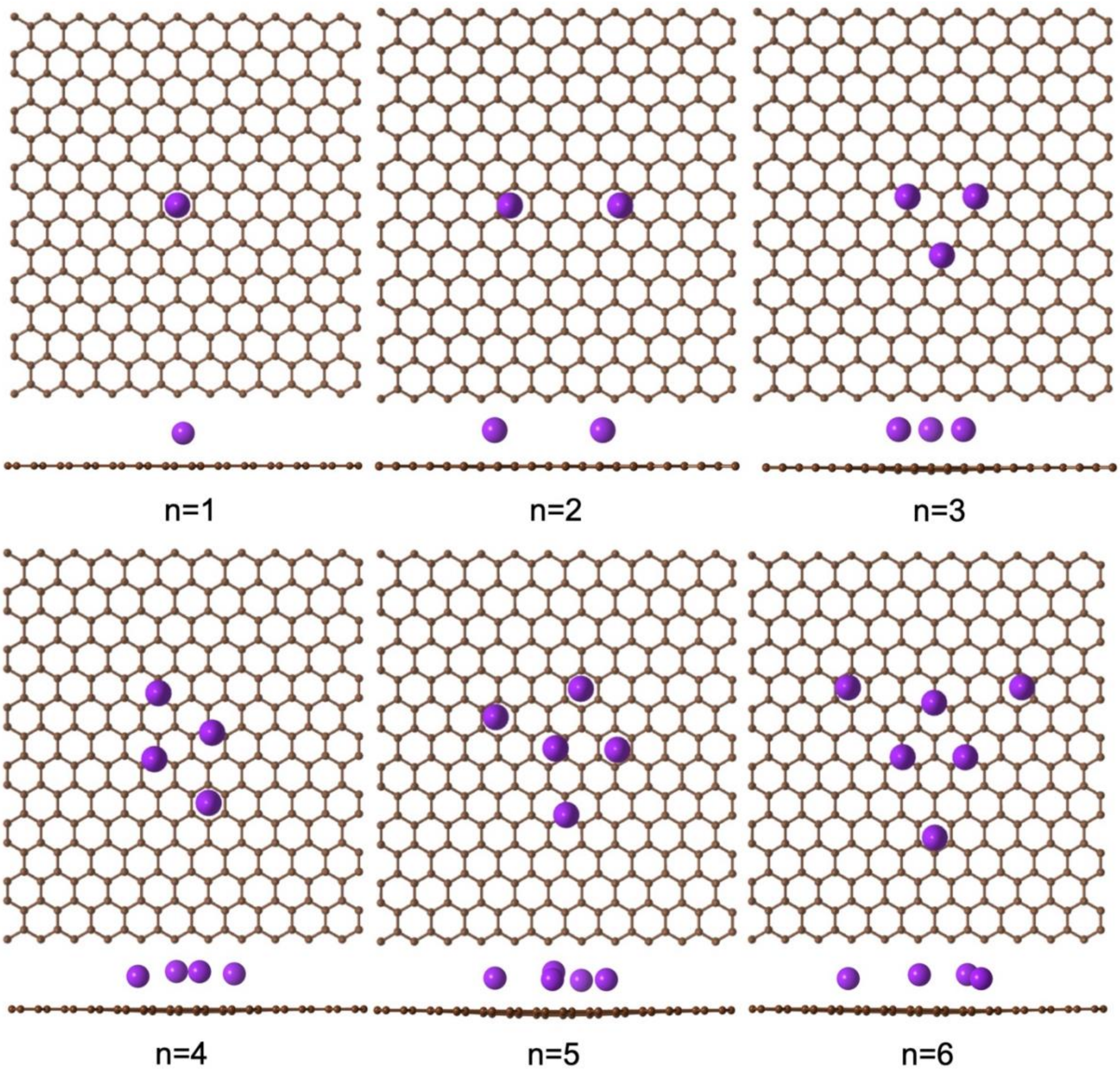

n=1
n=2
n=3
n=4
n=5
n=6

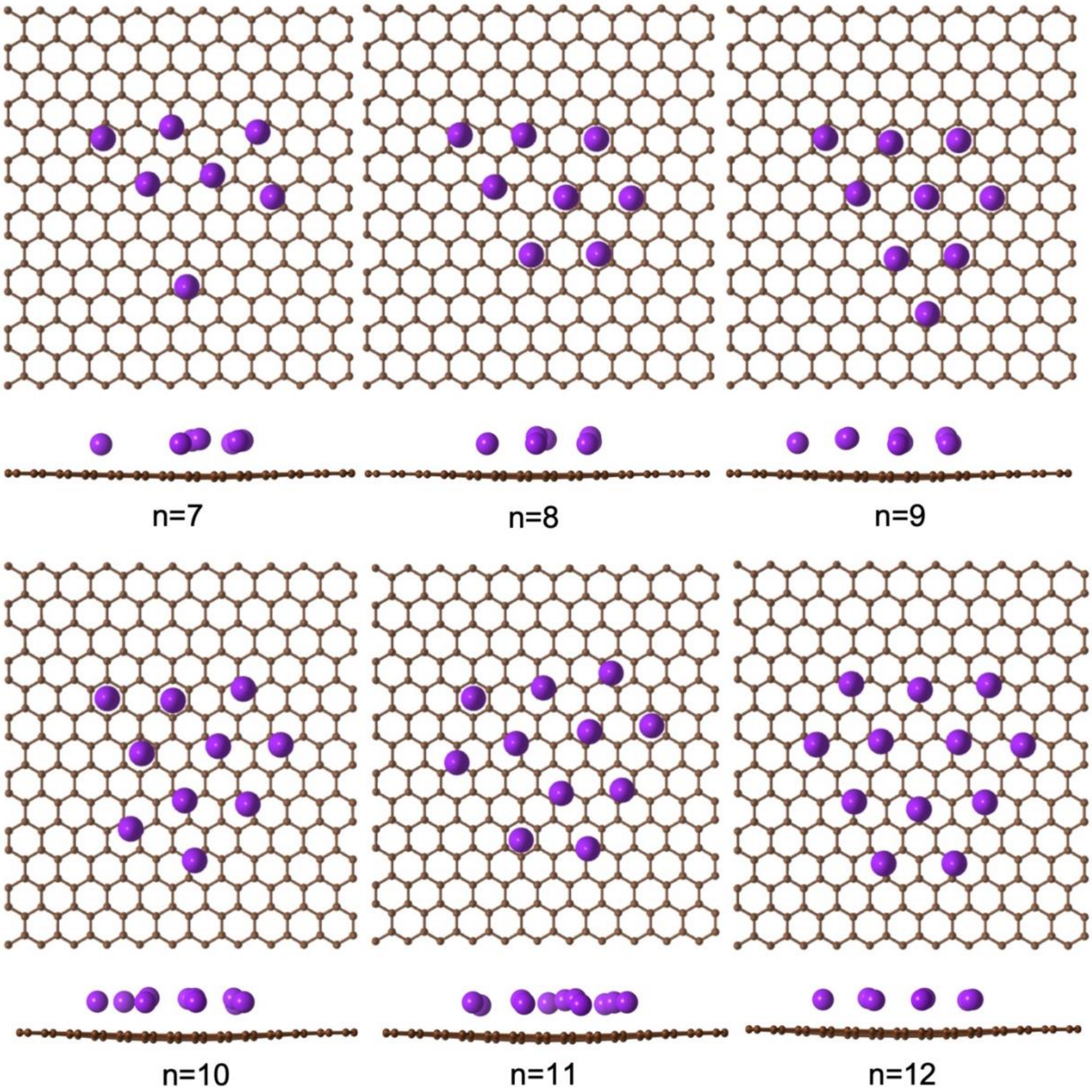

Figure S5. Top and side view of lowest energy K_n (n=1-12) on pristine basal plane for the data presented in Figure 1c in the main article. Brown spheres are carbon and purple potassium.

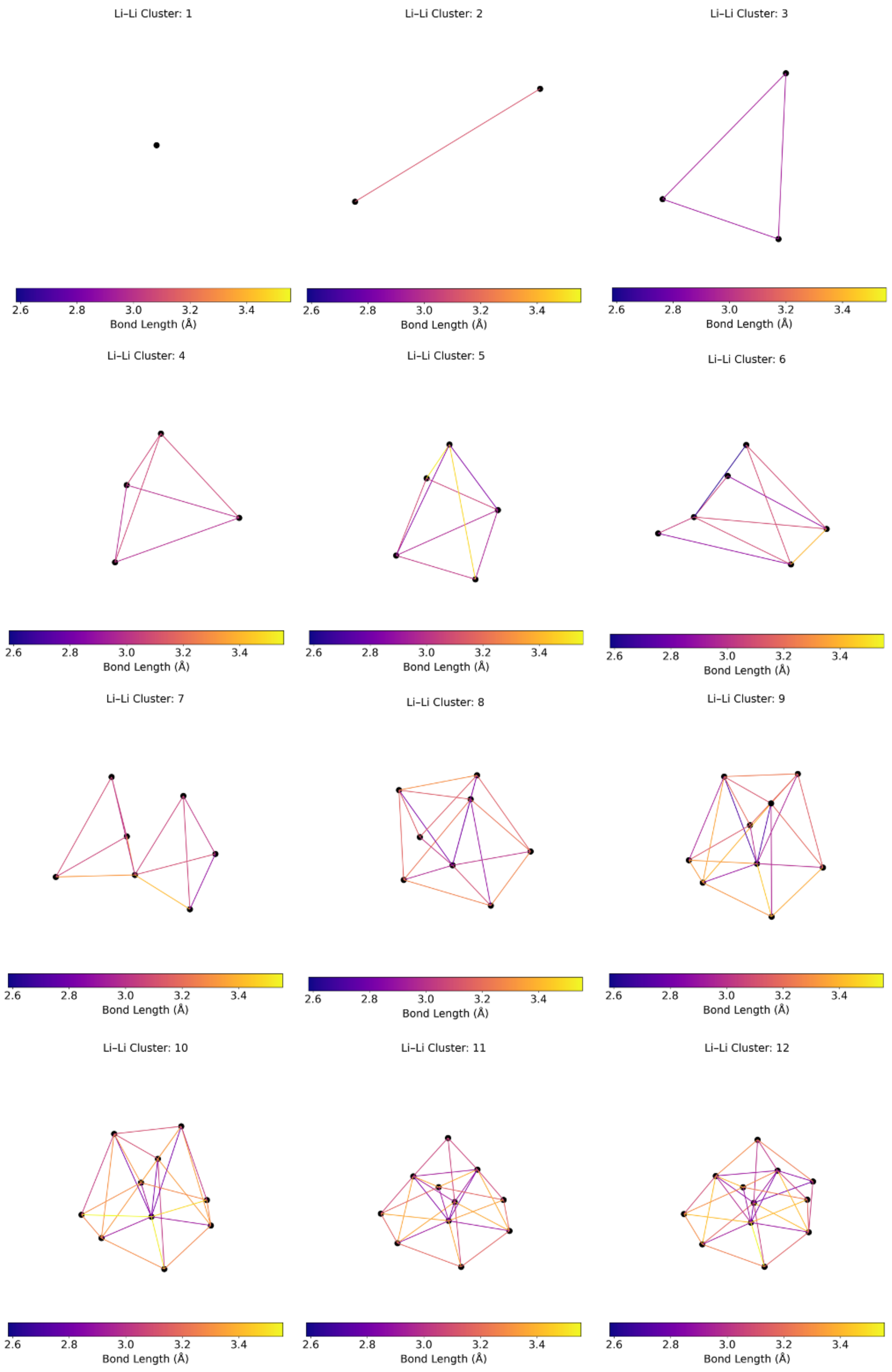

Figure S6. Li-Li distances in Li_n clusters on pristine graphene. The graphene Li_n distances are included in Figure S7.

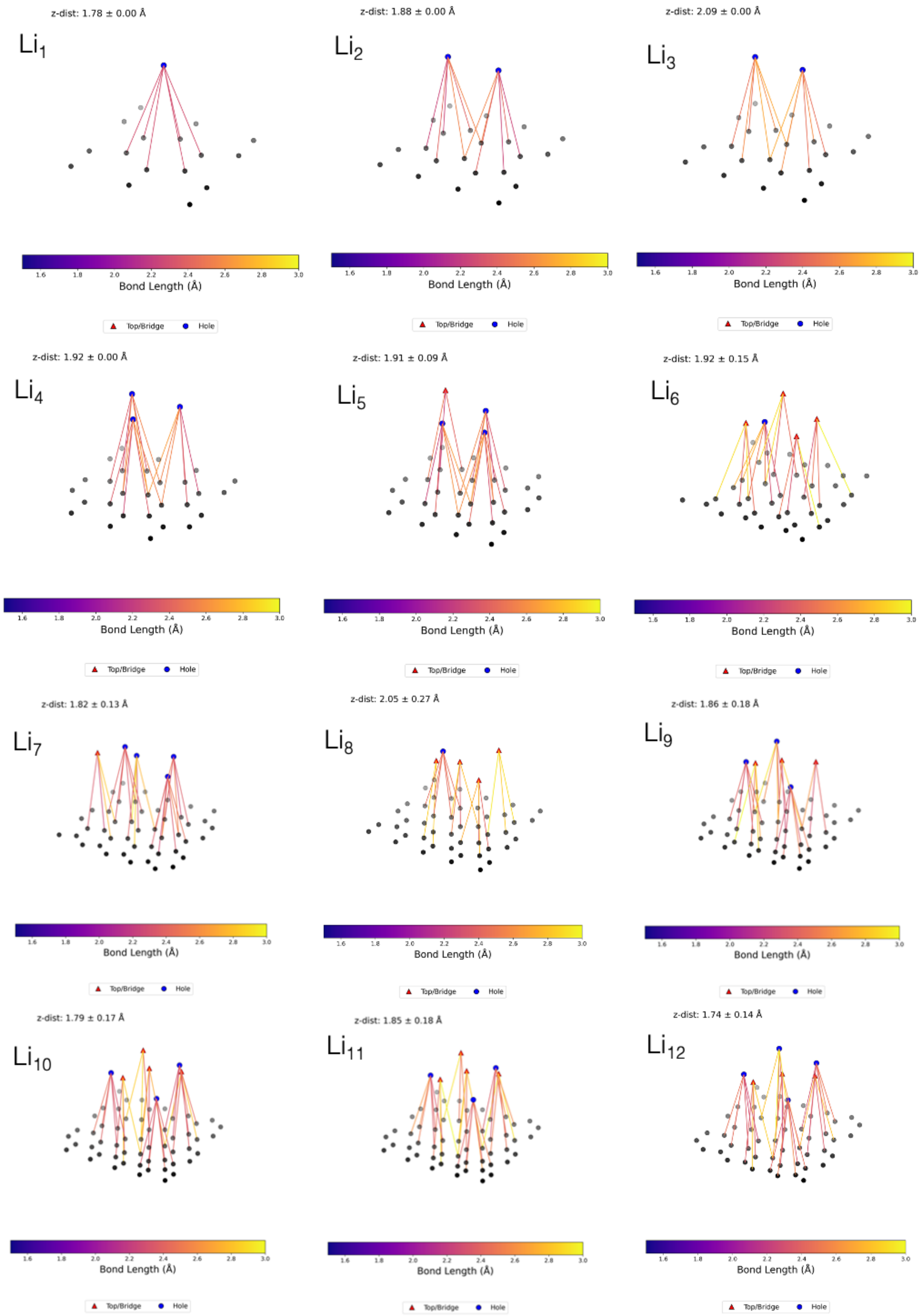

Figure S7. Li-C bond distances for Li_n clusters on pristine graphene. z-dist denotes the distance between Li and the graphene sheet.

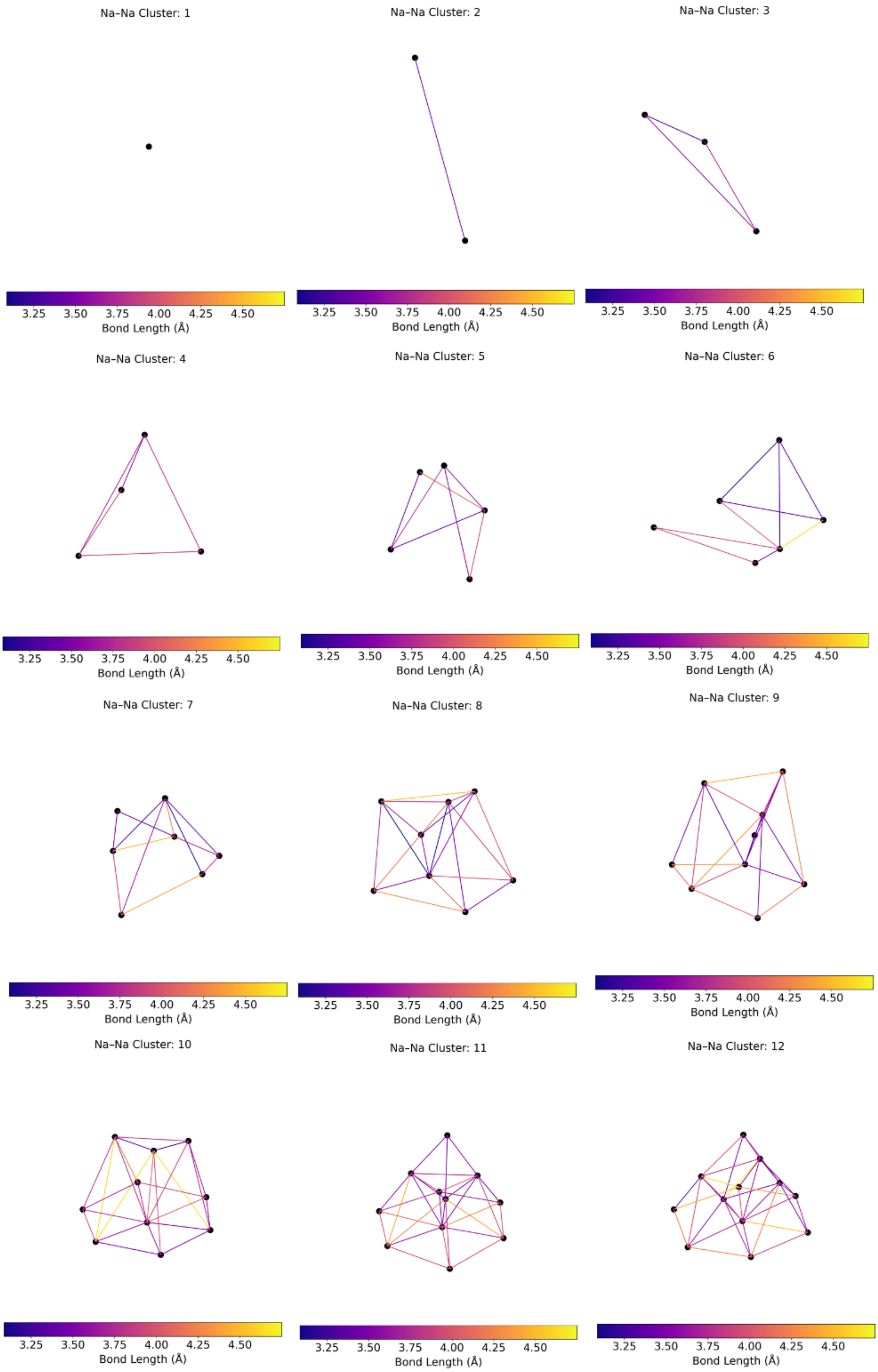

Figure S8. Na-Na distances in Na_n clusters on pristine graphene. The graphene Na_n distances are included in Figure S9.

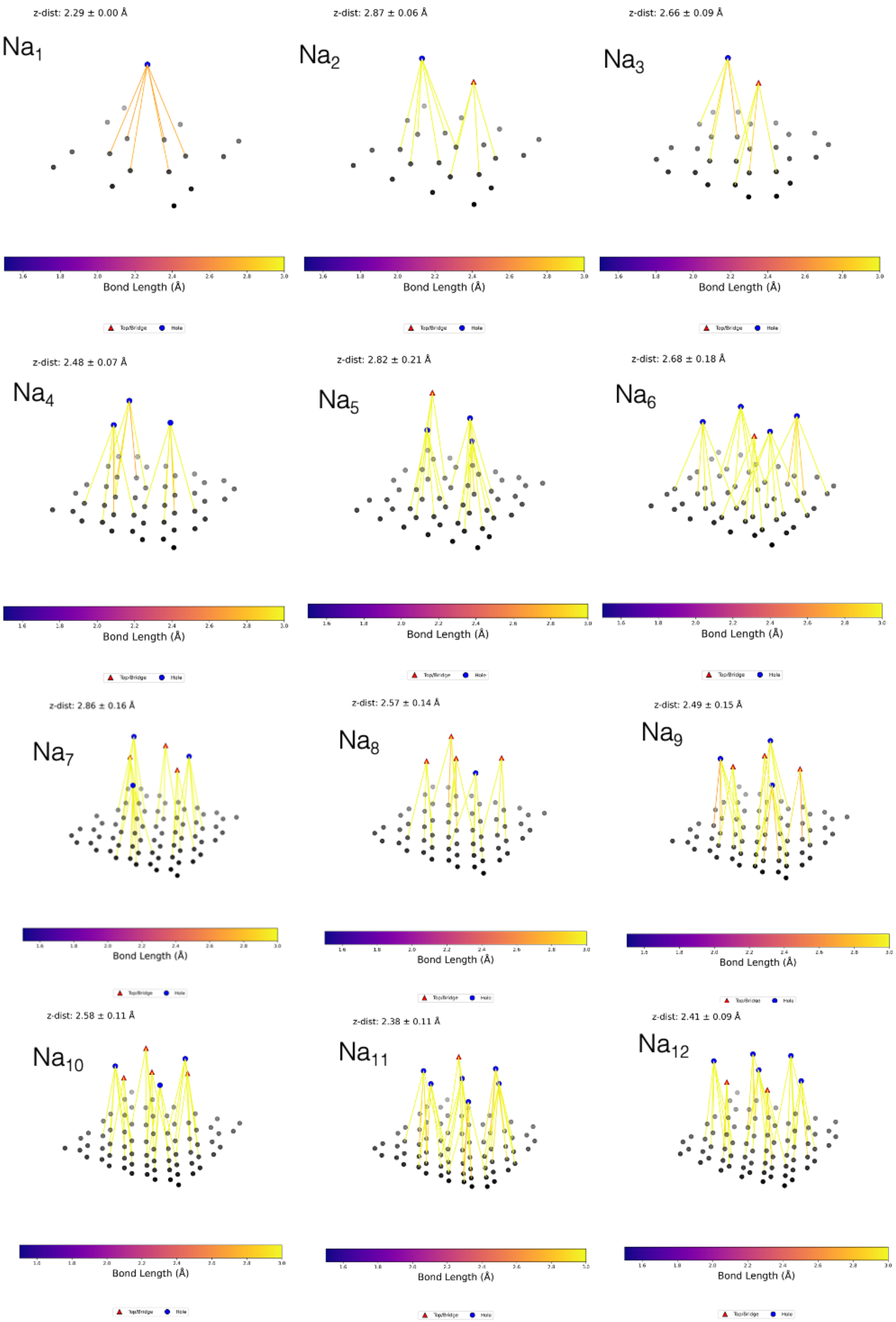

Figure S9. Na-C bond distances for Na_n clusters on pristine graphene. z-dist denotes the distance between Na and the graphene sheet.

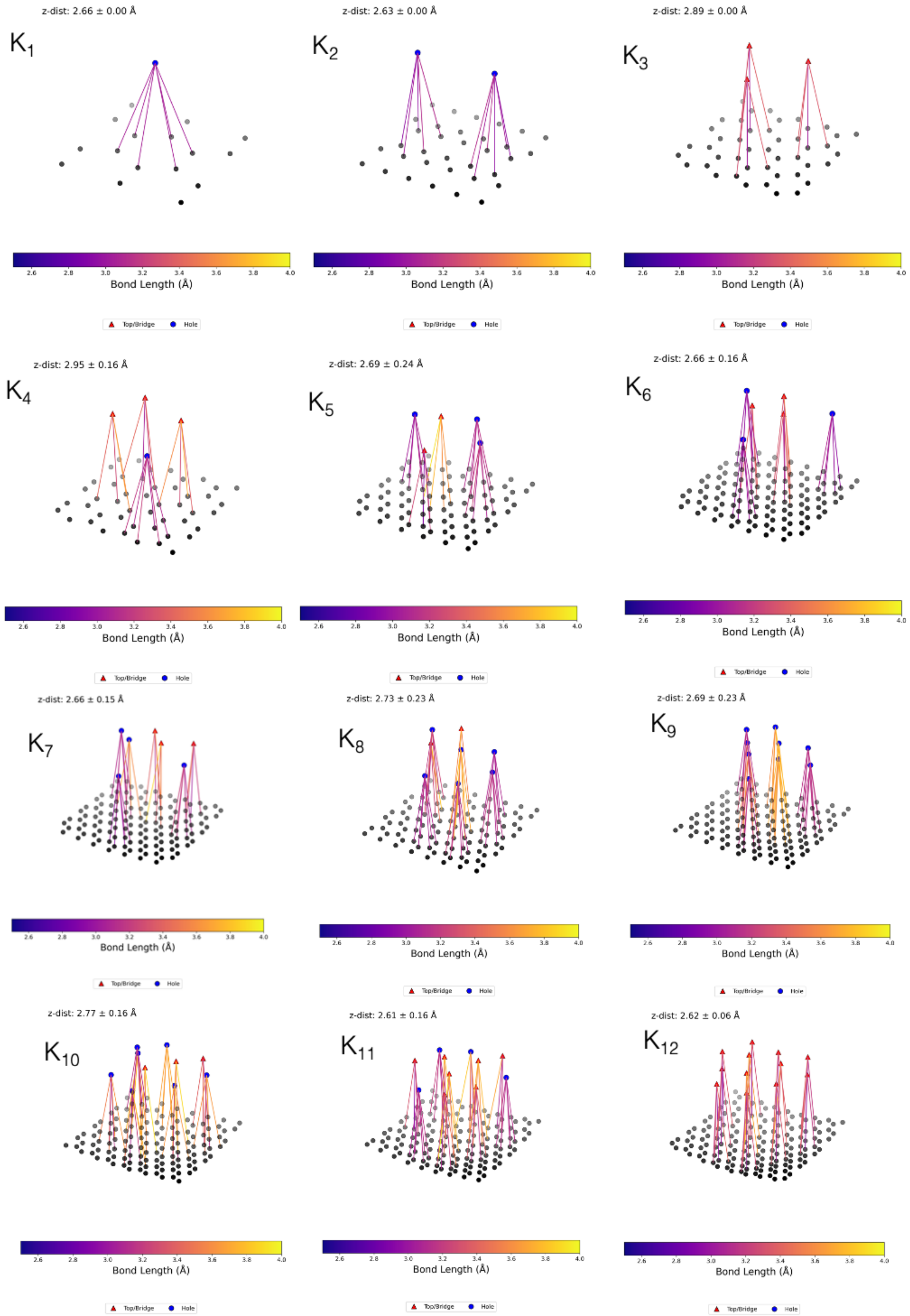

Figure S10. K-C bond distances for K_n clusters on pristine graphene. z-dist denotes the distance between K and the graphene sheet.

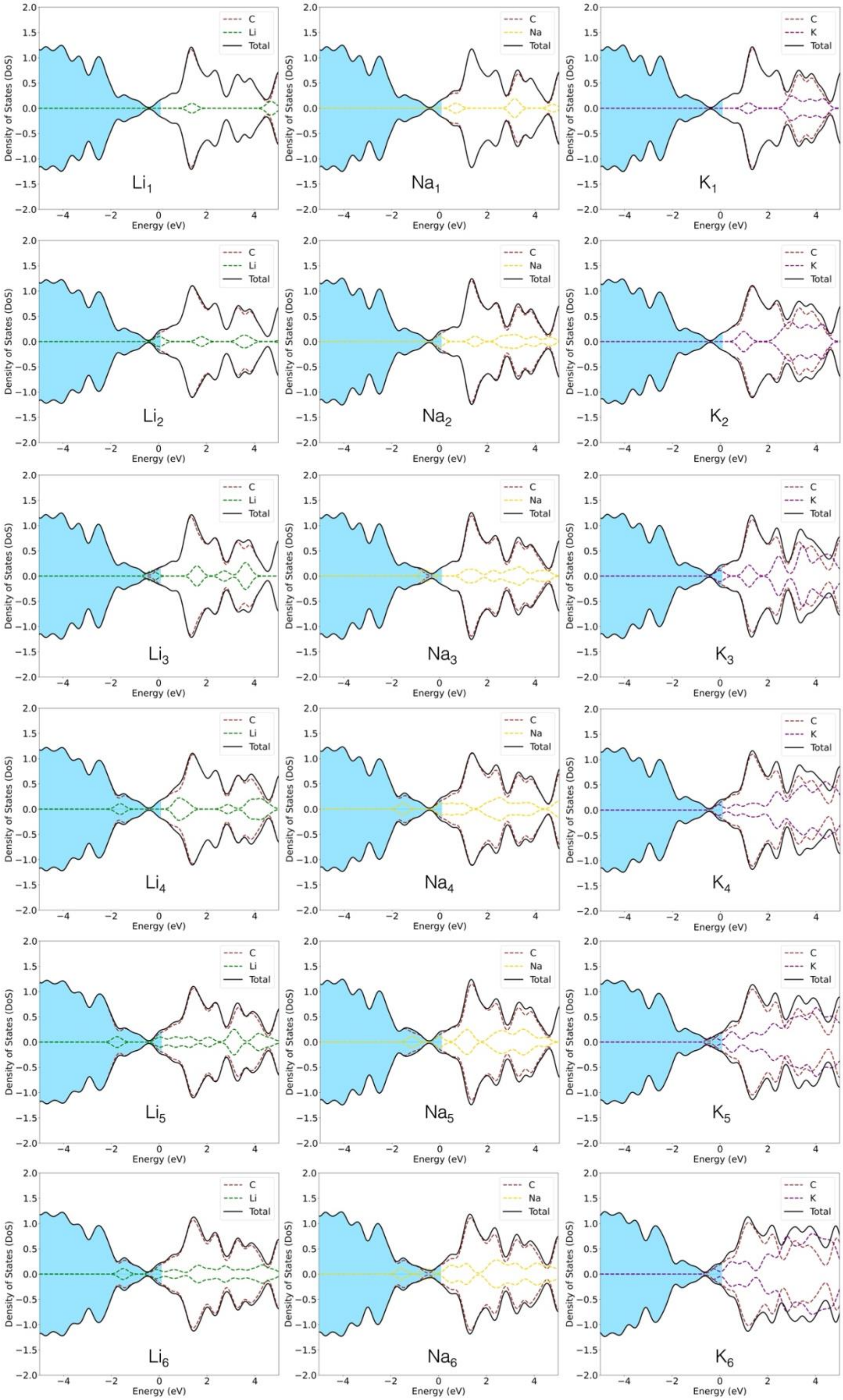

Li$_1$
Na$_1$
K$_1$
Li$_2$
Na$_2$
K$_2$
Li$_3$
Na$_3$
K$_3$
Li$_4$
Na$_4$
K$_4$
Li$_5$
Na$_5$
K$_5$
Li$_6$
Na$_6$
K$_6$
C
Li
Na
K
Total
Density of States (DoS)
Energy (eV)

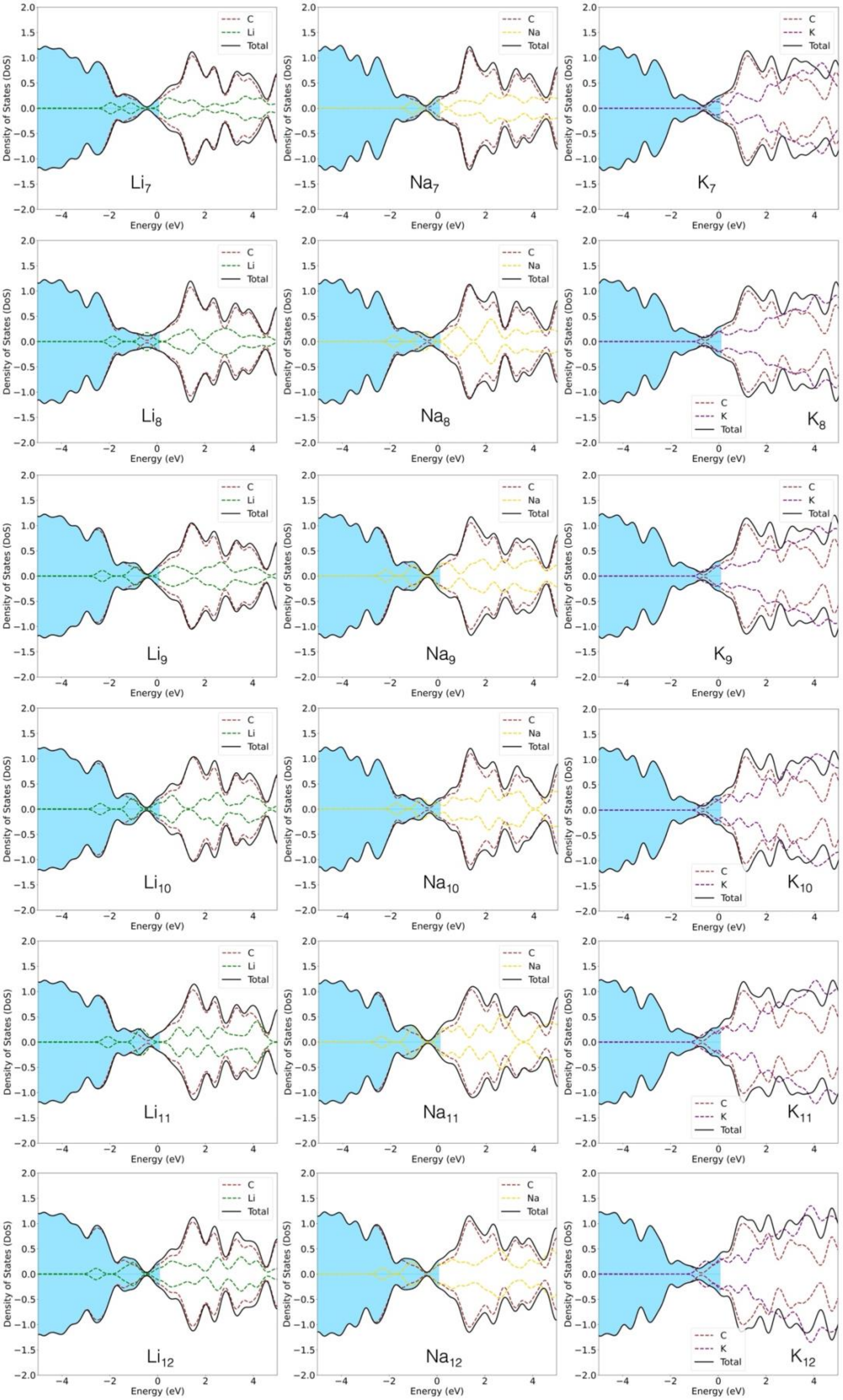

Figure S11. Projected density of states for M_n clusters on pristine graphene.

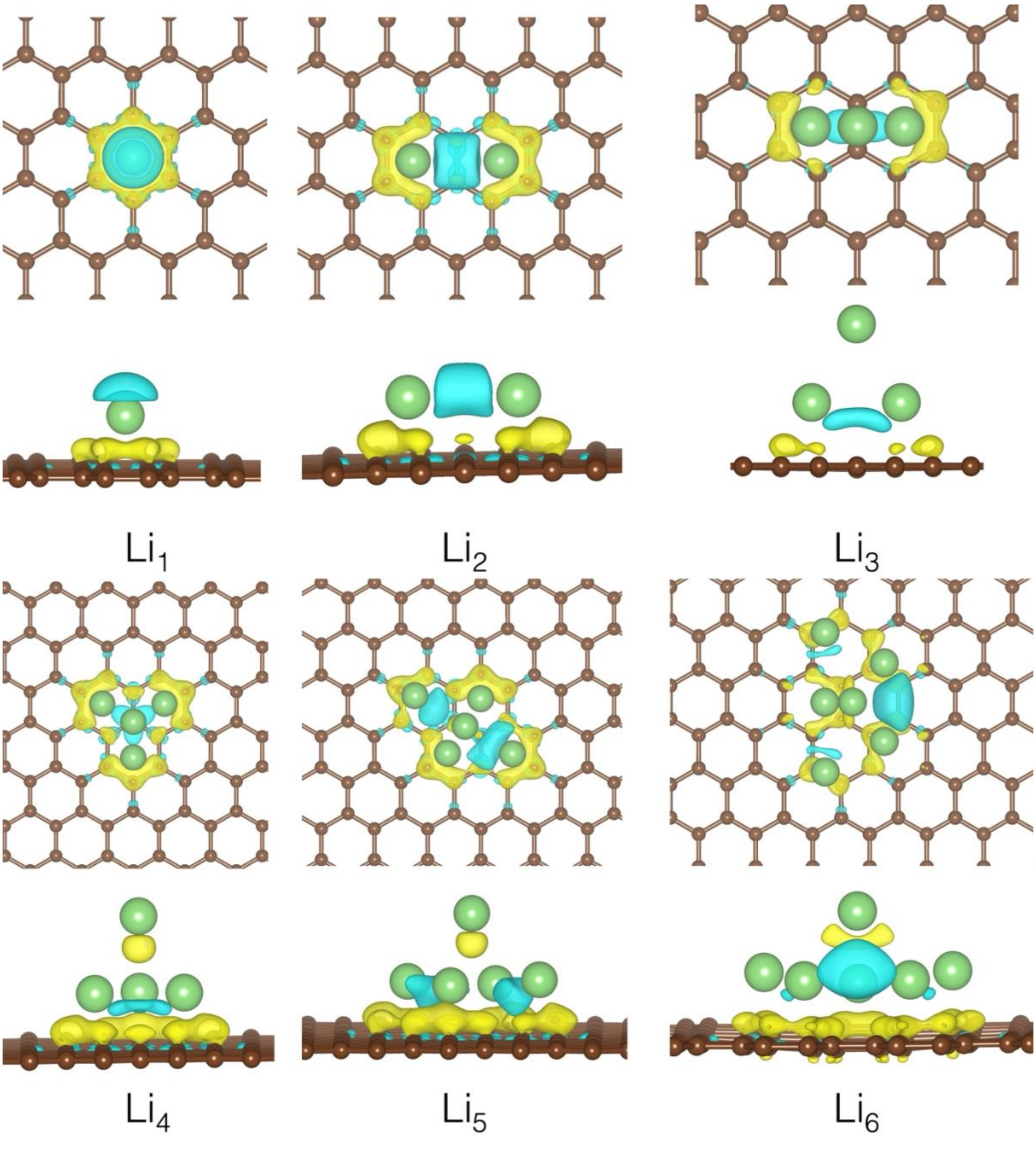
Li_1
Li_2
Li_3
Li_4
Li_5
Li_6

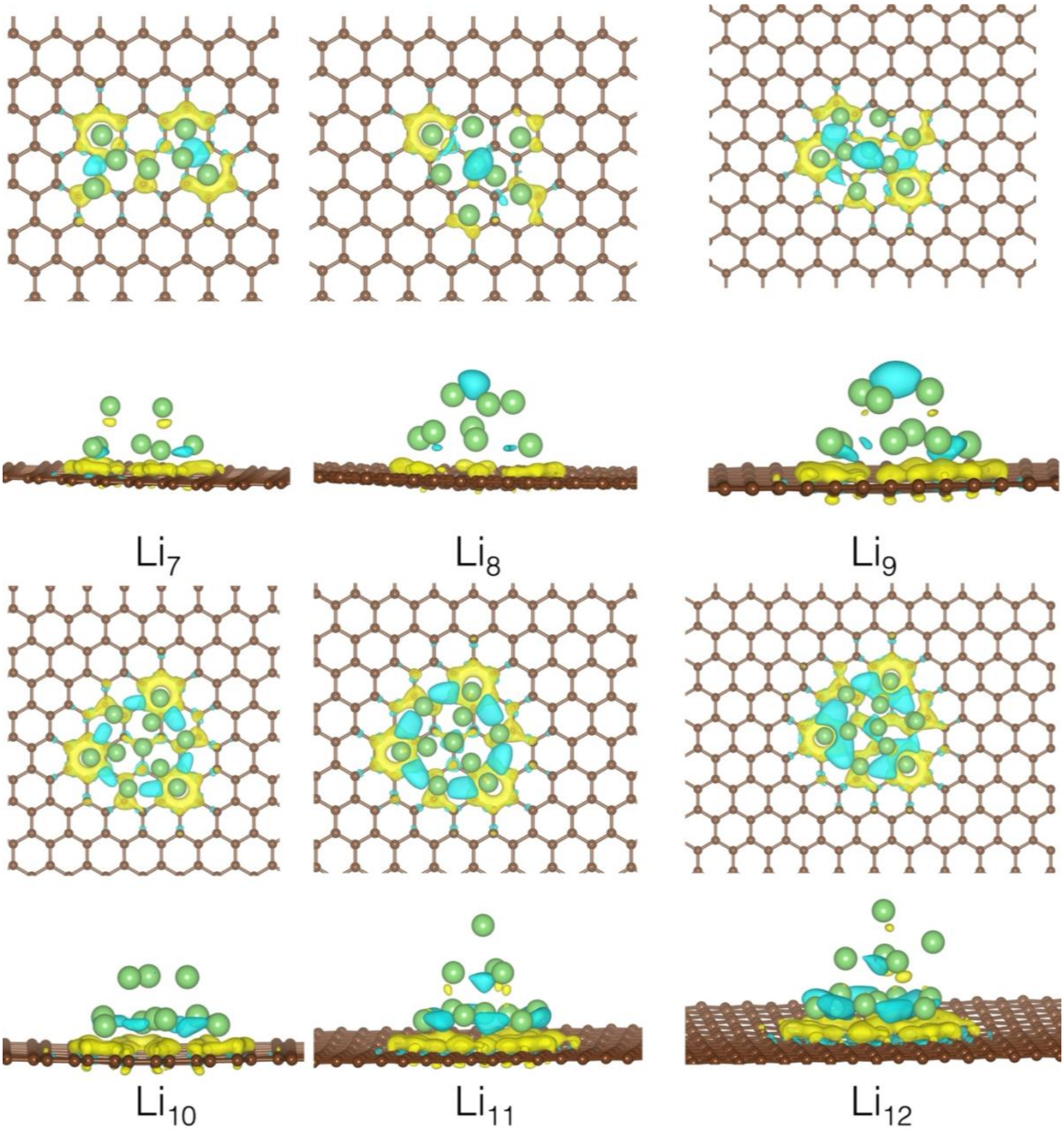

Figure S12. Charge density differences for Li clusters n=1-12 on pristine graphene. The first and third rows show the top views, and the second and fourth side views. Li is represented by green spheres and C with brown. Iso-surface is 0.003 for Li_{1-7}, 0.0025 for Li_{8-10}, and 0.0025 for Li_{11-12}. Yellow iso-surface denotes gain in charge density, whereas blue denotes loss.

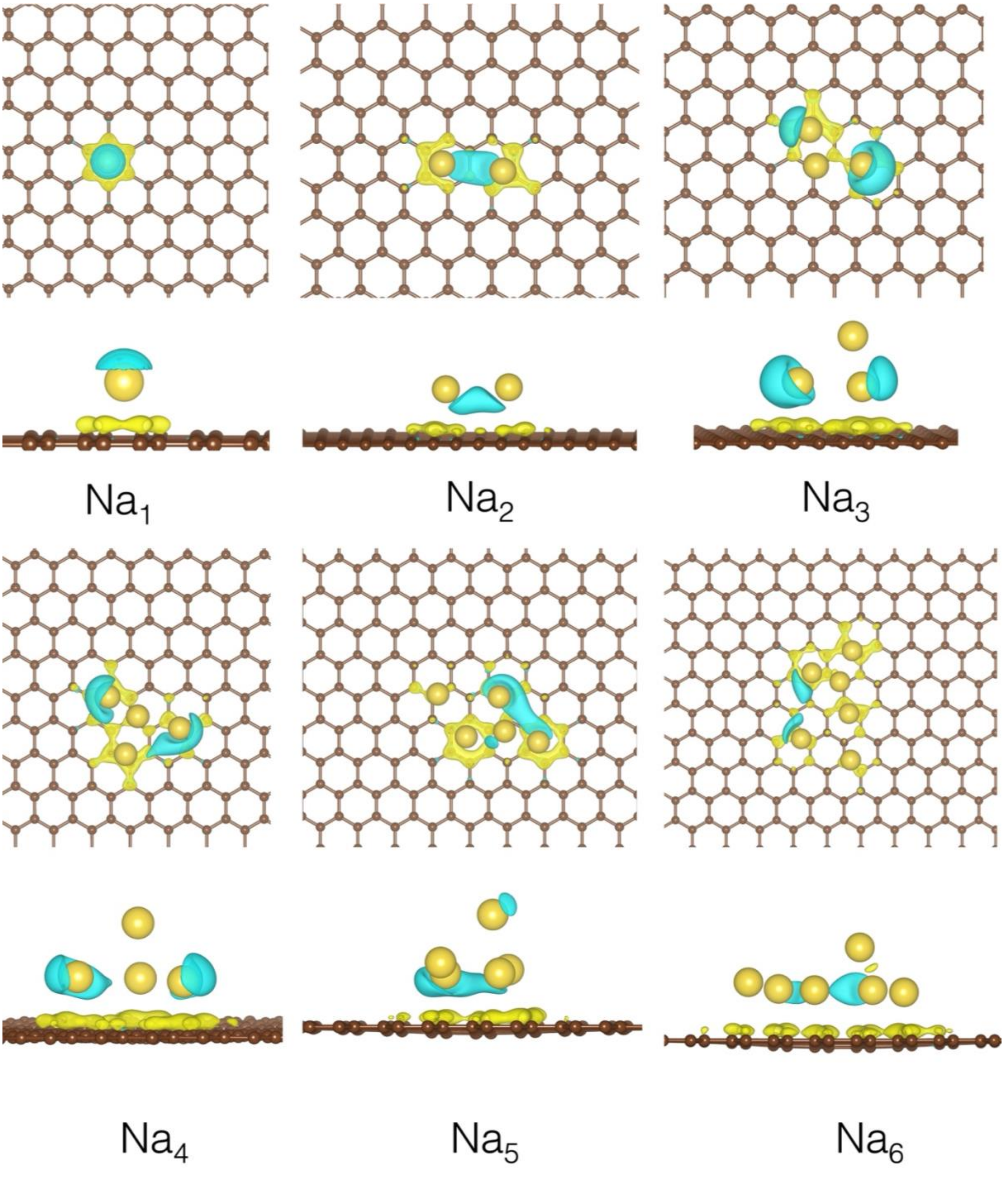
Na_1
Na_2
Na_3
Na_4
Na_5
Na_6

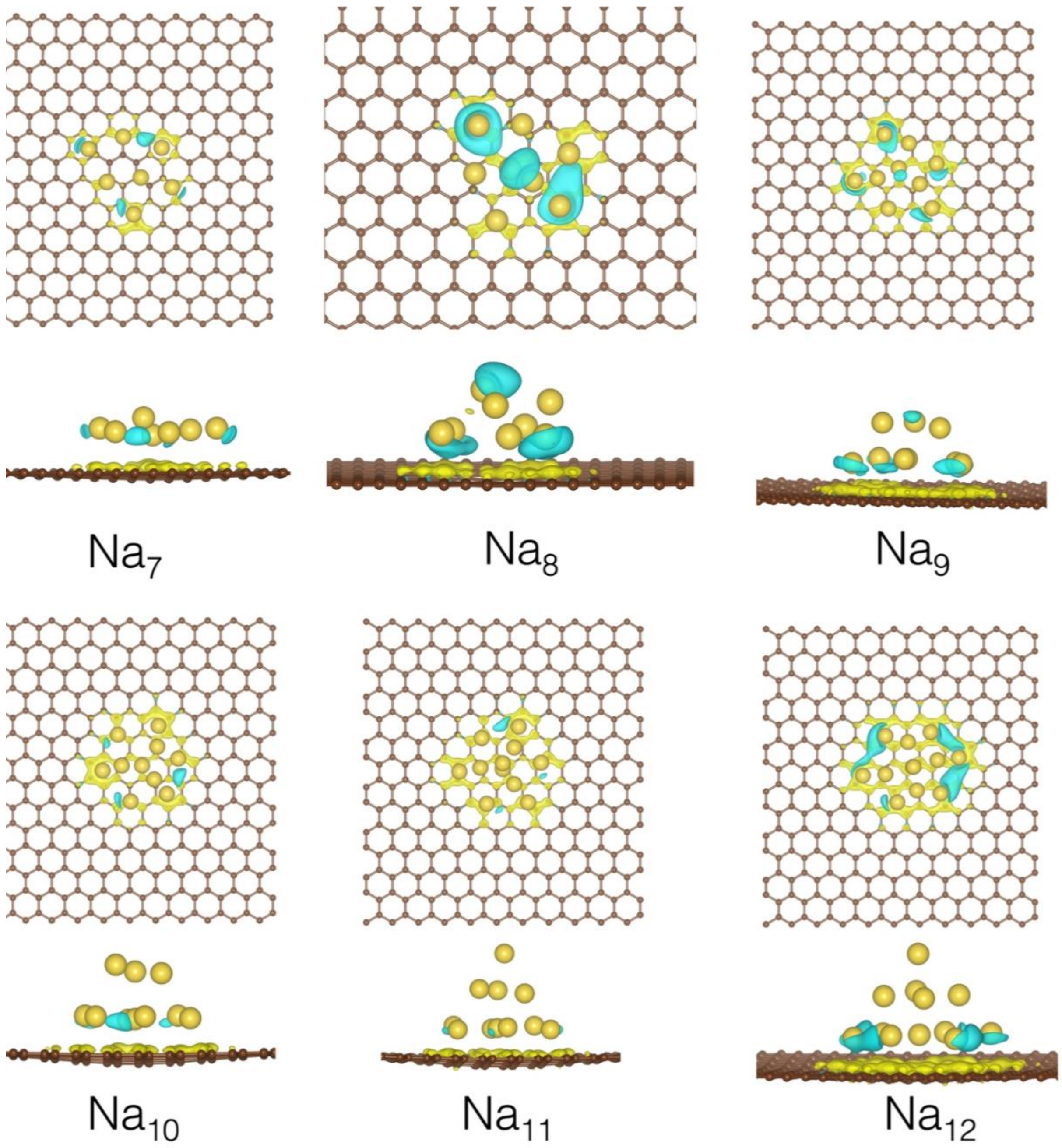

Figure S13. Charge density differences for Na clusters n=1-12 on pristine graphene. The first and third rows show the top views, and the second and fourth side views. Na is represented by yellow spheres and C with brown. Iso-surface is 0.003 for Na_1, 0.0015 for $Na_{2,3,5,7,8,10,12.}$, 0.002 for $Na_{4,11}$, and 0.0018 for $Na_{6,9.}$ Yellow iso-surface denotes gain in charge density, whereas blue denotes loss.

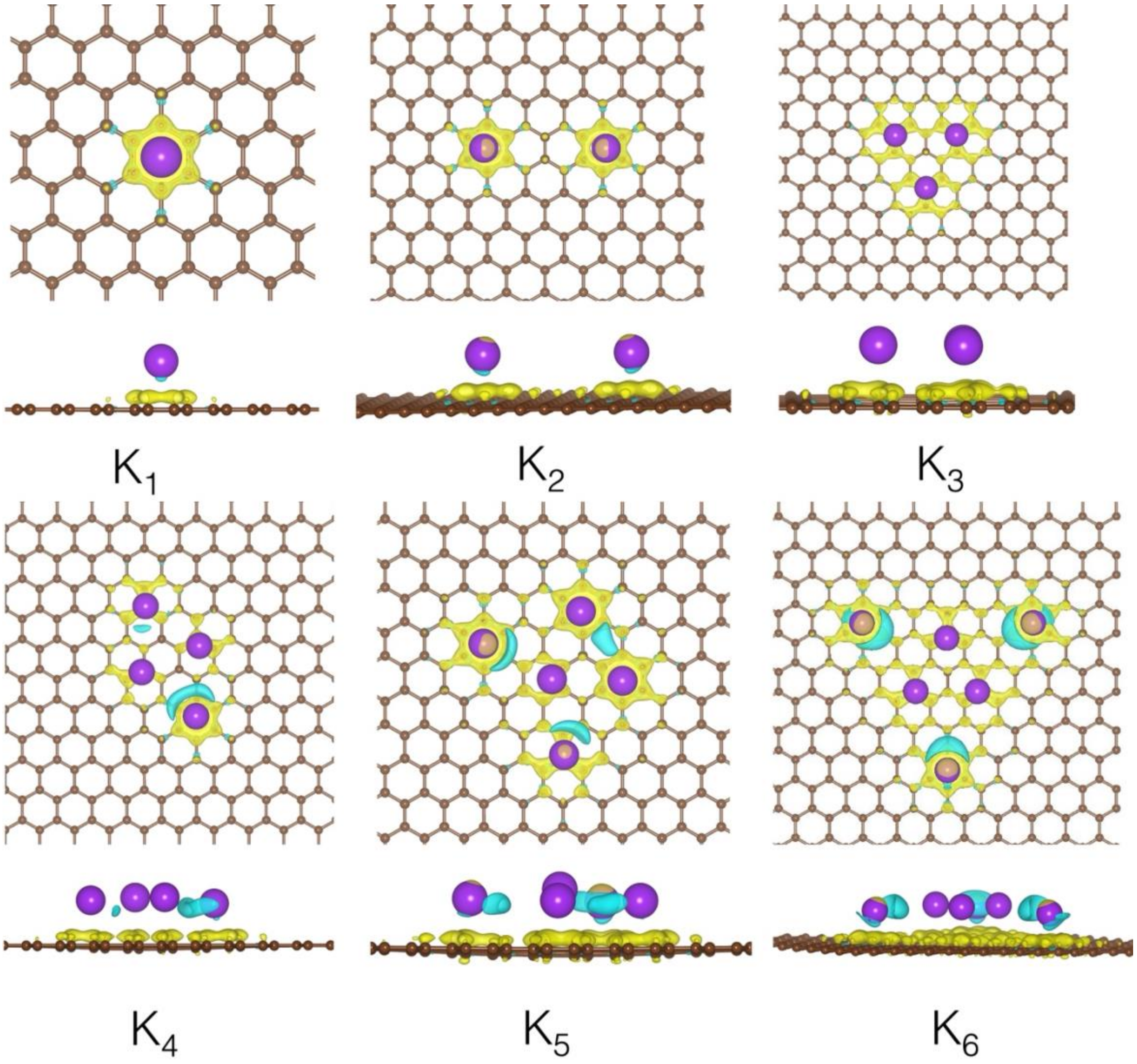
K_1
K_2
K_3
K_4
K_5
K_6

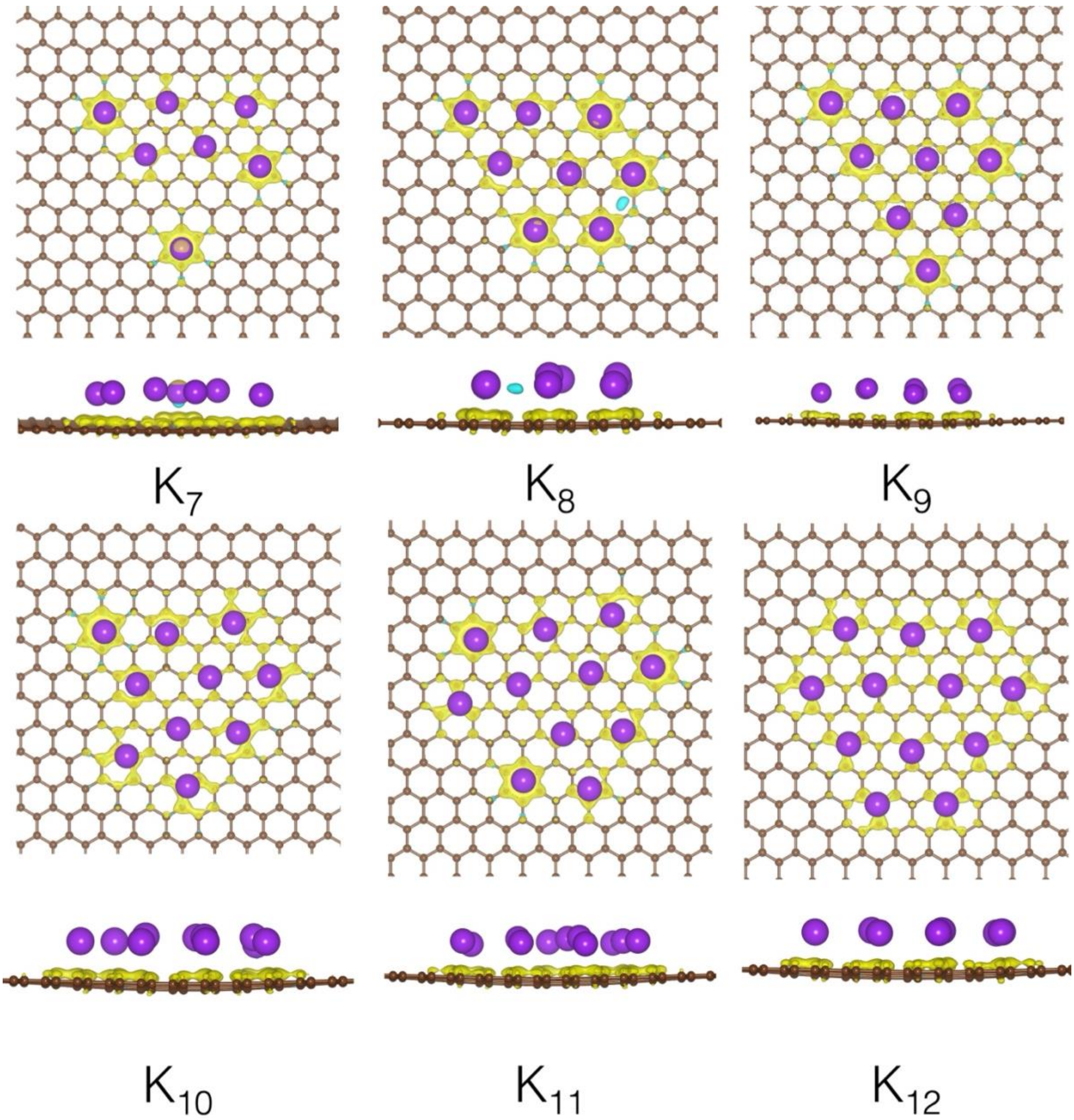

Figure S14. Charge density differences for K clusters n=1-12 on pristine graphene. The first and third rows show the top views, and the second and fourth side views. K is represented by purple spheres and C with brown. Iso-surface is 0.0012 for K_3, 0.0015 for K_{4-6}, 0.0018 for $K_{1,2,8,9,11,12}$, and 0.0015 for K_{10}. Yellow iso-surface denotes gain in charge density, whereas blue denotes loss.

S3 Defective graphene

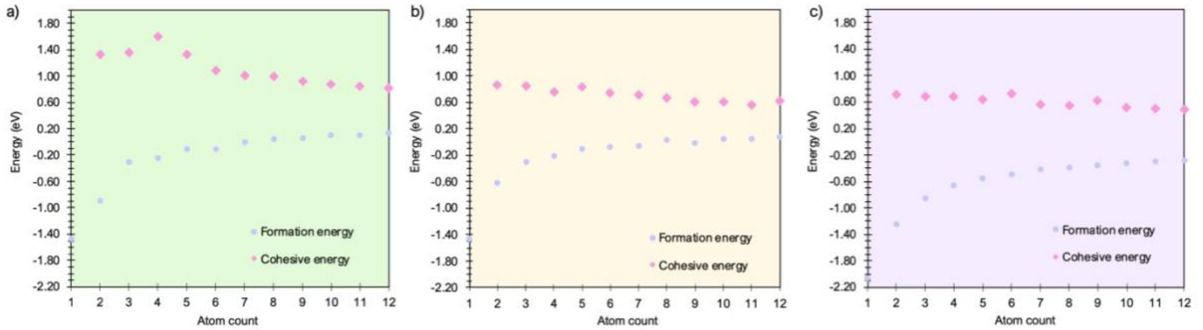

Figure S15. Interaction energies on the defective graphene sheet decomposed into binding energy, cohesive energy, and formation energy with metal chemical potential plotted for Li in (a), Na in (b), and K in (c), with the atom count denoting the number of M atoms in the M_n clusters.

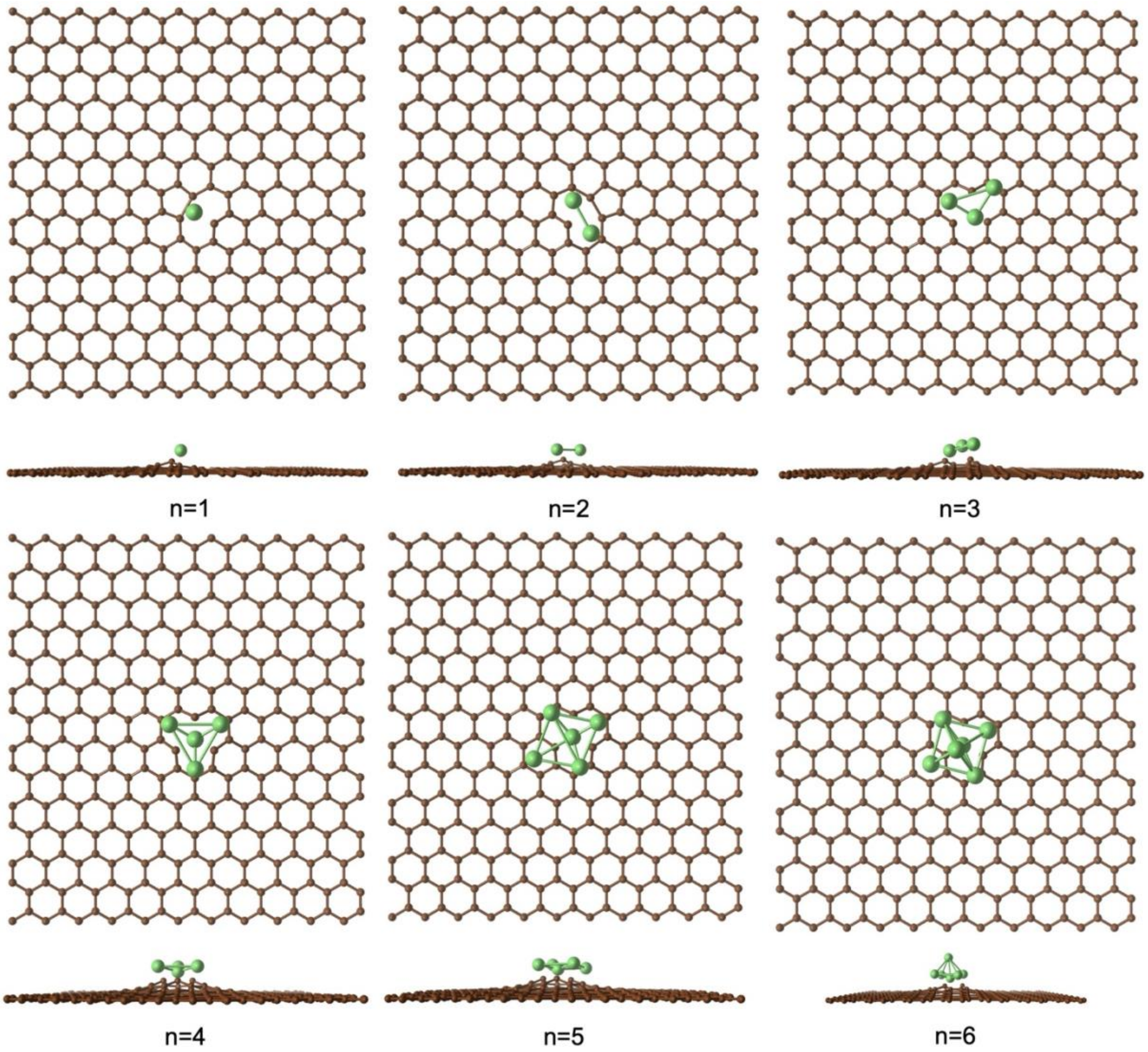

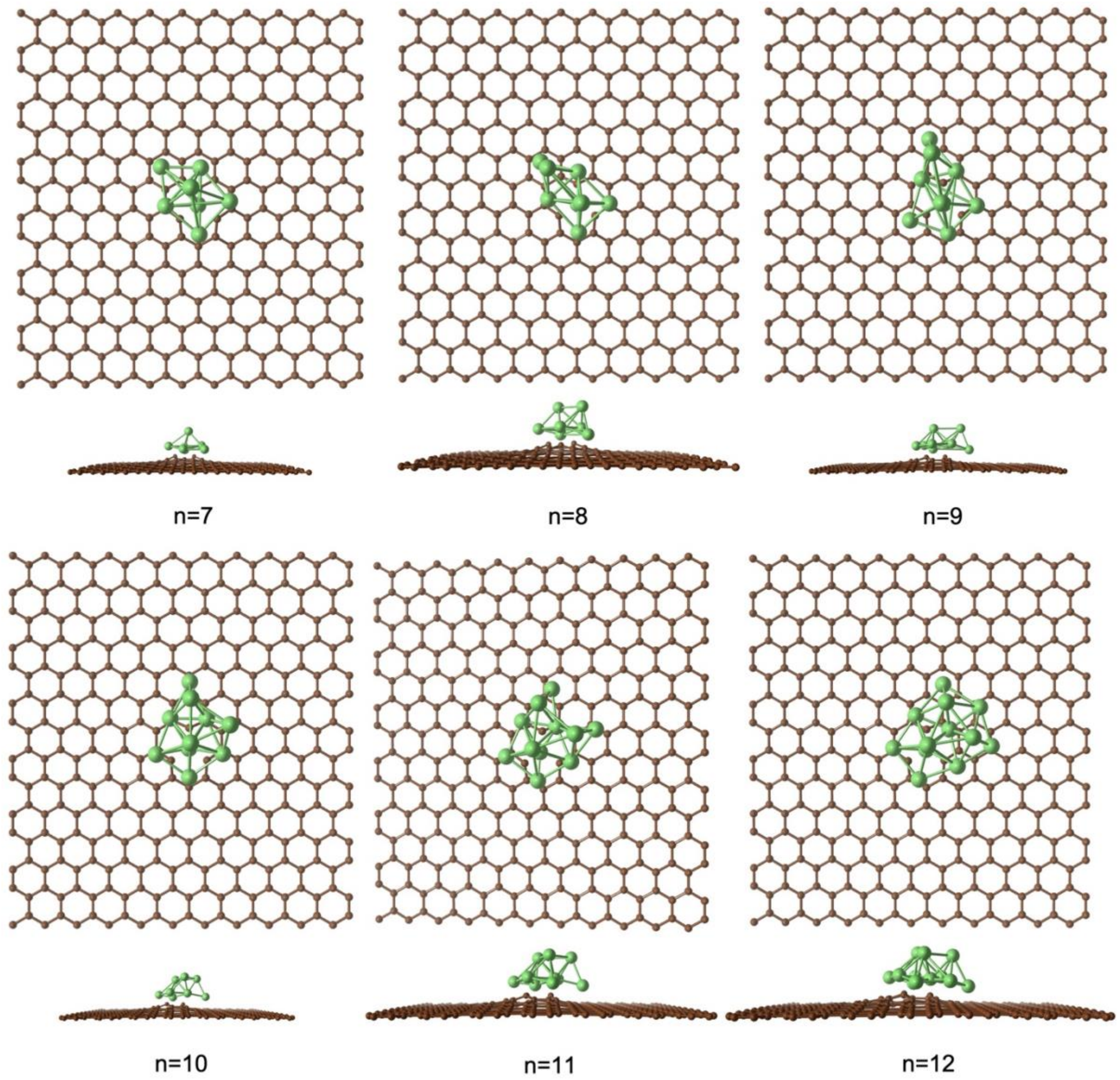

Figure S16. Top and side view of lowest energy Li_n (n=1-12) on defective basal plane for the data presented in Figure 4a in the main article. Brown spheres are carbon and green lithium.

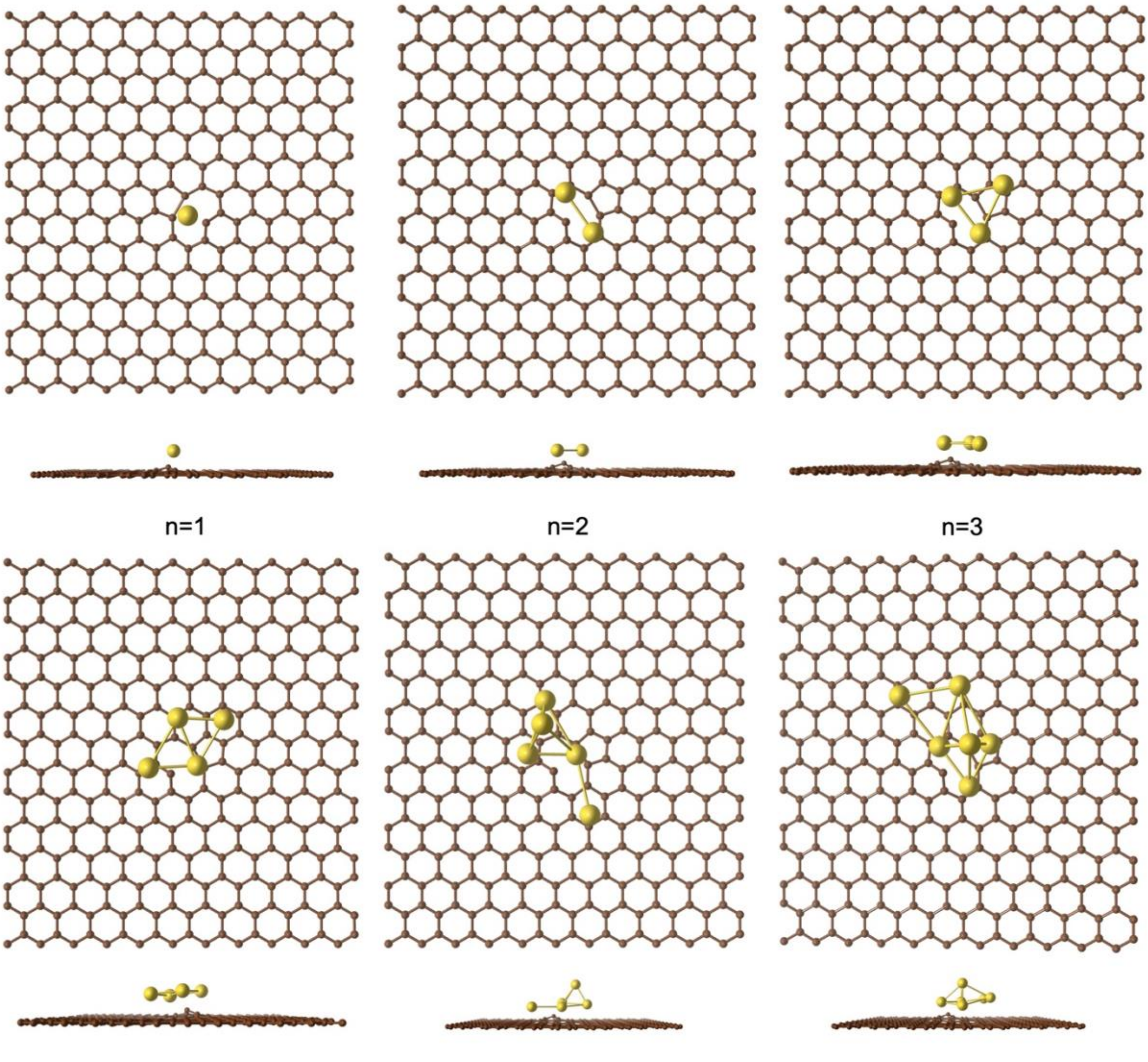
n=1
n=2
n=3
n=4
n=5
n=6

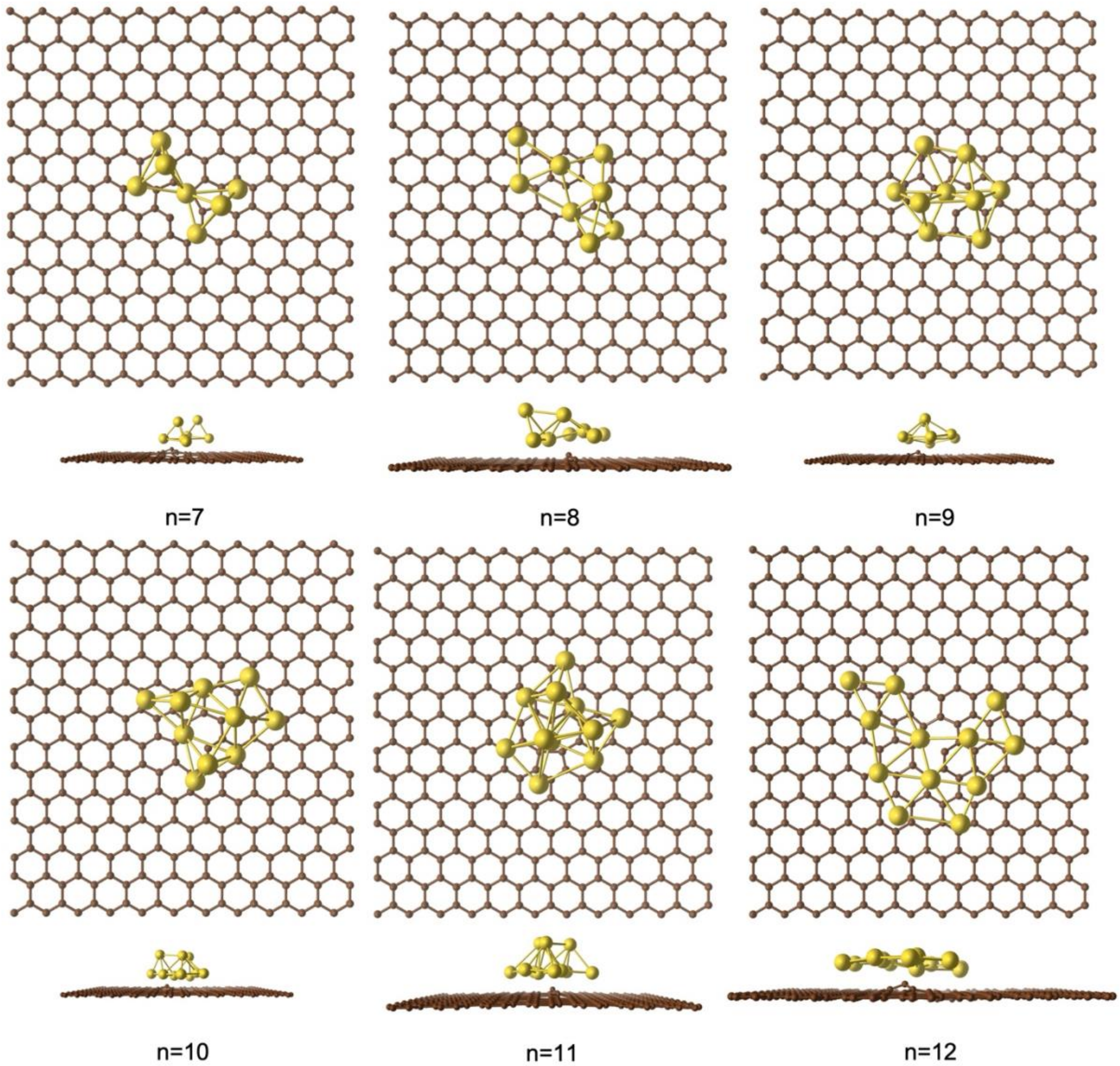

Figure S17. Top and side view of lowest energy Na_n (n=1-12) on defective basal plane for the data presented in Figure 4b in the main article. Brown spheres are carbon and yellow sodium.

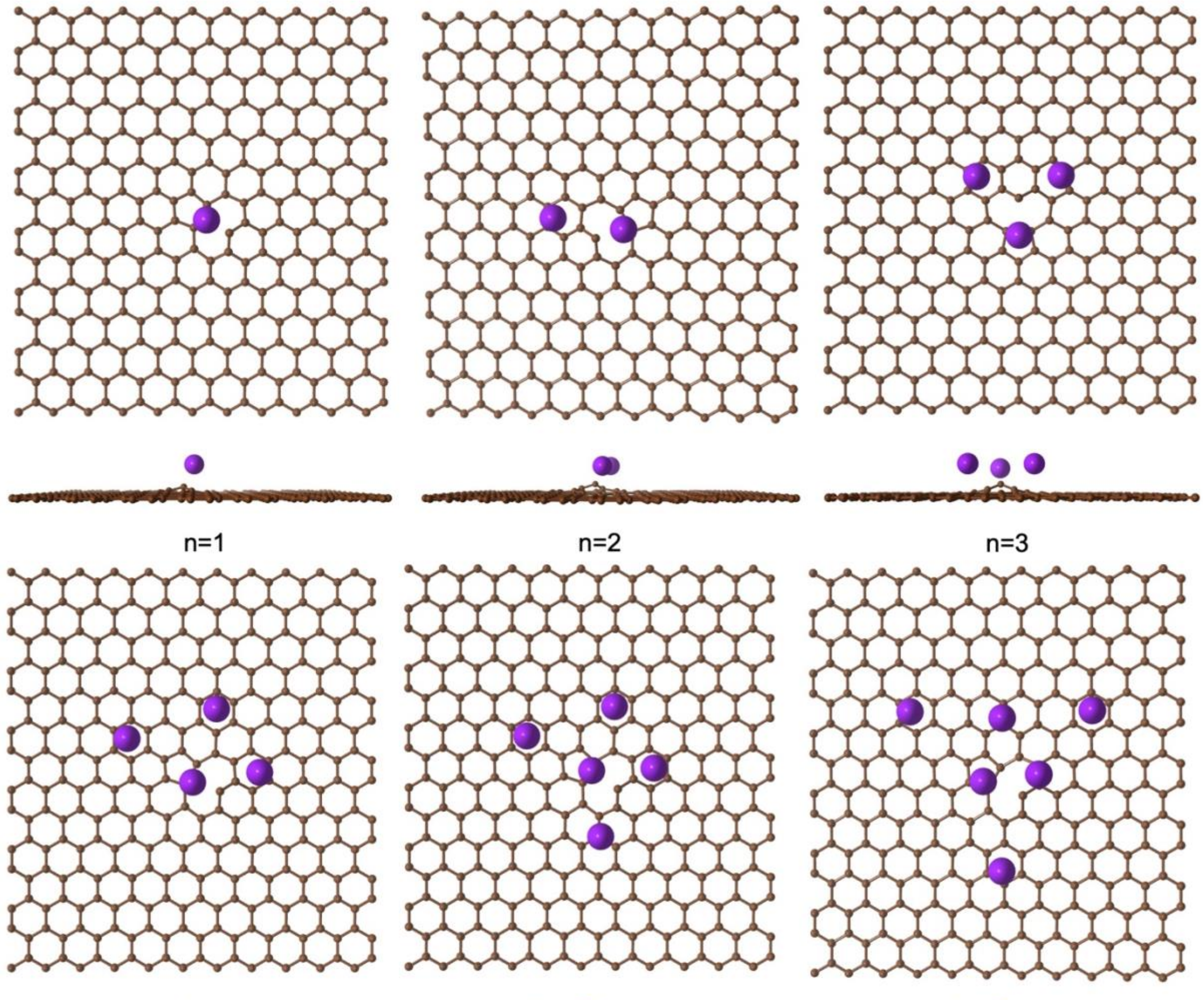
n=1
n=2
n=3
n=4
n=5
n=6

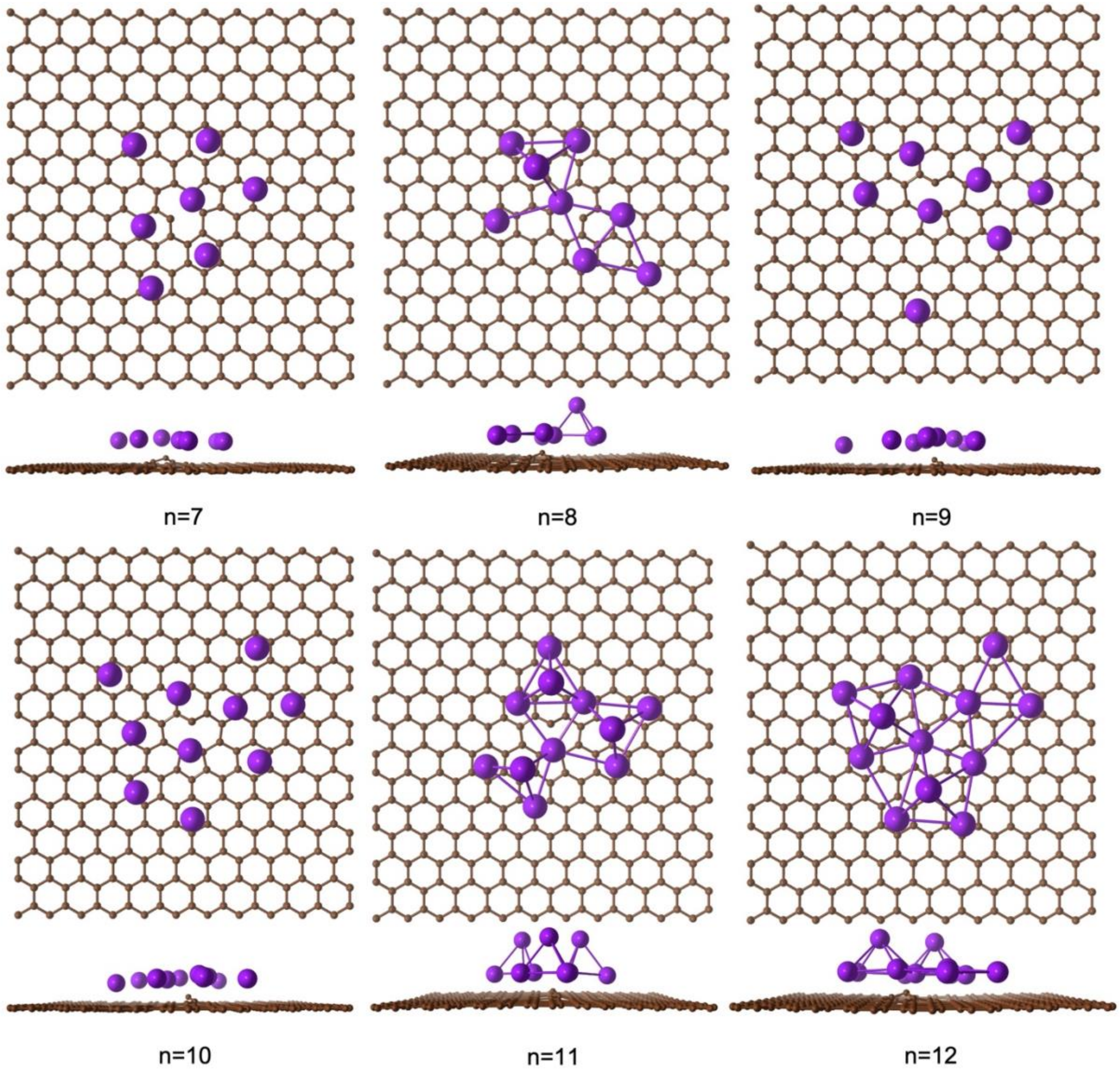

Figure S18. Top and side view of lowest energy K_n (n=1-12) on defective basal plane for the data presented in Figure 4c in the main article. Brown spheres are carbon and purple potassium.

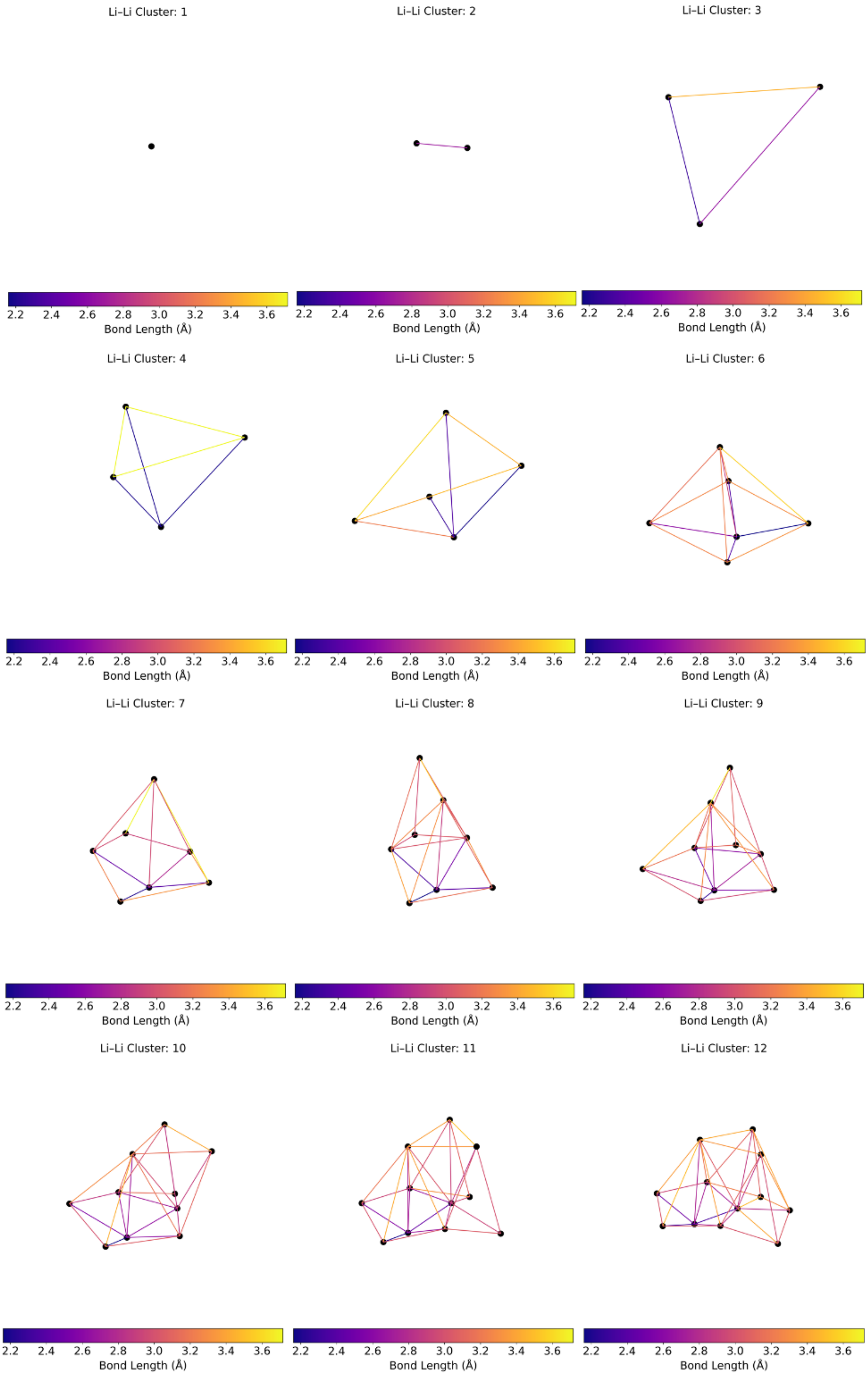

Figure S19. Li-Li bond distances for Li_n clusters on defective graphene. Li-C distances are plotted in Figure S20 below.

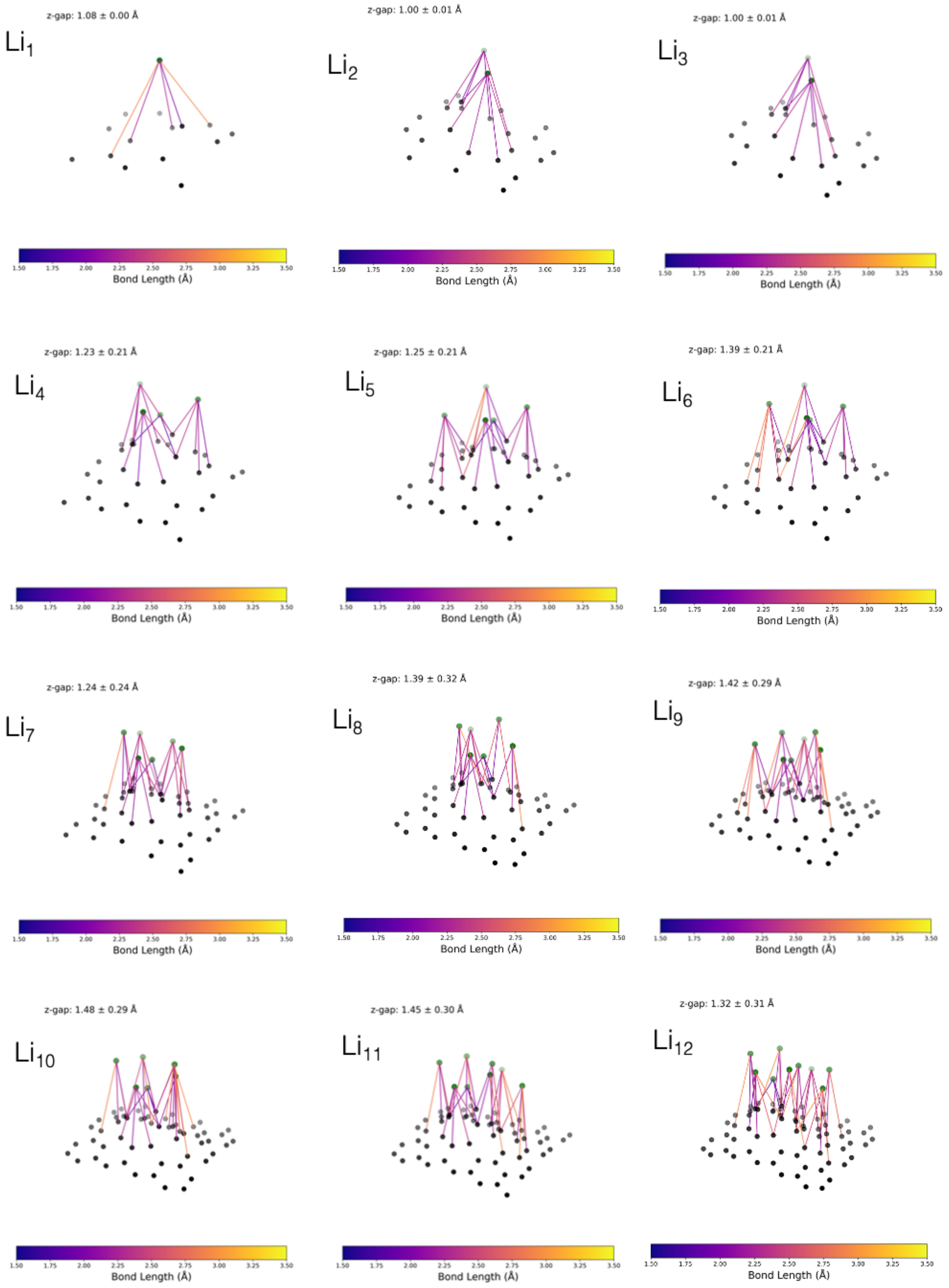

Figure S20. Li-C bond distances for Li_n clusters on defective graphene. z-dist denotes the distance between Li and the graphene sheet.

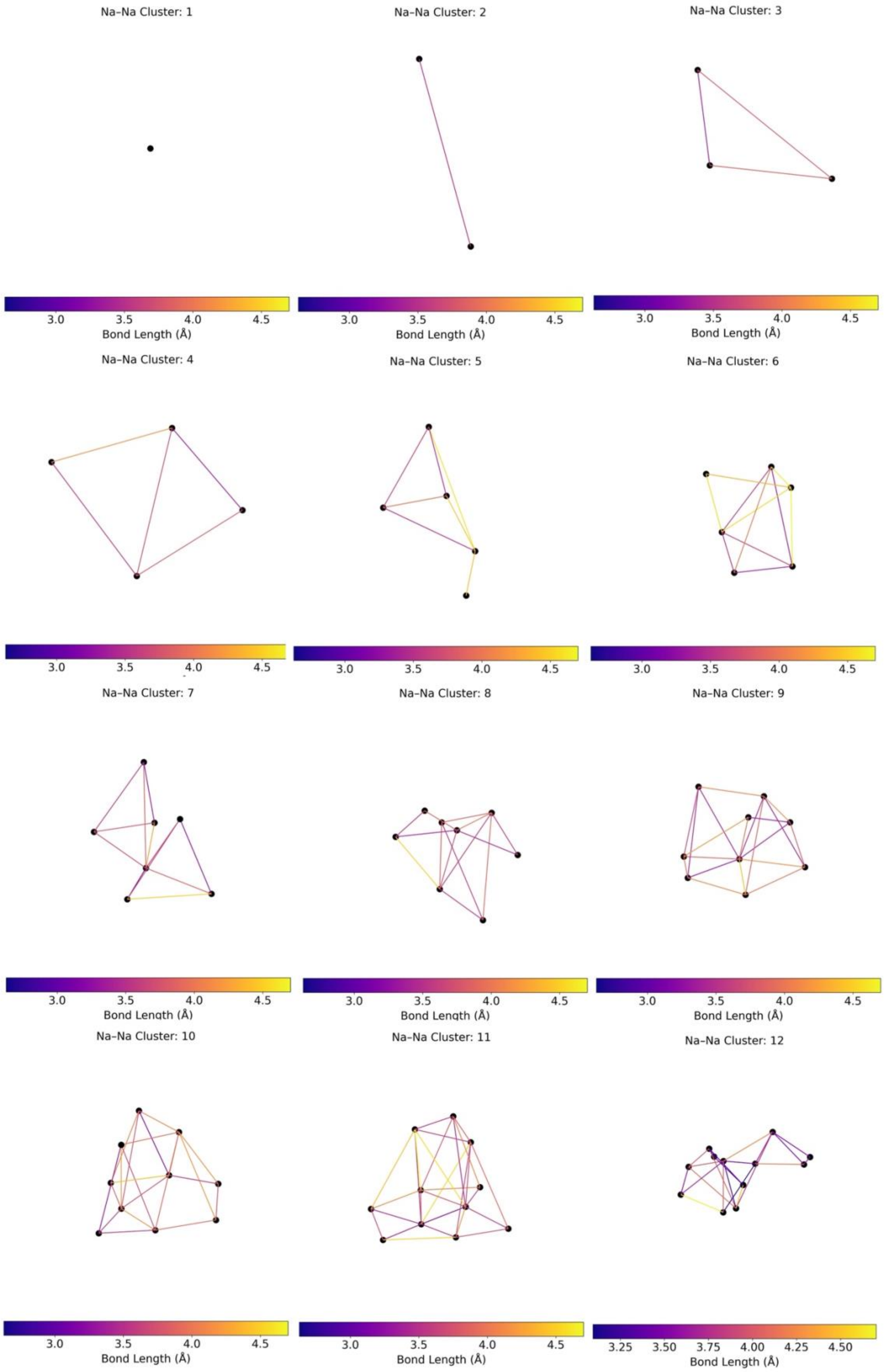

Figure S21. Na-Na bond distances for Na_n clusters on defective graphene. Na-C distances are plotted in Figure S22 below.

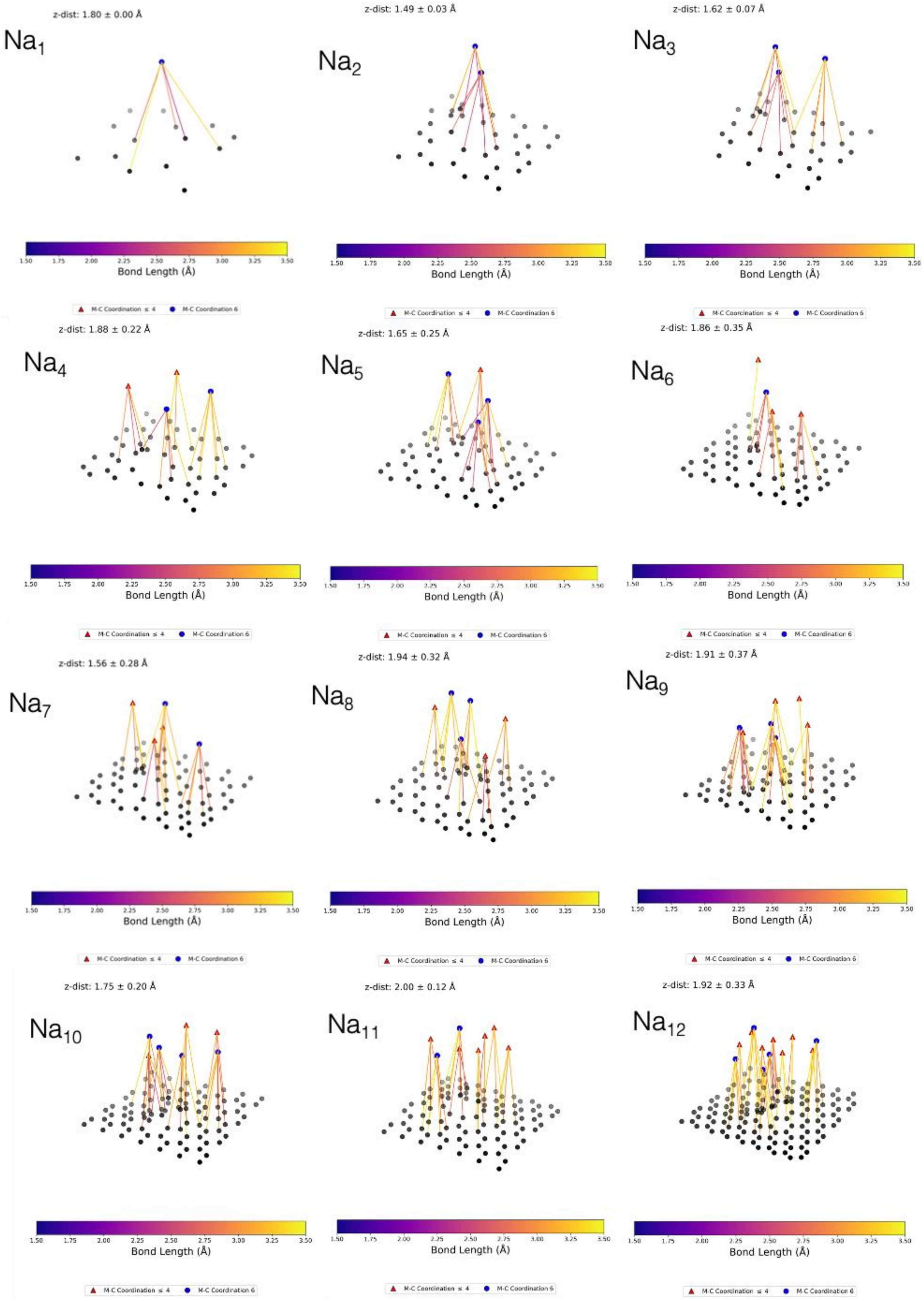

Figure S22. Na-C bond distances for Na_n clusters on defective graphene. z-dist denotes the distance between Na and the graphene sheet.

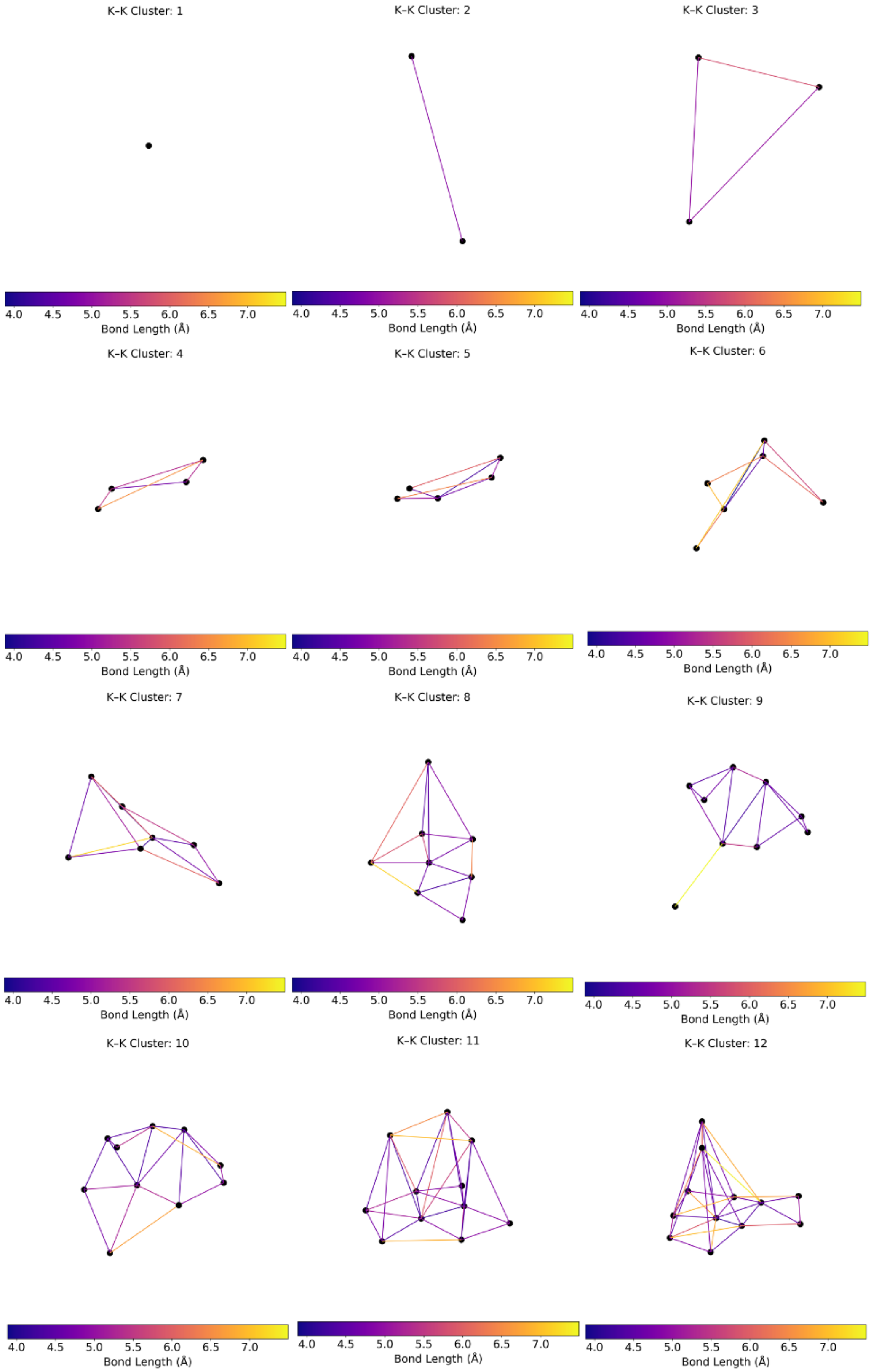

Figure S23. K-K bond distances for K_n clusters on defective graphene. K-C distances are plotted in Figure S24 below.

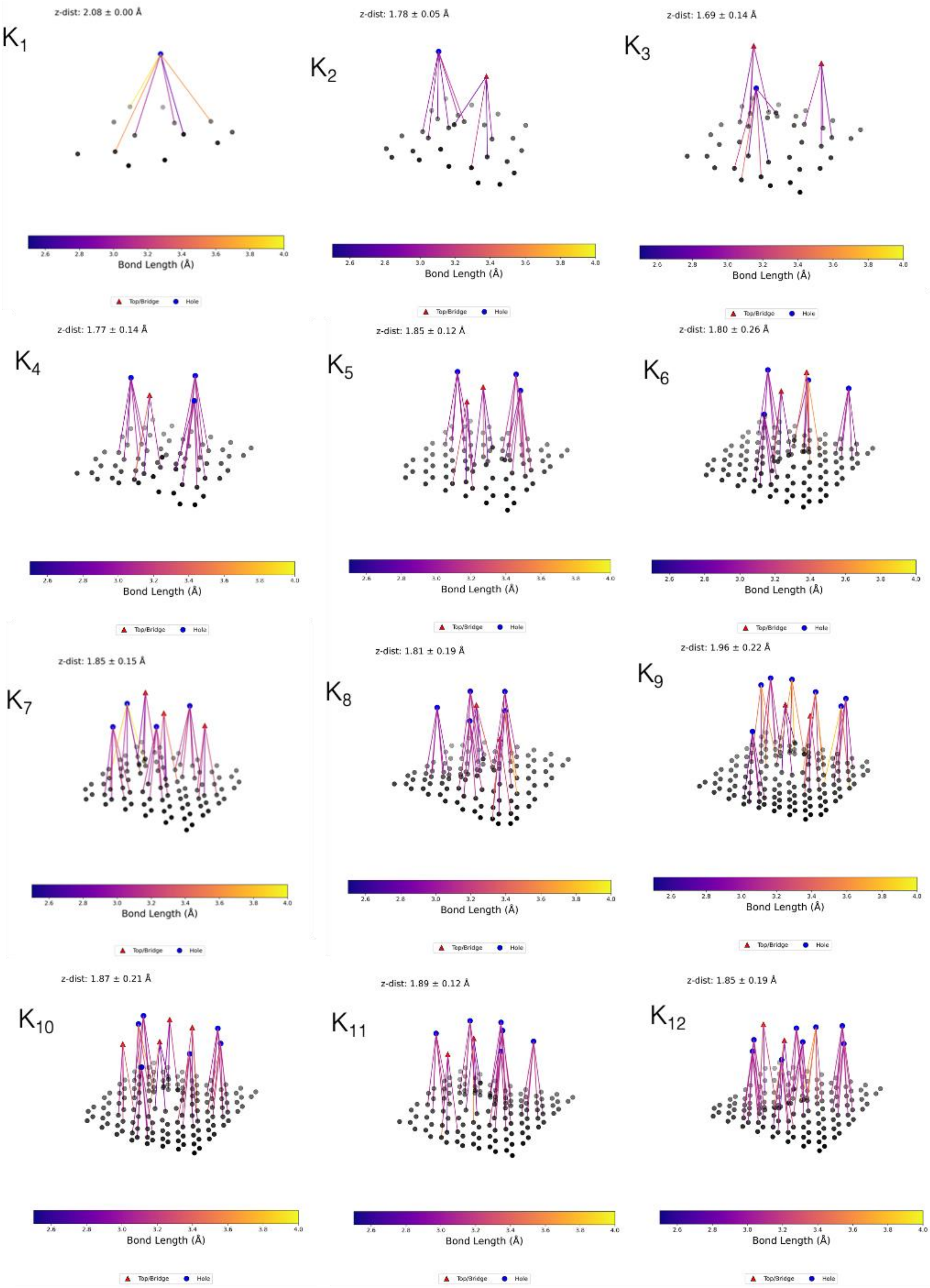

Figure S24. K-C bond distances for K_n clusters on defective graphene. z-dist denotes the distance between K and the graphene sheet.

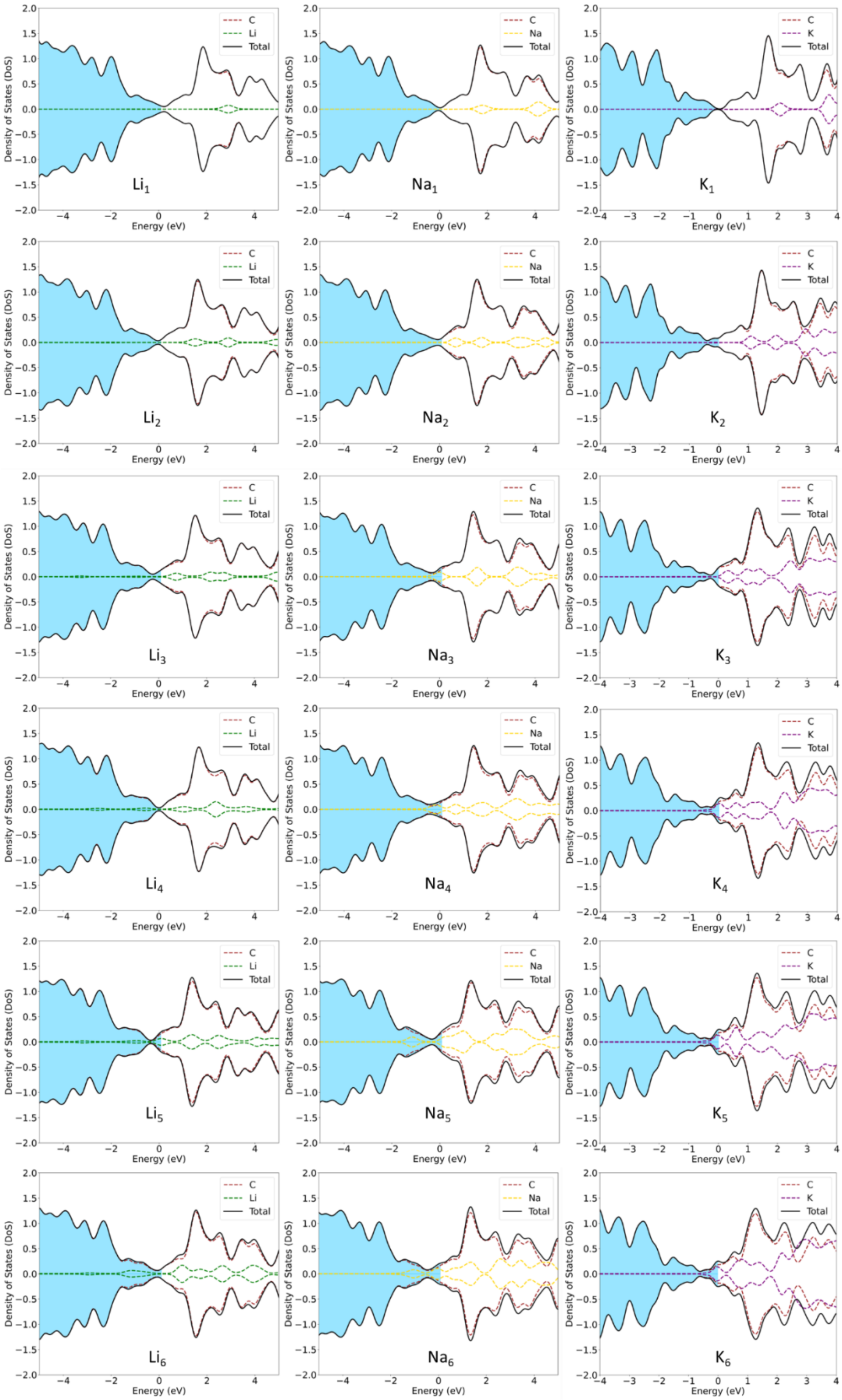
Li$_1$
Na$_1$
K$_1$
Li$_2$
Na$_2$
K$_2$
Li$_3$
Na$_3$
K$_3$
Li$_4$
Na$_4$
K$_4$
Li$_5$
Na$_5$
K$_5$
Li$_6$
Na$_6$
K$_6$
C
Li
Na
K
Total
Density of States (DoS)
Energy (eV)

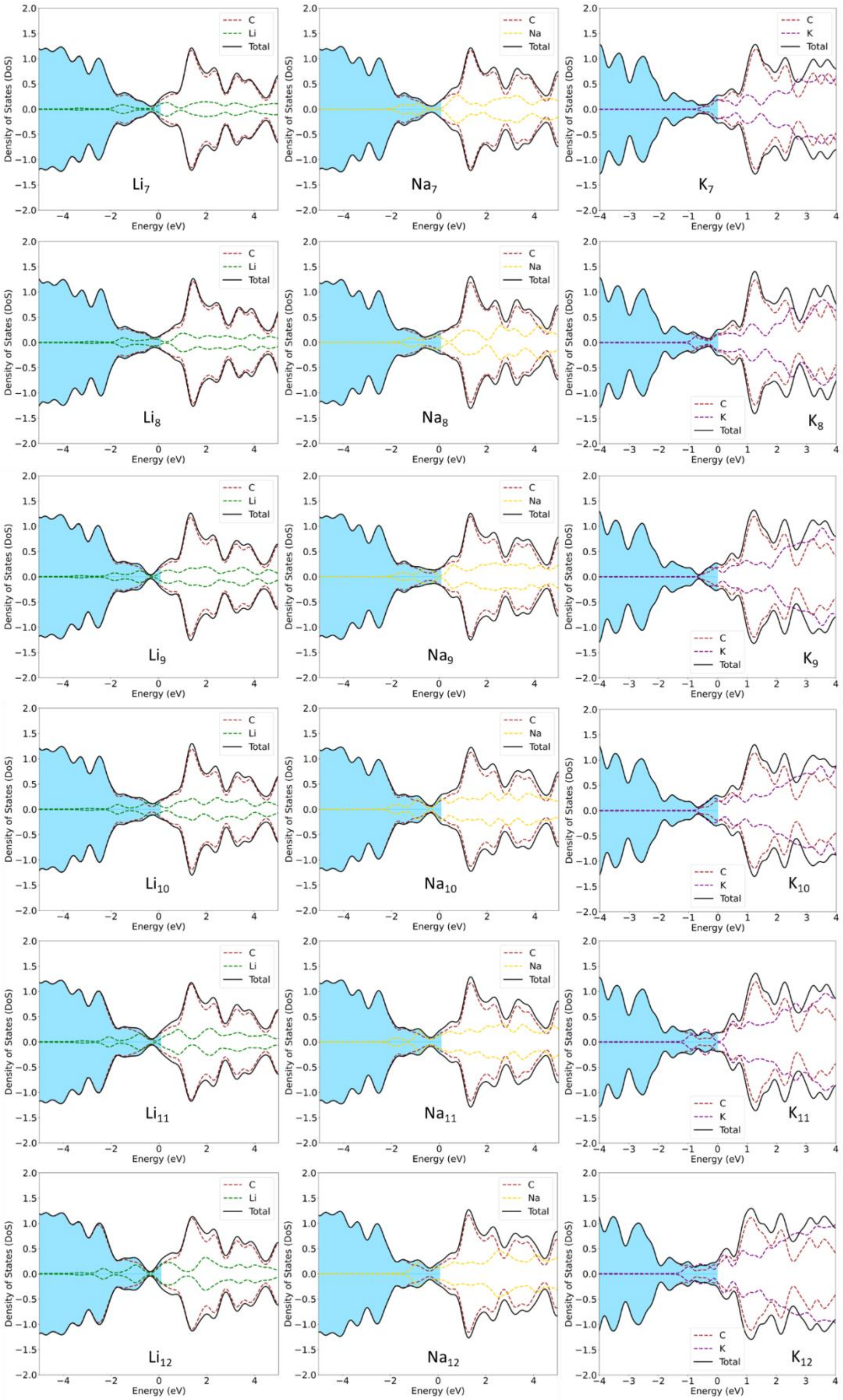

Figure S25. Projected density of states for M_n clusters on defective graphene.

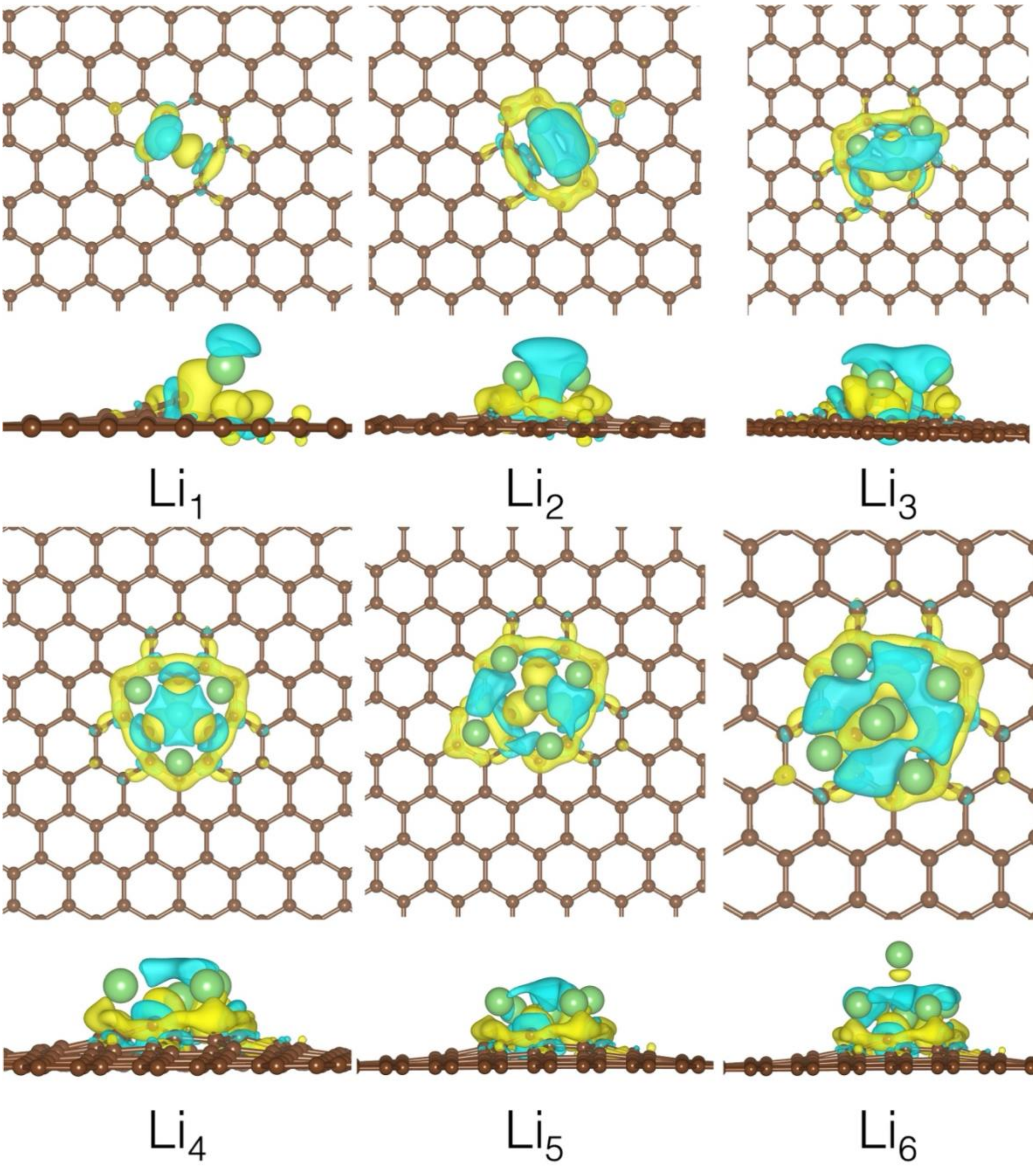

Li_1
Li_2
Li_3
Li_4
Li_5
Li_6

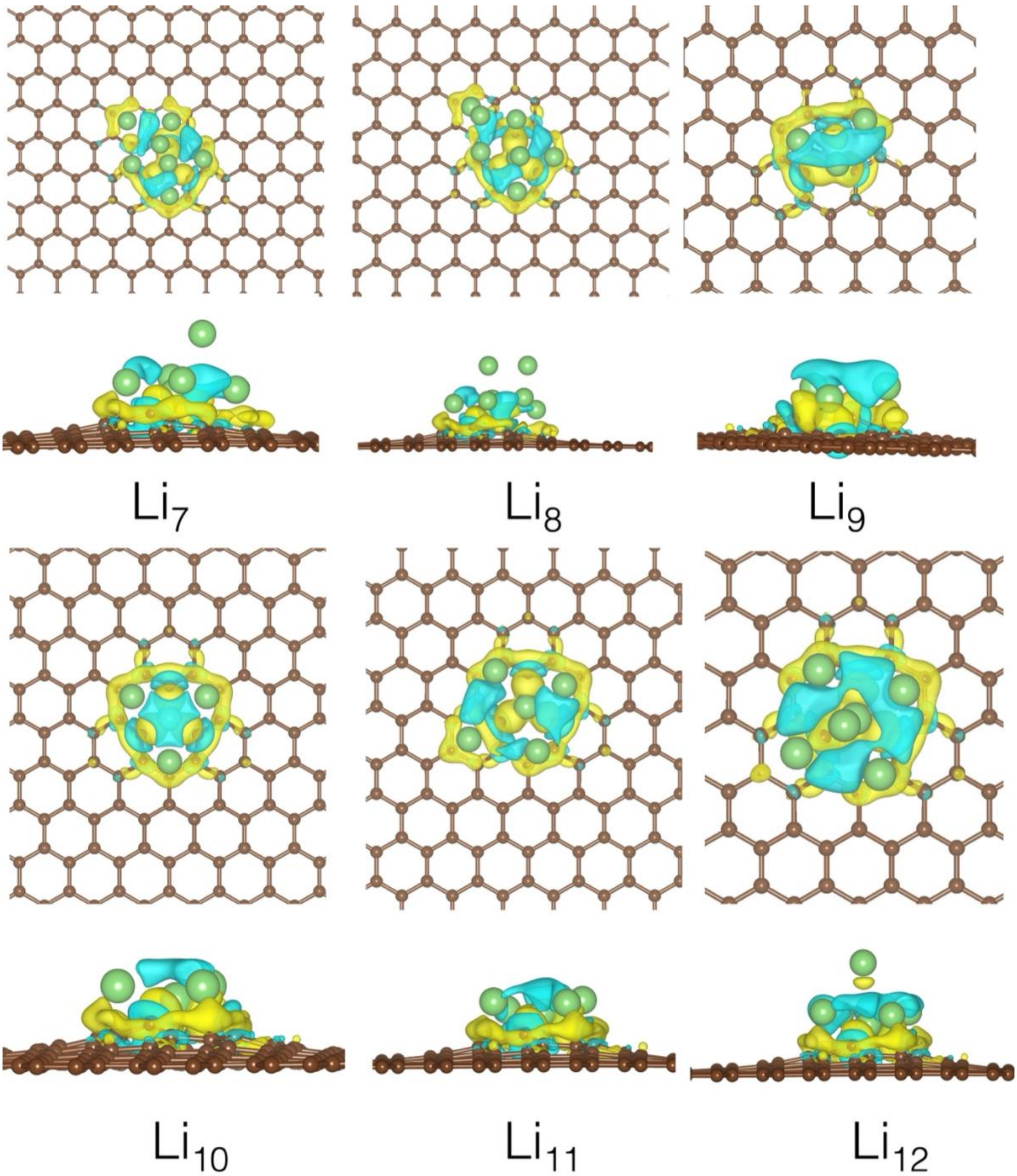

Figure S26. Charge density differences for Li clusters n=1-12 on defective graphene. The first and third rows show the top views, and the second and fourth side views. Li is represented by green spheres and C with brown. Iso-surface is 0.003, with yellow iso-surface denoting gain in charge density, whereas blue denotes loss.

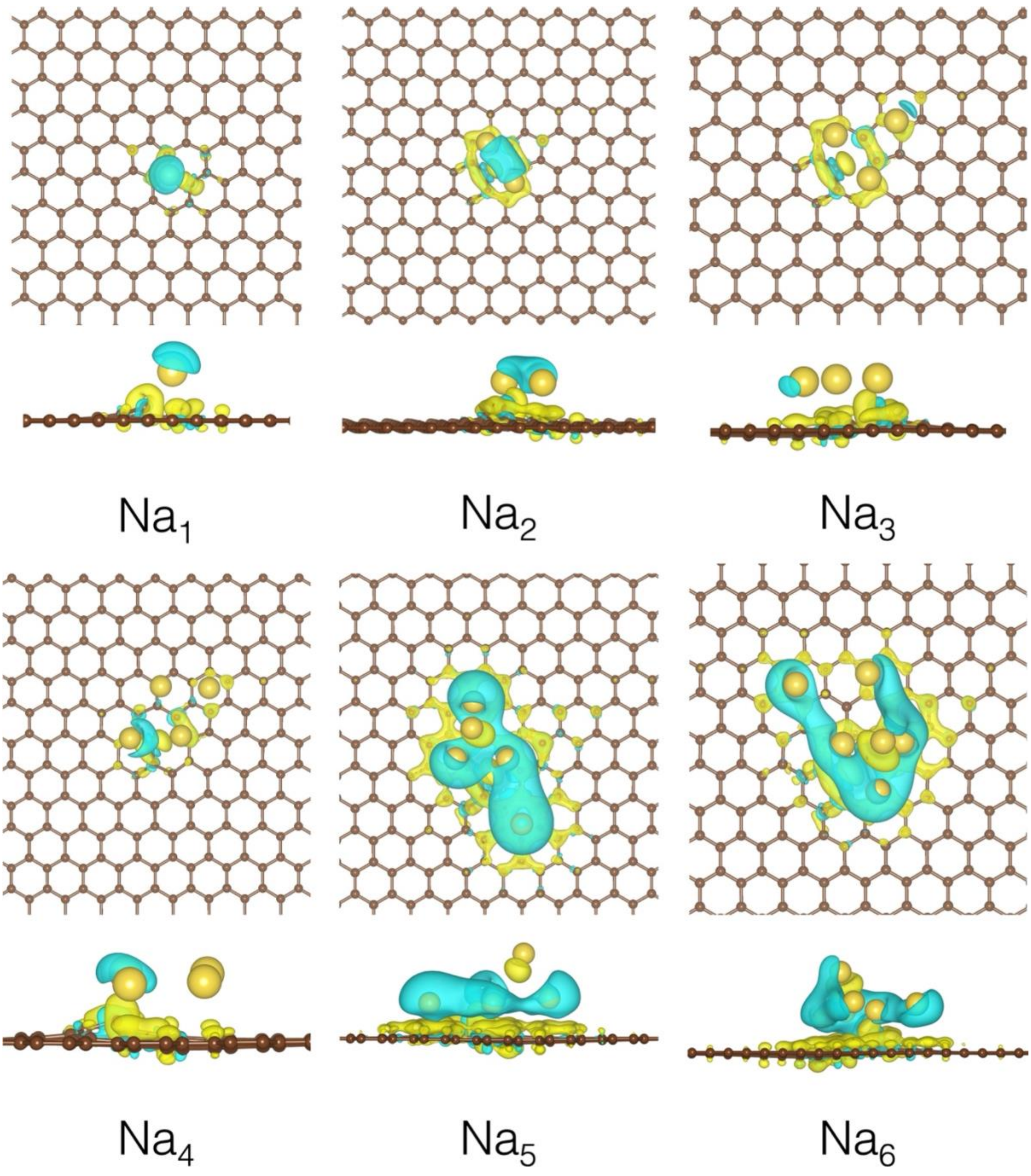
Na1
Na2
Na3
Na4
Na5
Na6

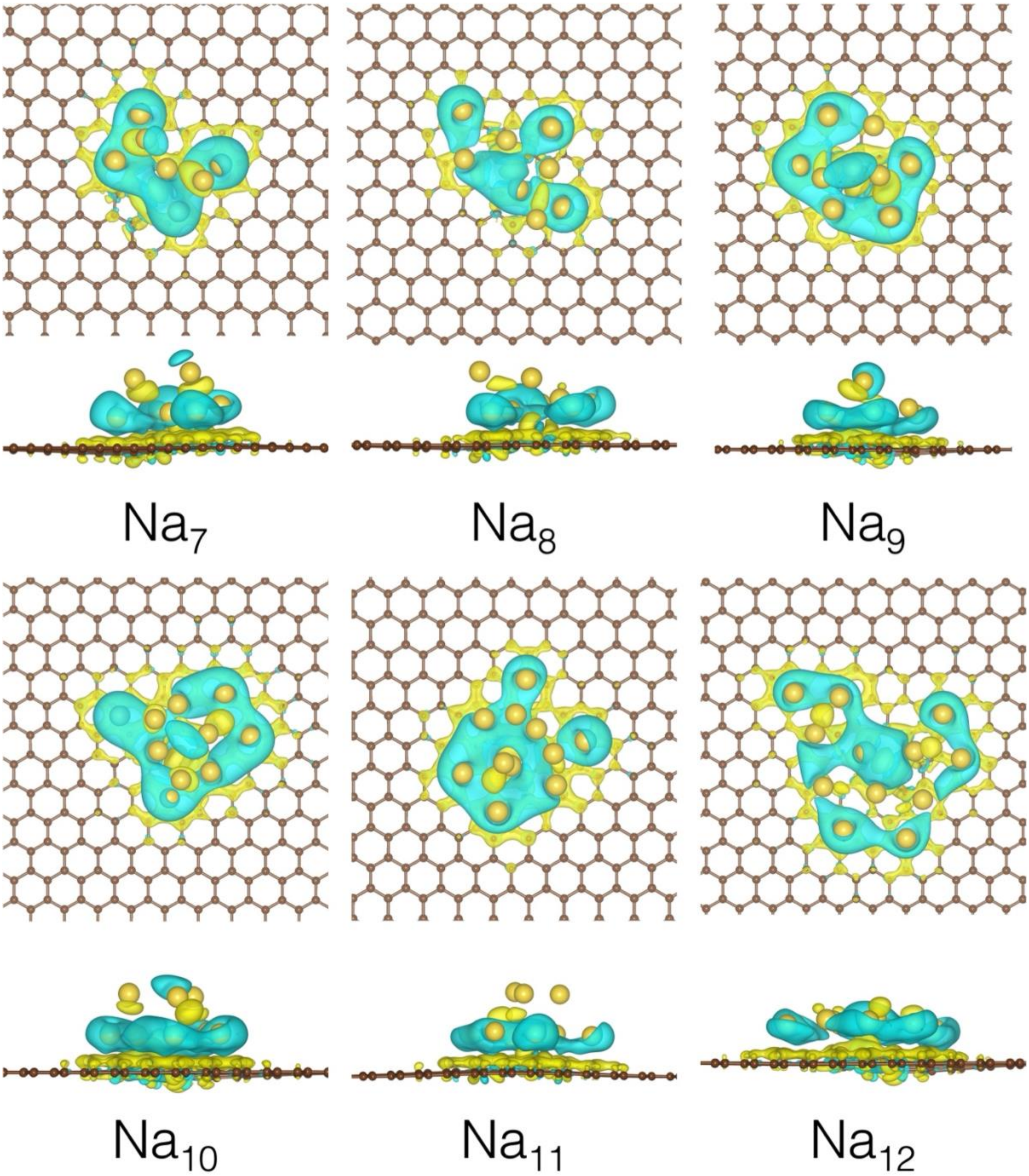

Figure S27. Charge density differences for Na clusters n=7-12 on defective graphene. The first and third rows show the top views, and the second and fourth side views. Na is represented by yellow spheres and C with brown. Iso-surface is 0.0025 for n=1-4, and 0.001 for n=5-12, with yellow iso-surface denoting gain in charge density, whereas blue denotes loss.

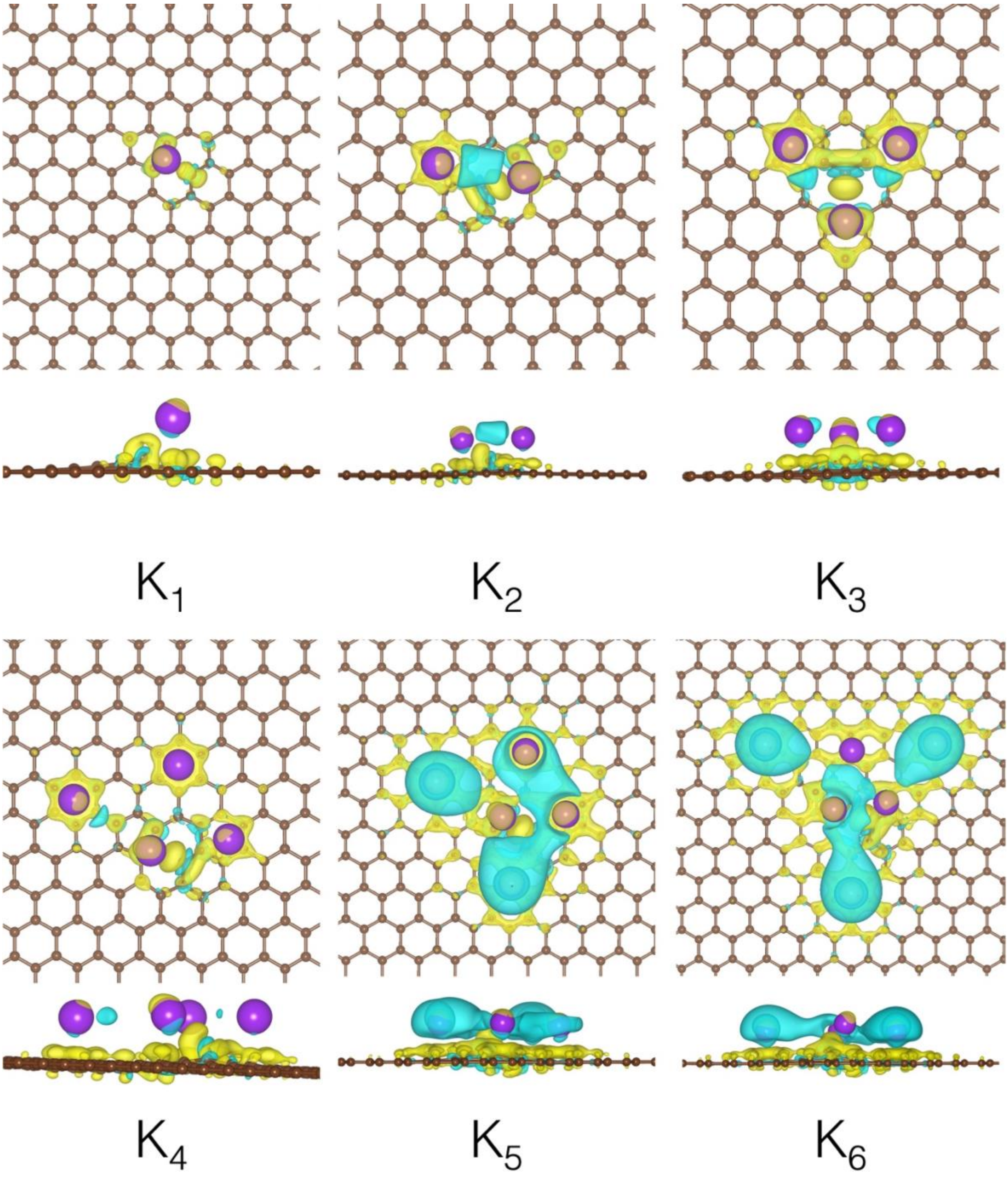
K1
K2
K3
K4
K5
K6

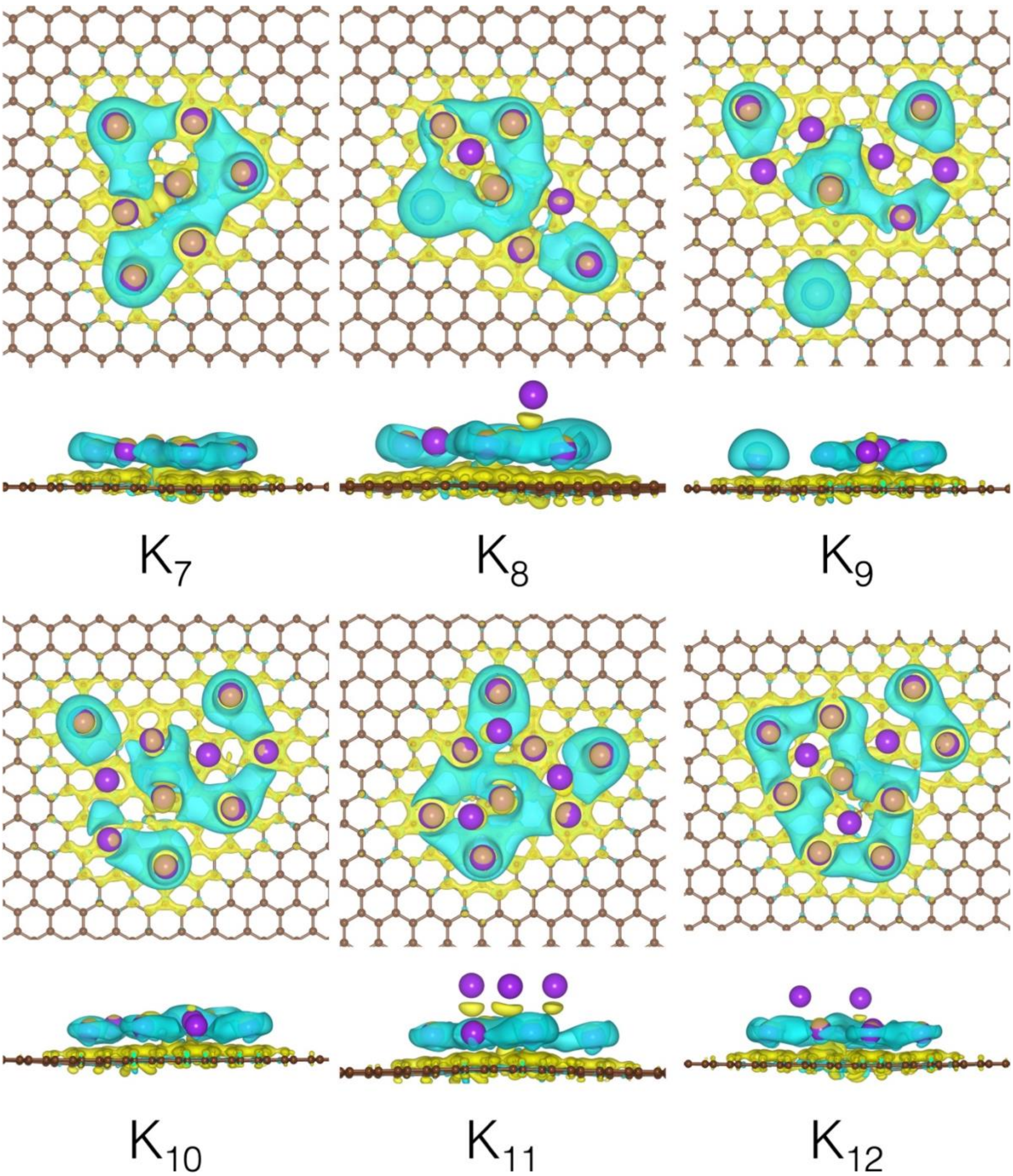

Figure S28. Charge density differences for K clusters n=1-12 on defective graphene. The first and third rows show the top views, and the second and fourth side views. K is represented by purple spheres and C with brown. Iso-surface is 0.002 for n=1-4, and 0.001 for n=5-12with yellow iso-surface denoting gain in charge density, whereas blue denotes loss.
